# Acute blood biomarker profiles predict cognitive deficits 6 and 12 months after COVID-19 hospitalization

**DOI:** 10.1038/s41591-023-02525-y

**Published:** 2023-08-31

**Authors:** Maxime Taquet, Zuzanna Skorniewska, Adam Hampshire, James D. Chalmers, Ling-Pei Ho, Alex Horsley, Michael Marks, Krisnah Poinasamy, Betty Raman, Olivia C. Leavy, Matthew Richardson, Omer Elneima, Hamish J. C. McAuley, Aarti Shikotra, Amisha Singapuri, Marco Sereno, Ruth M. Saunders, Victoria C. Harris, Linzy Houchen-Wolloff, Neil J. Greening, Parisa Mansoori, Ewen M. Harrison, Annemarie B. Docherty®, Nazir I. Lone, Jennifer Quint, Naveed Sattar, Christopher E. Brightling, Louise V. Wain, Rachael E. Evans, John R. Geddes, Paul J. Harrison, PHOSP-COVID Study Collaborative Group*,**, James D. Chalmers, Ling-Pei Ho, Alex Horsley, Michael Marks, Krisnah Poinasamy, Betty Raman, Olivia C. Leavy, Matthew Richardson, Omer Elneima, Hamish J. C. McAuley, Aarti Shikotra, Amisha Singapuri, Marco Sereno, Ruth M. Saunders, Victoria C. Harris, Linzy Houchen-Wolloff, Neil J. Greening, Parisa Mansoori, Ewen M. Harrison, Annemarie B. Docherty, Nazir I. Lone, Jennifer Quint, Naveed Sattar, Christopher E. Brightling, Louise V. Wain, Rachael E. Evans, John R. Geddes

**Affiliations:** 1 Department of Psychiatry, University of Oxford, Oxford, UK; 2 Oxford Health NHS Foundation Trust, Oxford, UK; 3 Department of Brain Sciences, Imperial College London, London, UK; 4 University of Dundee, Ninewells Hospital and Medical School, Dundee, UK; 5 MRC Human Immunology Unit, University of Oxford, Oxford, UK; 6 Division of Infection, Immunity& Respiratory Medicine, Faculty of Biology, Medicine and Health, University of Manchester, Manchester, UK; 7 Manchester University NHS Foundation Trust, Manchester, UK; 8 Department of Clinical Research, London School of Hygiene& Tropical Medicine, London, UK; 9 Hospital for Tropical Diseases, University College London Hospital, London, UK; 10 Division of Infection and Immunity, University College London, London, UK; 11 Asthma and Lung UK, London, UK; 12 Radcliffe Department of Medicine, University of Oxford, Oxford, UK; 13 Oxford University Hospitals NHS Foundation Trust, Oxford, UK; 14 Department of Population Health Sciences, University of Leicester, Leicester, UK; 15 The institute for Lung Health, NIHR Leicester Biomedical Research Centre, University of Leicester, Leicester, UK; 16 NIHR Leicester Biomedical Research Centre, University of Leicester, Leicester, UK; 17 University Hospitals of Leicester NHS Trust, Leicester, UK; 18 Centre for Exercise and Rehabilitation Science, NIHR Leicester Biomedical Research Centre-Respiratory, University of Leicester, Leicester, UK; 19 Department of Respiratory Sciences, University of Leicester, Leicester, UK; 20 Therapy Department, University Hospitals of Leicester, NHS Trust, Leicester, UK; 21 MQ: Transforming Mental Health, London, UK; 22 Centre for Medical Informatics, The Usher Institute, University of Edinburgh, Edinburgh, UK; 23 Usher Institute, University of Edinburgh, Edinburgh, UK; 24 Royal Infirmary of Edinburgh, NHS Lothian, Edinburgh, UK; 25 NHLI, Imperial College London, London, UK; 26 School of Cardiovascular and Metabolic Health, University of Glasgow, Glasgow, UK

## Abstract

Post-COVID cognitive deficits, including ‘brain fog’, are clinically complex, with both objective and subjective components. They are common and debilitating, and can affect the ability to work, yet their biological underpinnings remain unknown. In this prospective cohort study of 1,837 adults hospitalized with COVID-19, we identified two distinct biomarker profiles measured during the acute admission, which predict cognitive outcomes 6 and 12 months after COVID-19. A first profile links elevated fibrinogen relative to C-reactive protein with both objective and subjective cognitive deficits. A second profile links elevated D-dimer relative to C-reactive protein with subjective cognitive deficits and occupational impact. This second profile was mediated by fatigue and shortness of breath. Neither profile was significantly mediated by depression or anxiety. Results were robust across secondary analyses. They were replicated, and their specificity to COVID-19 tested, in a large-scale electronic health records dataset. These findings provide insights into the heterogeneous biology of post-COVID cognitive deficits.

Many people develop neuropsychiatric symptoms in the weeks and months after SARS-CoV-2 infection^1–5^, in isolation or within a post-acute COVID-19 syndrome^6^ also known as long COVID. One in eight patients receives their first ever neurological or psychiatric diagnosis within 6 months following COVID-19 (ref.7). Among these symptoms, cognitive deficits (including ‘brain fog’) are particularly worrisome; they are common^8–10^, persistent^11^ and they affect the ability to work^12^.

How post-COVID-19 cognitive deficits develop remains unknown. Elucidating the mechanisms is a critical step in identifying potential treatments and mitigating the burden of COVID-19. Several hypotheses have been formulated, including endothelial damage, neuroinflammation, thrombotic events, viral invasion and hypoxemia^13–15^. Some of these mechanisms might involve acute pathologies with persistent clinical manifestations, whereas others might only emerge in the post-acute phase^13,16^. Recent animal studies^17–20^ and in vitro analyses^21^ are providing insight into how COVID-19 might affect the brain. Post-COVID-19 autopsies have revealed multifocal vascular damage and microthrombi accompanied by endothelial cell activation^22^.

Other studies have investigated how biological states during the acute phase of COVID-19 predict post-acute outcomes^23–25^. These studies suggest that immunological mechanisms might underpin post-acute COVID-19 conditions; however, they provide little information about the biology of post-COVID cognitive deficits as the latter was either conflated with other conditions into a single post-acute COVID-19 score^23^ or represented as a single self-reported binary (yes/no) variable^24,25^. In contrast, post-COVID cognitive deficits are complex with both objective and subjective components which may or may not impact occupational functioning^26^. It is possible that these different dimensions of ‘brain fog’ are predicted by different biological states and that mechanisms underpinning them differ from those underlying other complications of COVID-19.

Here we used data from a large prospective longitudinal cohort study (the Post-hospitalization COVID-19 (PHOSP-COVID) study; ISCTN Registry no. ISRCTN10980107) to discover patterns of association between biomarkers measured on admission to hospital for COVID-19 and post-acute cognitive deficits (measured 6 and 12 months later). Both objective and subjective cognitive deficits, as well as occupational impact, were measured. We used canonical correlation analysis (CCA), an approach employed increasingly in biomedical research to discover patterns of covariation between sets of variables^27–29^. The generalizability of the findings was tested by seeking to reproduce them in a separate population using electronic health records (EHR) data from over 90 million patients.

## Results

A total of 1,837 patients (mean (s.d.) age, 57.9 (12.4); 36.6% female, 57.7% male) were part of the PHOSP-COVID cohort (baseline characteristics in Table 1, first column and Supplementary Table 1).

### Factors associated with post-COVID cognitive deficits

**Fig. 1:**
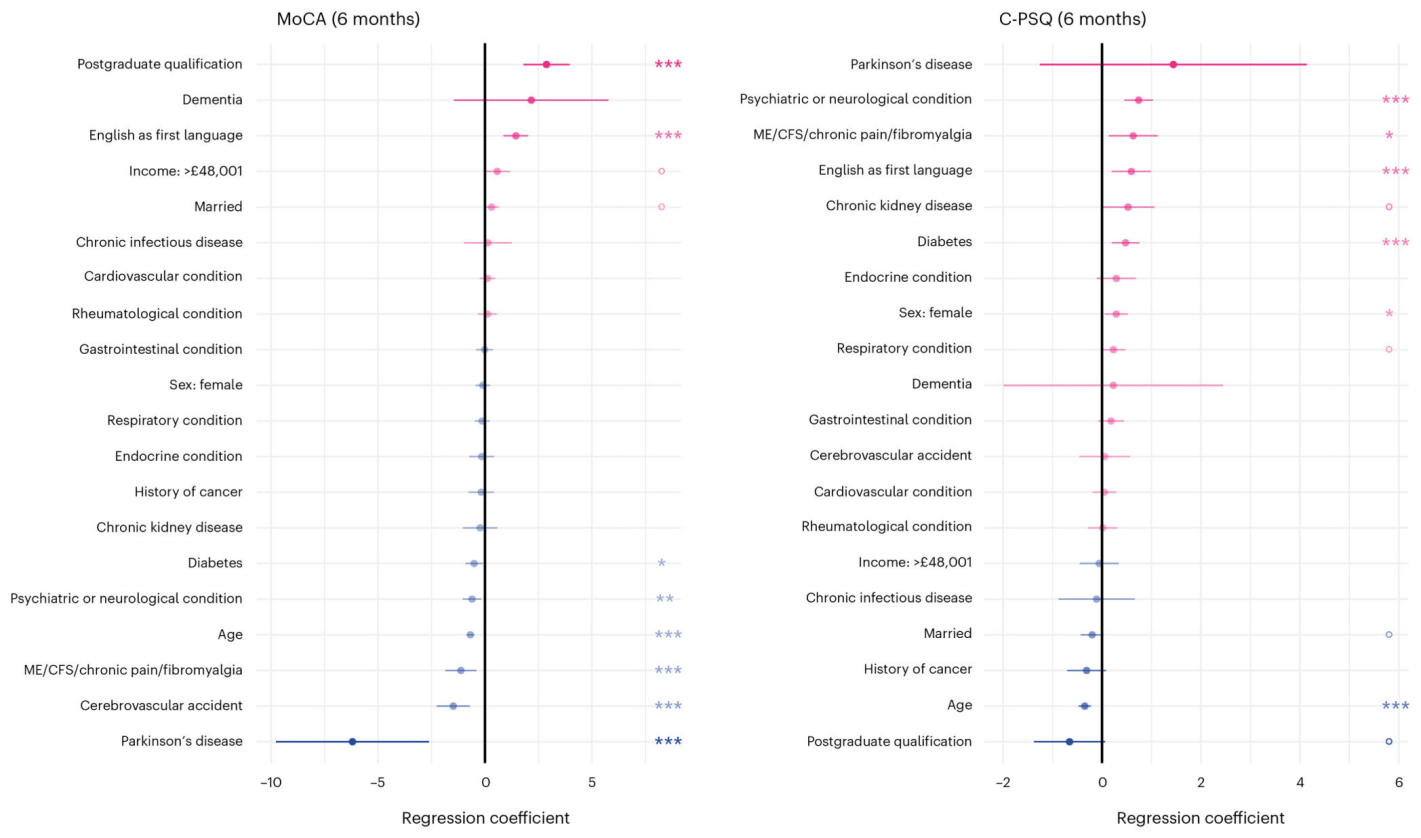
Factors associated with post-COVID cognitive deficits. Association between baseline characteristics and MoCA at 6 months (measuring objective cognitive deficits, lower indicate more deficits) and C-PSQ at 6 months (measuring subjective cognitive deficits, higher means more deficits). Age was z-transformed in this analysis which means that the coefficient corresponds to a difference in MoCA/C-PSQ corresponding to a difference of 1 × s.d. in age. Only one level of education and one income level are presented (with no education and income <£19,000 being taken as references respectively). The same graphs with all education and income levels, as well as ethnicity, are presented in Supplementary Figs. 1 and 2. *n* = 1,837 individual participants. Dots indicate point estimates and horizontal lines indicate 95% CI. *P* values were estimated as part of a generalized linear model and are two-sided and not adjusted for multiple comparisons: *P* < 0.1, *P < 0.05, **P < 0.01, ***P < 0.001. NVQ, national vocational qualification.

The Montreal Cognitive Assessment (MoCA) score (a measurement of objective cognitive deficits) at 6 months was significantly associated with a range ofbaseline characteristics, including age, education level and several comorbidities (Fig.1 and Supplementary Fig. 1). The cognitive items of the Patient Symptom Questionnaire (C-PSQ^5,30^, a measurement of subjective cognitive deficits) were also associated with a range of baseline characteristics including age and comorbidities (especially psychiatric or neurological conditions and chronic fatigue syndrome (CFS)/chronic pain/fibromyalgia; Fig.1 and Supplementary Fig. 2). Younger participants and those whose first language is English had significantly worse C-PSQ but better MoCA. All these variables were included as covariates in subsequent analyses (whether they were significantly associated with cognitive outcomes or not).

### Two dimensions link biomarkers with cognitive profiles

CCA was used to identify dimensions of covariation linking a set of six blood biomarkers measured on admission to hospital (C-reactive protein (CRP), D-dimer, fibrinogen, lymphocyte, neutrophil and platelet counts; these represent various aspects of health, including inflammation, coagulation and immune system reaction) with a set of 14 cognitive scores measured 6 months later (seven individual items of the MoCA and seven individual items of the C-PSQ). All biomarker and cognitive values were adjusted for all covariates described in the previous section before being input to CCA. Each dimension consists of one linear combination of biomarkers (referred to as a biomarker profile) and one linear combination of cognitive scores (referred to as a cognitive profile) such that the biomarker and cognitive profiles are highly correlated.

We identified two statistically significant dimensions of covariation (*r* = 0.23 and *r* = 0.17, with *P* < 0.0001 and *P* = 0.0010, respectively, corrected for multiple comparisons by recording maximum correlations within a permutation test; all other dimensions had *P* > 0.05). These dimensions were robust in split-sample analysis wherein the population was randomly split in half 100 times (mean correlation in weights between original and split samples in the first dimension: 0.87 for biomarkers and 0.88 for cognitive scores; in the second dimension, 0.77 for biomarkers and 0.71 for cognitive scores; permutation test *P* < 0.001 for both dimensions and for both biomarkers and cognitive scores). The dimensions were also robust in leave-one-out cross-validation (*r* = 0.18 and *r* = 0.11, both *P* < 0.0001) and when the data were limited to complete cases, with no imputation (first dimension: *r* = 0.22, *P* < 0.0001; second dimension: *r* = 0.14, *P* = 0.008, neither was significantly different from the original dimensions: *P* > 0.6).

### High fibrinogen is linked with objective and subjective cognitive deficits

**Extended Data Fig. 1:**
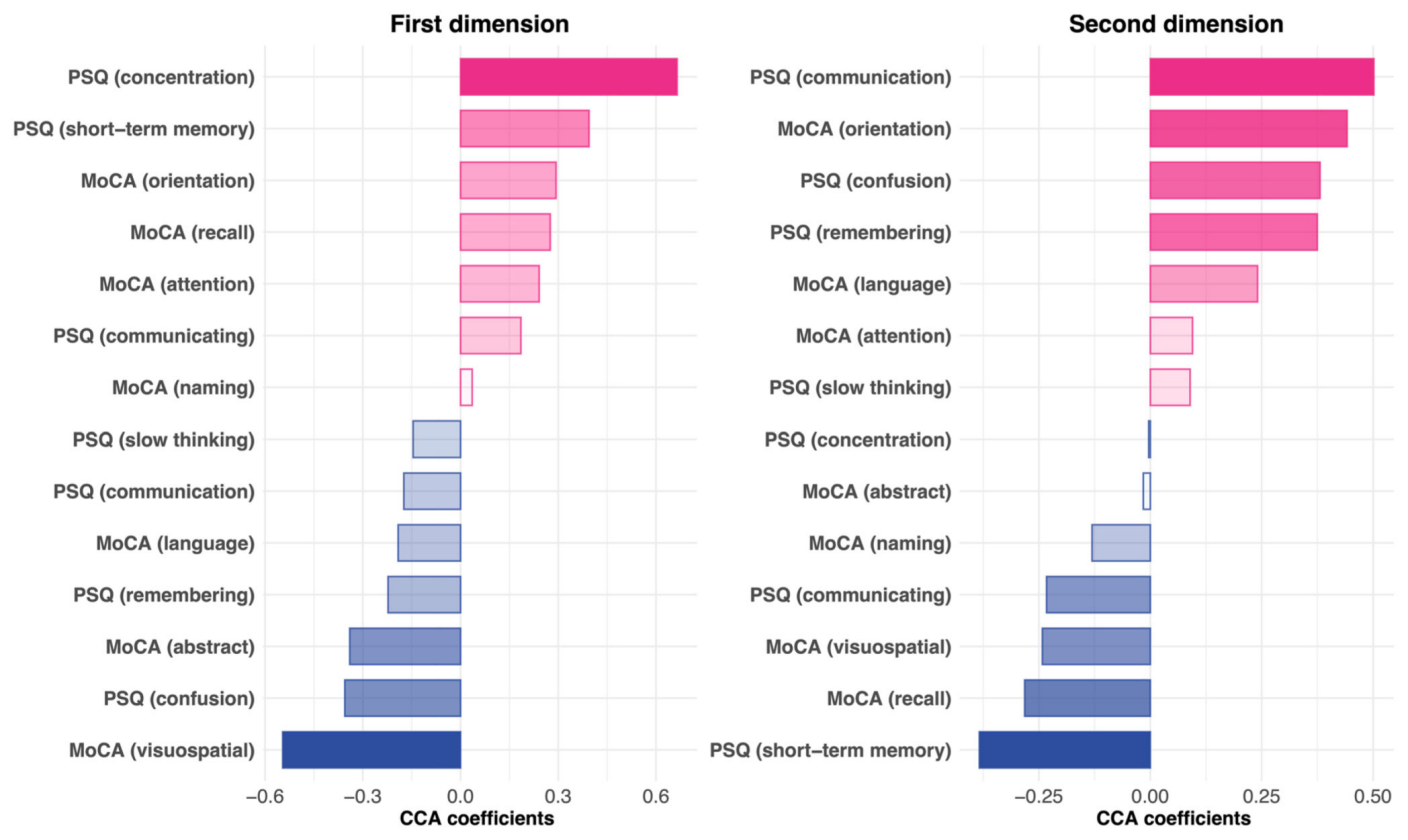
Contributions of each cognitive item to the dimensions of covariation. These report the weights of each item in the weighted combinations that represent the cognitive profile for each dimension. Note that positive weights (for MoCA items) and negative weights (for C-PSQ items) do not necessarily imply that individuals who scored high on the dimension of covariation had better cognitive outcomes for those items since these items might covary with other items with opposite weights. See Supplementary Figs. 5–6, 9 and 10 for distribution of individual items along dimensions of covariation.

**Fig. 2:**
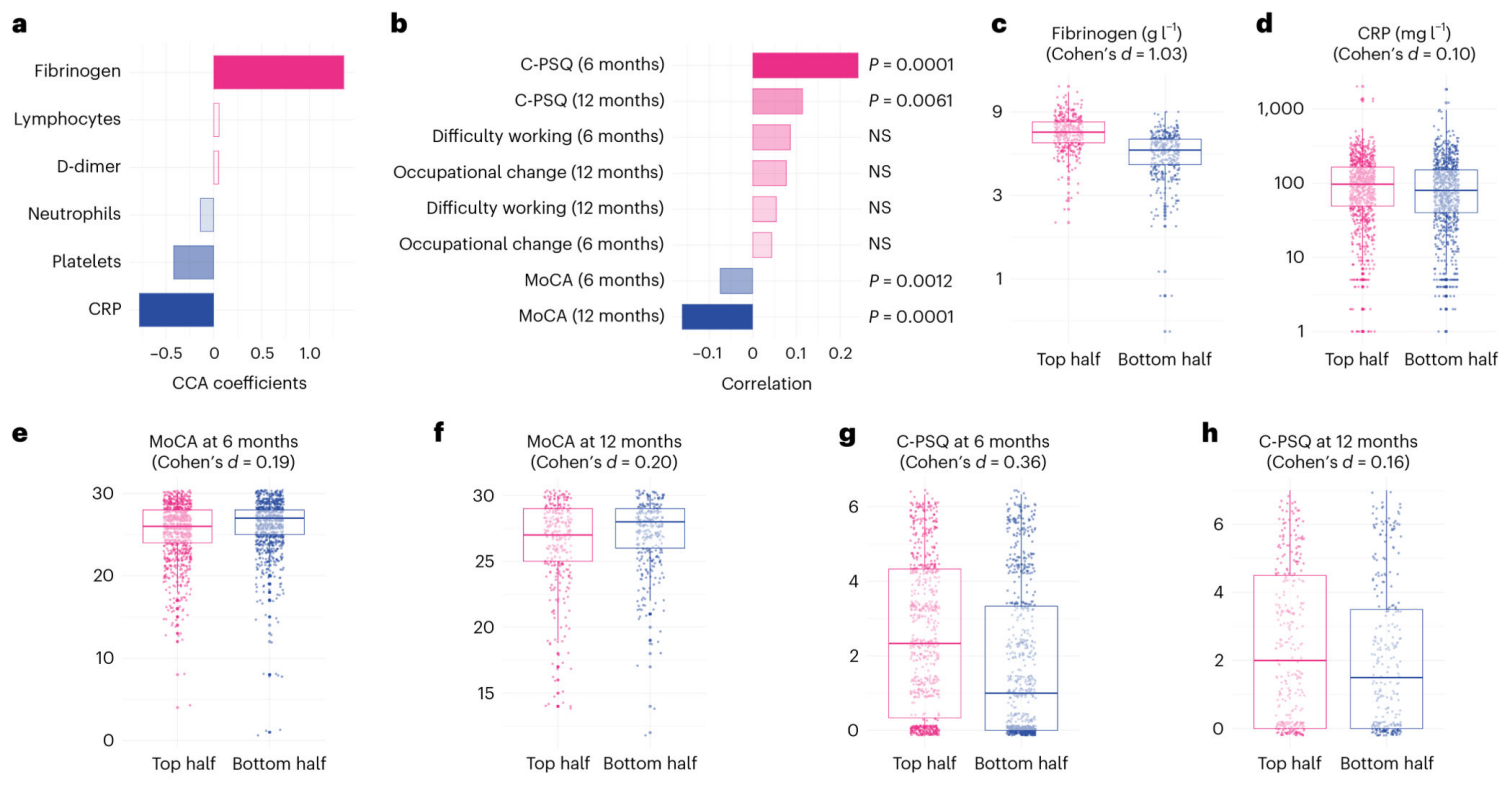
High fibrinogen is linked with objective and subjective cognitive deficits. **a**,**b**, A first dimension of covariation links high fibrinogen with relatively low CRP to higher C-PSQ at 6 and 12 months (signs of subjective cognitive deficits) and lower MoCA at 6 and 12 months (signs of objective cognitive deficits). *P* values are derived from permutation tests, two-sided and not corrected for multiple comparisons. **c**–**h**, Distribution of different variables between the top half and the bottom half of the cohort along this first dimension (*n* = 768, 1,777, 1,837, 626, 1,502 and 584 individual participants, respectively). The center of the boxes represents the median, their bounds represent the 25th and 75th centiles and the lower and upper ends of whiskers represent the smallest/largest values, no further than 1.5 × IQR from the box-plot respective end. Distribution of all variables investigated is found in Supplementary Figs. 4–6. NS, *P* > 0.05.

On the biomarker side, the first dimension of covariation was characterized by a positive weight for fibrinogen and a negative weight for CRP (Fig. 2a and Supplementary Table 2). This indicates that the first dimension of covariation was driven by elevated fibrinogen with a CRP level that was not as high as the fibrinogen would suggest (elevated fibrinogen relative to CRP) given that the two tend to be correlated at the cohort level (Fig. 2a and Supplementary Table 2). On the cognitive side, this dimension of covariation was driven by a range of deficits across objective and subjective domains (Extended Data Fig. 1), which translated into significantly higher C-PSQ (subjective cognitive deficit) and lower MoCA score (objective cognitive deficit) at 6 months after COVID-19 (Fig. 2b and Supplementary Table 3). This cognitive profile was also associated with significantly lower MoCA scores and significantly higher C-PSQ at 12 months, but not with differences in occupational outcomes (Fig. 2b). In other words, individuals with high fibrinogen relative to CRP on admission tend to have signs of objective and subjective cognitive deficits at 6 and 12 months after COVID-19.

**Extended Data Fig. 2:**
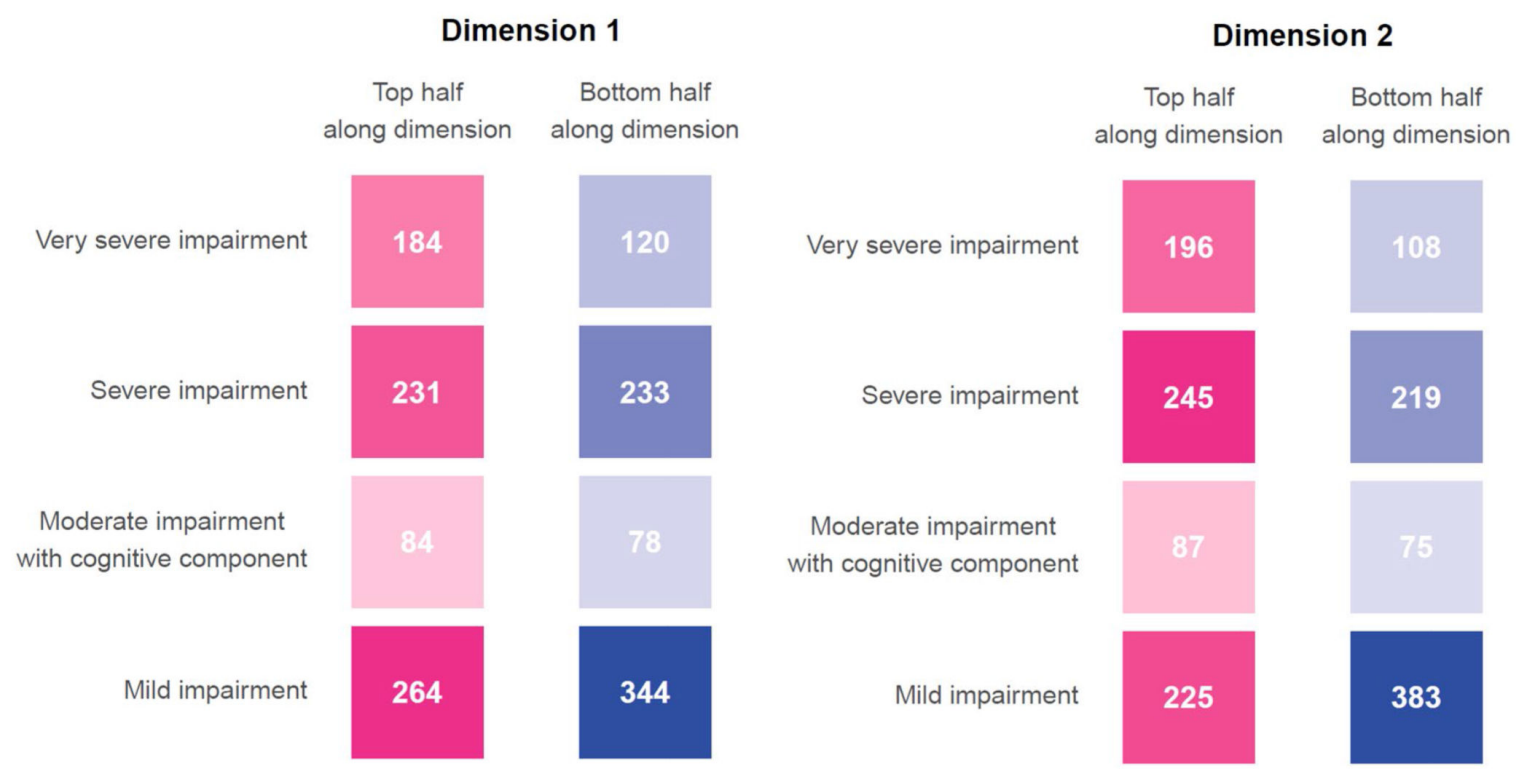
Association between dimensions of covariation and clusters of post-acute impairment. Distribution of individuals in the top and bottom half of the cohort along both dimensions of covariation in terms of predefined clusters of post-acute impairment. For both dimensions, those who scored in the top half of the cohort tended to have more severe impairment.

The effect sizes of the association can be appreciated by comparing those in the top vs. bottom half of the cohort along the first dimension of covariation. These two sub-cohorts had similar baseline characteristics (Table 1, middle columns). Those in the top half of the cohort along this first dimension had elevated fibrinogen levels compared to those in the bottom half (mean (95% confidence interval (CI)) 6.82 (6.72–6.92) versus 5.09 (4.99–5.20) g l^-1^; Cohen’s *d*, 1.03; Fig. 2c) and similar CRP levels (mean (95% CI) 76.8 (71.3–82.7) versus 68.2 (63.3–73.5) mg l^-1^; Cohen’s *d*, 0.10; Fig. 2d). They had lower MoCA at 6 months (25.35 versus 26.01; difference in mean 0.66, 95% CI 0.34–0.98; Fig. 2e) and 12 months (26.22 versus 26.85; difference in mean 0.63, 95% CI 0.13–1.12; Fig. 2f) and higher C-PSQ at 6 months (2.52 versus 1.79; difference in mean 0.72, 95% CI 0.52–0.93; Fig. 2g) and 12 months (2.27 versus 1.93; difference in mean 0.34, 95% CI 0.0009–0.68; Fig. 2h). Predefined clusters characterized by different degrees of post-acute impairment were unevenly distributed along this dimension: those in the top half of the cohort had more severe post-acute impairment (odds ratio (OR) for being severely impaired: 1.73, 95% CI 1.34–2.24, *P* < 0.0001; Extended Data Fig. 2). Supplementary Fig. 3 shows the correlations between subjective and objective cognitive deficits and occupational outcomes and Supplementary Figs. 4–6 show other variables separated between top and bottom halves of the cohort along the first dimension. No robust association was found between the cognitive profile of this first dimension and biomarkers measured at the 6-month follow-up (Supplementary Fig. 7).

### High D-dimer is linked with subjective cognitive deficits and occupational outcomes

**Fig. 3:**
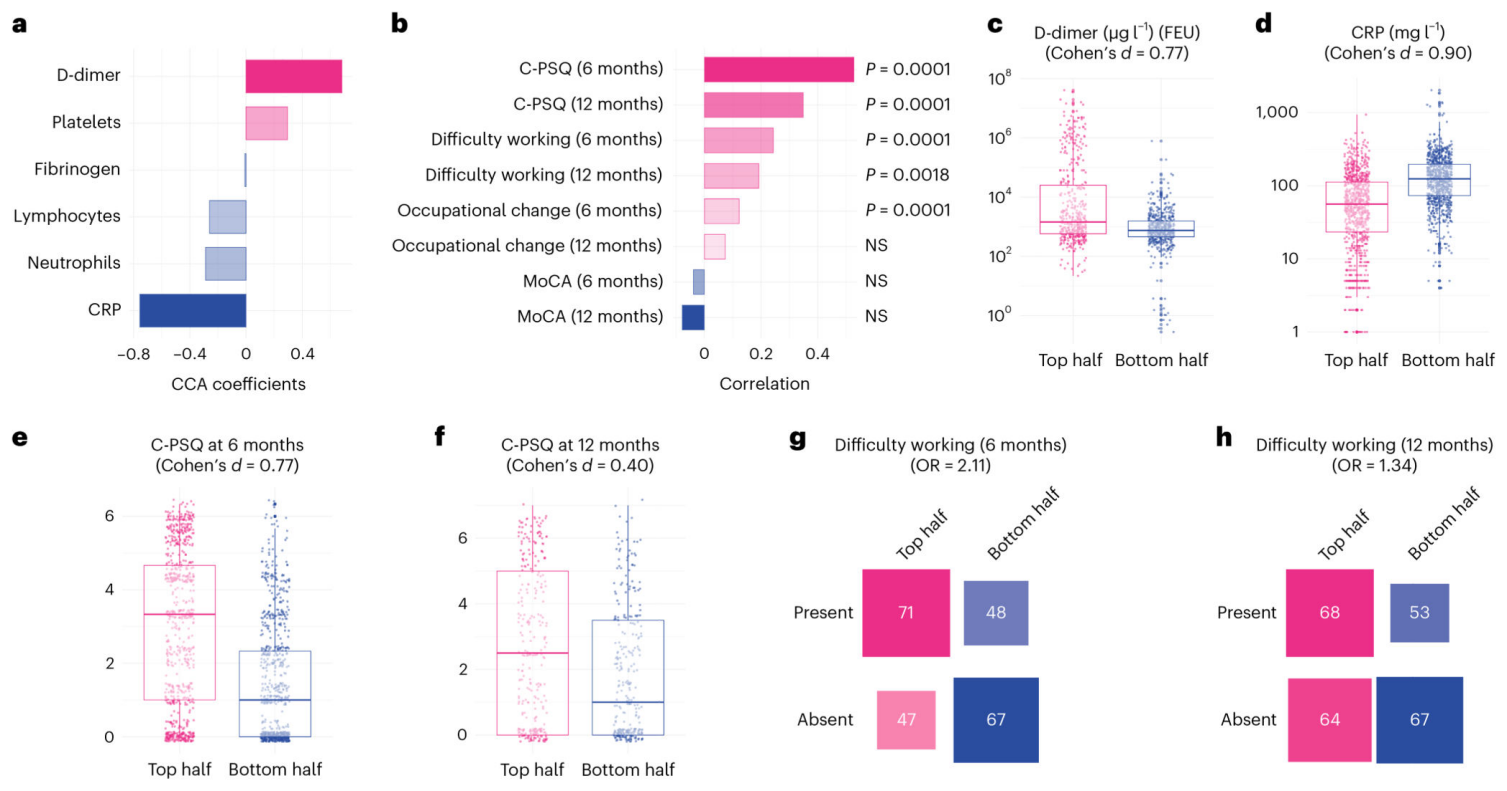
High D-dimer is linked with subjective but not objective cognitive deficits. **a**,**b**, A second dimension of covariation links high D-dimer with relatively low CRP to higher C-PSQ at 6 and 12 months (signs of subjective cognitive deficits) and signs of occupational impact (in terms of affected ability to work at 6 and 12 months and occupational changes at 6 months) but little difference in MoCA. *P* values are derived from permutation tests, two-sided and not corrected for multiple comparisons. **c**–**h**, Distribution of different variables between the top half and the bottom half of the cohort along this second dimension (*n* = 977, 1,777, 1,502, 584, 233 and 252 individual participants, respectively). The center of the boxes represents the median, their bounds represent the 25th and 75th centile and the lower and upper ends of whiskers represent the smallest/largest value, no further than 1.5 × IQR from the boxplot respective end. Distribution of all variables investigated can be found in Supplementary Figs. 8–10. NS, *P* > 0.05.

On the biomarker side, the second dimension of covariation was driven by elevated D-dimer relative to CRP (Fig. 3a and Supplementary Table 4). On the cognitive side, it was driven by a range of deficits across domains (Extended Data Fig. 1), which translated into significantly higher C-PSQ (more subjective impairment), but not lower MoCA, at 6 months after COVID-19 (Fig. 3b and Supplementary Table 5). This cognitive profile was also significantly correlated with higher C-PSQ at 12 months and with occupational outcomes at 6 and 12 months (Fig. 3b). In other words, individuals with high D-dimer relative to CRP tend to have subjective cognitive deficits, as well as signs of occupational impact, at 6 and 12 months.

Those in the top half of the cohort along this dimension had very similar baseline characteristics as those in the bottom half (Table 1; right columns). Compared to those in the bottom half of the cohort, those in the top half had elevated D-dimer (mean (95% CI) 4.97 × 10^3^ (4.06-6.07) versus 0.78 × 103 (0.70-0.86) μg l^-1^ fibrinogen equivalent units (FEU); Cohen’s *d*, 0.77; Fig. 3c), lower CRP (mean (95%CI) 44.8 (41.3-48.6) versus 115.7 (109.9-121.8) mg l^-1^; Cohen’s *d*, 0.90; Fig.3d), higher C-PSQ at 6 months (2.90 versus 1.42; difference in mean 1.48, 95% CI 1.29-1.68; Fig. 3e) and higher C-PSQ at 12 months (2.51 versus 1.69; difference in mean 0.82, 95% CI 0.48-1.16; Fig. 3f). They were more likely to report impaired ability to work at 6 months (OR = 2.11, 95% CI 1.25-3.56; Fig.3g) and 12 months (OR = 1.34, 95% CI 0.82-2.21; Fig.3h) and to report occupational change at 6 months (OR = 1.57, 95% CI 1.21-2.05) but not 12 months (OR = 0.91, 95% CI 0.59-1.39). As for the first dimension, those in the top half were characterized by more severe post-acute impairment based on predefined clusters (OR for being severely impaired: 2.20, 95% CI 1.70-2.87, *P* < 0.0001; Extended Data Fig. 2). Supplementary Figs. 8-10 show other variables separated between top and bottom halves of the population along the second dimension. No robust association was found between the cognitive profile of this second dimension and biomarkers measured at the 6-month follow-up (Supplementary Fig. 7).

### Evidence of absence of confounding by pre-COVID cognition

If pre-COVID cognitive function predicts both acute biomarker profiles and post-COVID cognitive deficits, then it might confound the associations identified. We tested this possibility in three different ways using data from a subgroup of the PHOSP-COVID cohort who reported their subjective cognitive function both before and at 6 months (*n* = 547) and 12 months (*n* = 205) after COVID using C-PSQ-2 (a subset of items from C-PSQ).

**Extended Data Fig. 3:**
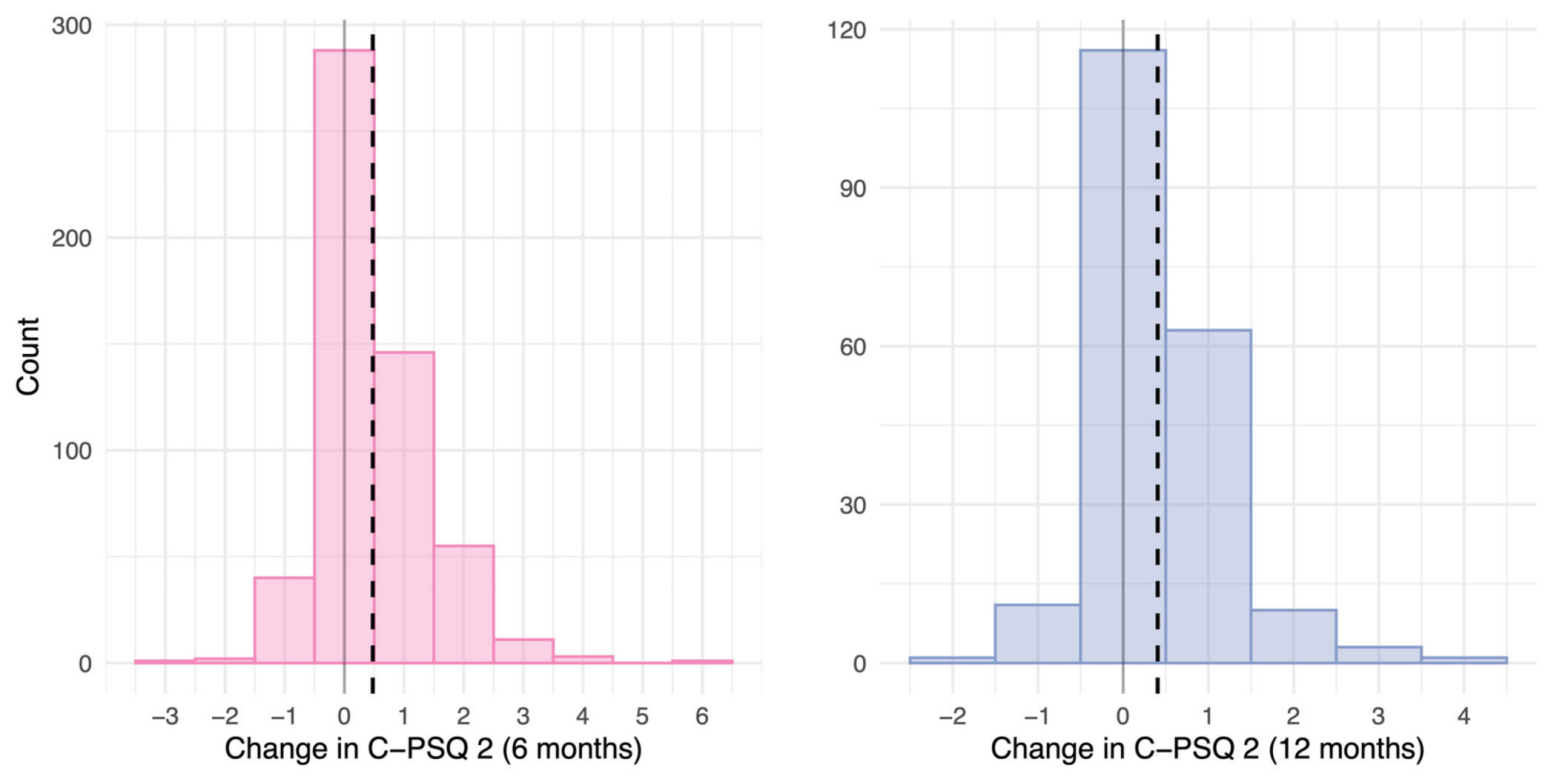
Change in subjective cognitive function between pre- and post-COVID. Distribution of the change in C-PSQ-2 (assessing subjective cognitive deficits) between a pre-COVID baseline and 6 months (left) or 12 months (right) post-COVID. The dashed lines represent the mean change. In both cases, the change was, on average, significantly greater than zero indicating worsening of subjective cognitive function following COVID-19 (mean [s.e.m.] change in C-PSQ-2: 0.48 [0.04] between pre-COVID and 6 months post-COVID, p < 0.0001; and 0.40 [0.055] between pre-COVID and 12 months post-COVID-19, p < 0.0001). At six months, 43/547 participants (7.9%) had better cognition, 288 (52.7%) had no change, and 216 (39.5%) had worse cognition compared to before COVID-19. At 12 months, 12/205 participants (5.9%) had better cognition, 116 (56.6%) had no change, and 77 (37.6%) had worse cognition compared to before COVID-19.

We first assessed whether cognitive deficits at 6 and 12 months merely reflected pre-existing cognitive deficits by testing whether there were significant changes in C-PSQ-2 between before and after COVID-19. Cognitive function was found to deteriorate on average following COVID-19 (mean (s.e.m.) change in C-PSQ-2: 0.48 (0.04) between before COVID and 6 months after COVID, *P* < 0.0001 and 0.40 (0.055) between before COVID and 12 months after COVID-19, *P* < 0.0001; Extended Data Fig. 3).

Second, we assessed whether pre-existing cognitive deficits predicted biomarker profiles, which would be necessary to confound the associations. Pre-existing cognitive deficits were not associated with either biomarker profile (*r* = 0.043, 95% CI -0.05-0.14, *P* = 0.36 in the first dimension and *r* = 0.022, 95% CI -0.071-0.12, *P* = 0.64 in the second dimension). This provides evidence that high fibrinogen or high D-dimer levels relative to CRP are not more commonly observed in people with pre-existing cognitive deficits.

Third, we assessed whether dimensions of covariation are associated with changes in cognitive function from a pre-COVID baseline. Given the relative contributions of C-PSQ items to the dimensions of covariation (Extended Data Fig. 1), one can anticipate that C-PSQ-2 at 6 months would be associated with the second but not the first dimension of covariation. This was confirmed (correlation between C-PSQ-2 and the second dimension of covariation: *r* = 0.22, 95% CI 0.12–0.31, *P* = 0.0002 at 6 months and *r* = 0.20, 95% CI 0.048–0.34, *P* = 0.011 at 12 months; correlation with the first dimension of covariation: *r* = 0.063, 95% CI -0.038–0.16, *P* = 0.23 at 6 months and *r* = 0.03, 95% CI -0.12–0.18, *P* = 0.72 at 12 months). C-PSQ-2 thus captures subjective cognitive deficits experienced by those scoring high along the second dimension of covariation. We found that those scoring higher along that dimension had significantly worse changes in cognitive function (*r* = 0.16, 95% CI 0.061–0.26, *P* = 0.0021 for the change at 6 months and *r* = 0.27, 95% CI 0.13–0.41, *P* = 0.0005 for the change at 12 months).

These complementary analyses indicate that associations between biomarker profiles and subjective cognitive deficits cannot be explained by pre-COVID cognitive function.

### Mediation by clinical features and severity of acute illness

**Fig. 4:**
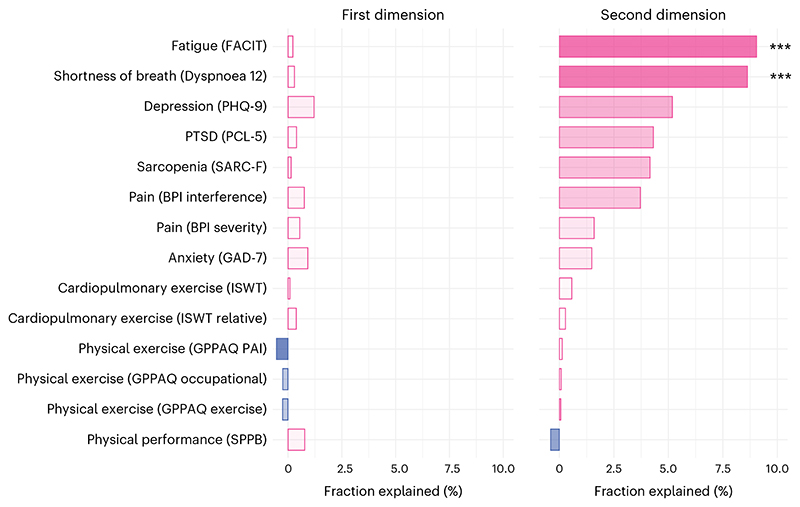
Mediation by other clinical features. Mediation of the associations captured in the first and second dimensions of covariation by scales representing other aspects of health at 6 months after COVID-19. The names of the scales are reported in brackets. *P* values were estimated using nonparametric bootstrap with 1,000 repetitions and are two-sided and not adjusted for multiple comparisons: ***P < 0.001. For the second dimension, the *P* value for fatigue was 0.004 and for shortness of breath it was <0.001 (below the minimum threshold detectable with 1,000 repetitions). FACIT, Functional Assessment of Chronic Illness Therapy; BPI, Brief Pain Inventory; SARC-F, Sarcopenia screen; ISWT, Incremental Shuttle Walk Test; GPPAQ, General Practice Physical Activity Questionnaire; SPPB, Short Physical Performance Battery; PHQ-9, Patient Health Questionnaire; PTSD, post-traumatic stress disorder; PCL-5, PTSD Checklist; GAD-7, Generalized Anxiety Disorder scale; PAI, Physical Activity Index.

The association captured by the first dimension was not significantly mediated by any of 14 clinical scales 6 months after COVID (capturing fatigue, dyspnea, exercise tolerance, pain, depression and anxiety), whereas the association between the biomarker and cognitive profiles in the second dimension was significantly mediated by dyspnea (fraction explained, 8.63%, *P* < 0.001) and fatigue (fraction explained, 9.05%, *P* = 0.004; Fig. 4 and Supplementary Table 6).

**Extended Data Fig. 4:**
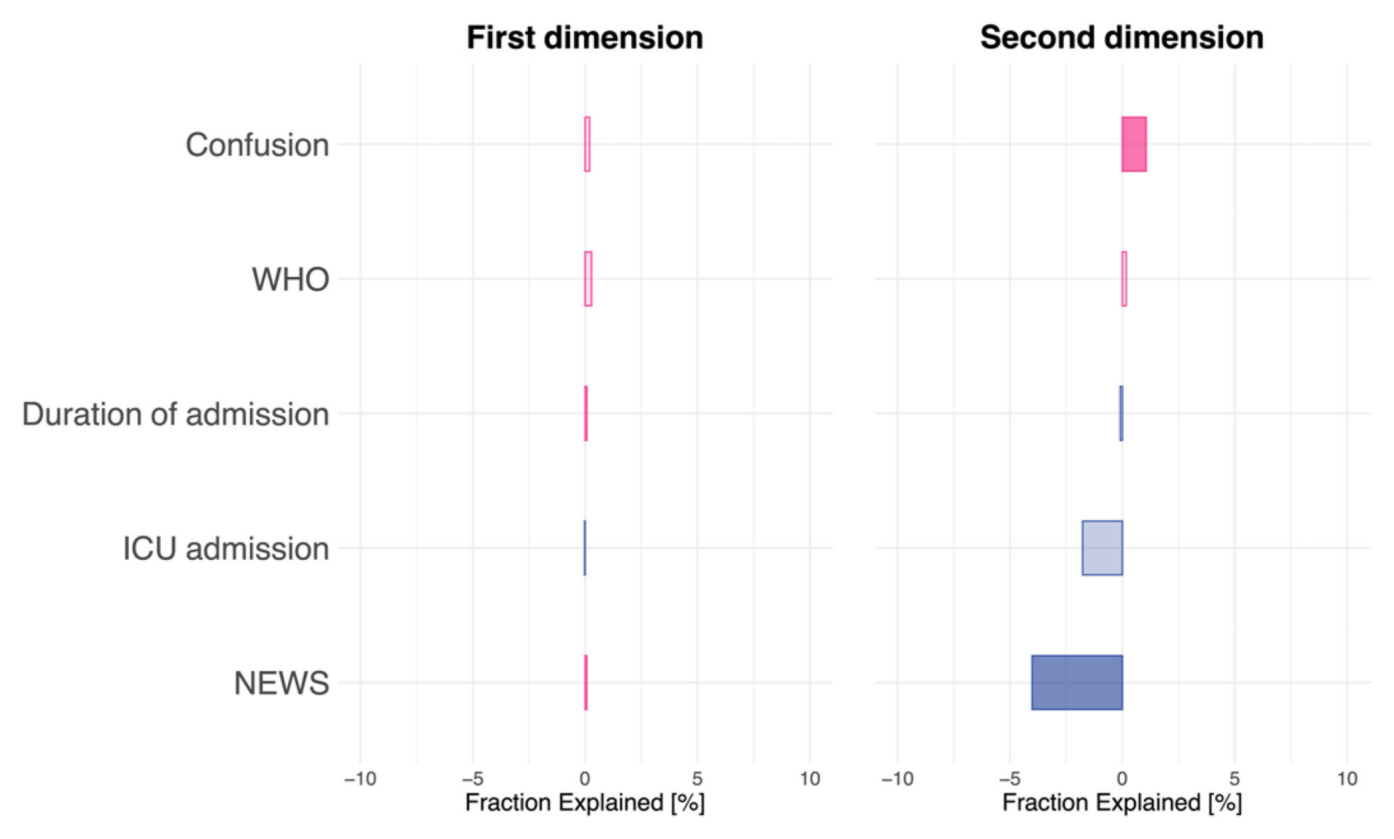
Mediation of the associations between biomarker and cognitive profiles by markers of severity of infection. No marker reached statistical significance. Fraction explained by the mediator are reported as negative if they are negatively associated with the cognitive profile. WHO, World Health Organization clinical progression scale; ICU, Intensive care unit; NEWS, National Early Warning Scores.

The associations captured by both dimensions were not significantly mediated by severity of the acute illness (captured by a range of severity markers; Extended Data Fig. 4) so that they remained significant when all mediators were included in the model (direct effect for the first dimension, *β* = 0.26, 95% CI 0.21–0.32, *P* < 0.001 and for the second dimension, *β* = 0.18, 95% CI 0.11–0.24, *P* < 0.001).

### Independent replication in a large-scale EHR network

To assess the generalizability of the main findings, we reproduced the analysis using an independent and structurally different dataset, namely the TriNetX Analytics Network, an EHR network of 57 healthcare organizations primarily in the United States covering over 90 million patients^2,3^. Within this dataset, all individuals hospitalized with COVID-19 were identified and the risk of post-COVID cognitive deficits (captured with a range of ICD-10 codes as used in previous studies^2,3,31^) was compared between subgroups within that cohort. Subgroups were propensity-score-matched for 82 covariates capturing risk factors for COVID-19, for more severe COVID-19 illness^32^ and for COVID-19 neurological and psychiatric sequelae^2,3^, as well as vaccination status.

**Extended Data Fig. 5:**
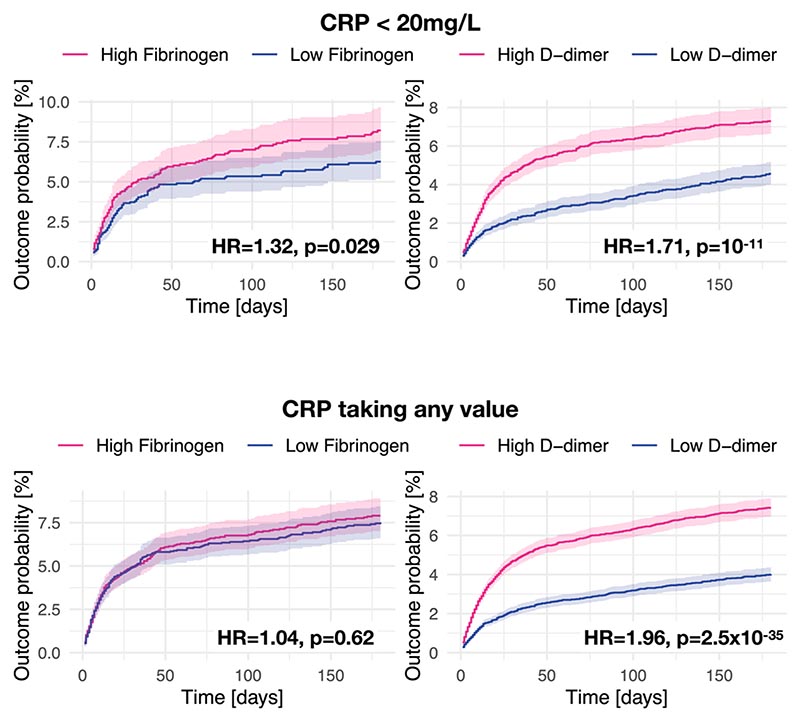
Associations between biomarkers and post-acute cognitive deficits in the EHR data with different constraints on CRP. Kaplan– Meier curves represent the cumulative incidence of cognitive deficits between those with high versus low fibrinogen (or D-dimer) and CRP level ≤ 20 mg/L (top panels), or any CRP level (bottom panels). Curves represent the Kaplan– Meier estimates and shading around curves represents 95% confidence intervals. P-values are derived from log-rank tests, two-sided, and not adjusted for multiple comparisons.

**Fig. 5:**
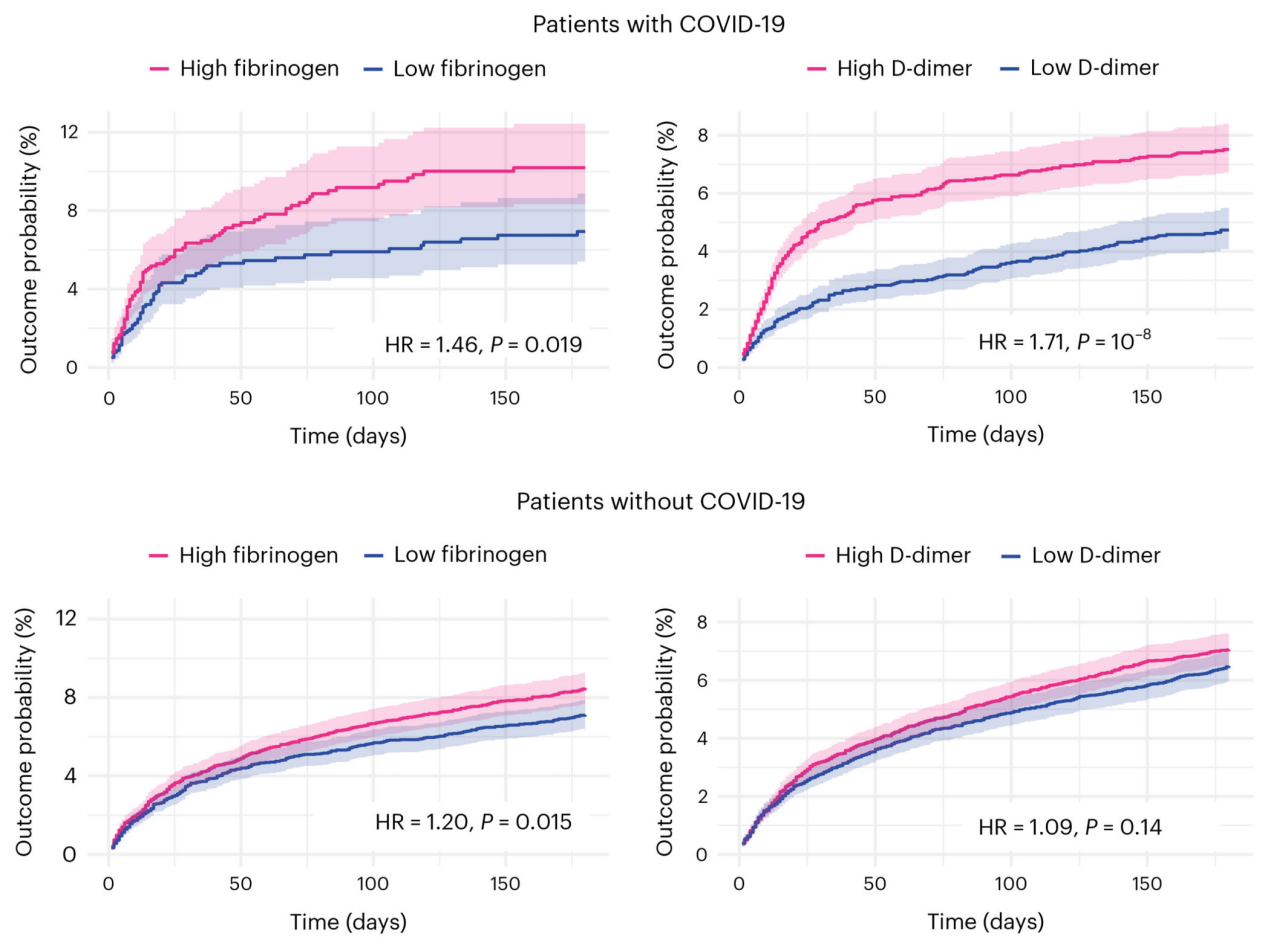
Replication and generalization of the findings using electronic health records data. Kaplan–Meier curves represent the cumulative incidence of cognitive deficits between those with high versus low fibrinogen (or D-dimer) and CRP level ≤10 mg l^-1^. The same analysis conducted in people without COVID-19 (bottom). Curves represent the Kaplan–Meier estimates and shading around curves represents 95% CI. *P* values are derived from log-rank tests, are two-sided and not adjusted for multiple comparisons. The same figures within the COVID-19 cohort wherein the criterion on CRP is relaxed to include all those with a CRP ≤ 20 mg l^-1^ or is removed altogether are presented as Extended Data Fig. 5.

To seek to replicate the first dimension of covariation, we compared those with acutely high fibrinogen (≥5.88 g l^-1^, taken to be the median of the population before matching) versus acutely low fibrinogen (<5.88 g l^-1^) and normal CRP (*n* = 1,276 in each cohort after matching; Supplementary Table 7 describes baseline characteristics). Acutely raised fibrinogen level was found to be significantly associated with post-COVID cognitive deficits (10.19% versus 6.94% incidence at 6 months in the high- versus low-fibrinogen cohorts; hazard ratio (HR) 1.46, 95% CI 1.06–2.02, *P* = 0.019; Fig. 5). Similar results were obtained when the maximum CRP level was doubled, but not when no limit was set on CRP (Extended Data Fig. 5).

To seek to replicate the second dimension of covariation, we compared those with acutely high D-dimer (≥14,700 μg l^-1^ (FEU), taken to be the median of the population before matching) versus acutely low D-dimer (<14,700 μg l^-1^ (FEU)) and normal CRP (*n* = 5,722 in each cohort after matching; Supplementary Table 8 shows baseline characteristics). D-dimer level was found to be significantly associated with post-COVID cognitive deficits (7.51% versus 4.74% incidence at 6 months in the high versus low D-dimer cohorts; HR 1.71, 95% CI 1.42–2.07, *P* < 0.0001; Fig. 5). Similar results were obtained when the maximum CRP level was doubled (in line with the primary findings) and when no limit was set on CRP, unlike the primary findings (Extended Data Fig. 5).

### Biomarker and cognitive profiles in the absence of COVID-19

To assess whether the associations between biomarkers and post-acute cognitive deficits can occur in other illnesses, we repeated the analyses based on EHR data described above in a pre-pandemic cohort of individuals (without COVID-19).

The association between high versus low fibrinogen and post-acute cognitive deficit was replicated among individuals without COVID-19 (*n* = 6,782 in each cohort after matching; HR 1.20, 95% CI 1.04–1.39, *P* = 0.015, Fig. 5; Supplementary Table 9 shows baseline characteristics) and was not significantly moderated by COVID-19 status when the risks were compared to those seen in people with COVID-19 (interaction HR 1.23, 95% CI 0.87–1.75, *P* = 0.25).

**Extended Data Fig. 6:**
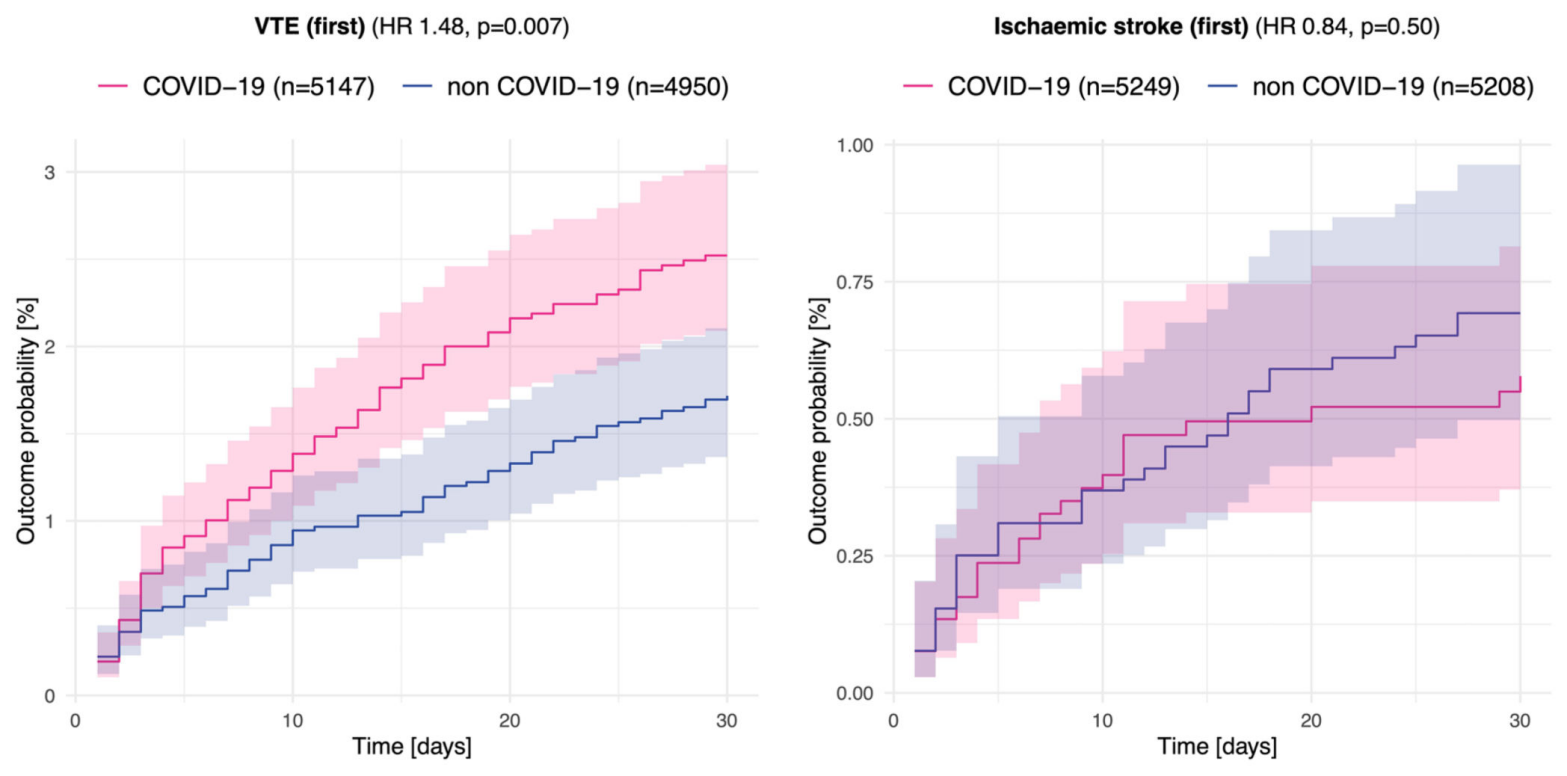
Associations between COVID-19 status and risks of venous thromboembolism and ischemic stroke among those with raised D-dimer. Comparison between matched cohorts of patients with high D-dimer and normal CRP with COVID-19 vs. without COVID-19 in terms of risk of venous thromboembolism [VTE] (left) and ischaemic stroke (right). Curves represent the Kaplan–Meier estimates and shading around curves represents 95% confidence intervals. P-values are derived from log-rank tests, two-sided, and not adjusted for multiple comparisons.

In contrast, the association between high versus low D-dimer and post-acute cognitive deficits was not significant in individuals without COVID-19 (*n* = 11,129 in each cohort after matching; HR 1.09, 95% CI 0.97–1.23, *P* = 0.14, Fig. 5; Supplementary Table 10 shows baseline characteristics) and there was significant moderation of this association by COVID-19 status (interaction HR 1.57, 95% CI 1.26–1.96, *P* < 0.0001). We further explored this moderation by COVID-19 status in a post-hoc analysis; individuals with COVID-19 and raised D-dimer were found to be at a higher risk of venous thromboembolism (VTE) at 30 d (HR 1.48, 95% CI 1.11–1.98, *P* = 0.007) but not ischemic stroke (HR 0.84, 95% CI 0.50–1.39, *P* = 0.50) compared to a matched cohort of individuals with raised D-dimer but without COVID-19 (Extended Data Fig. 6).

In other words, individuals with high fibrinogen are at an increased risk of post-acute cognitive deficits whether they had COVID-19 or not. In contrast, high D-dimer is only associated with post-acute cognitive deficits in those who had COVID-19, who differed from other people with high D-dimer in their risk of peripheral (VTE) rather than central (ischemic stroke) thrombosis.

## Discussion

This prospective cohort study of 1,837 patients hospitalized for COVID-19, augmented with a separate retrospective cohort study of EHR data, revealed two distinct dimensions linking acute blood biomarkers and post-acute cognitive deficits. A first dimension links high fibrinogen (relative to CRP) to objective and subjective cognitive deficits 6 and 12 months after infection. A second dimension links high D-dimer (relative to CRP) to subjective cognitive deficits, as well as occupational impact at 6 and 12 months after infection. The latter association was partially mediated by shortness of breath and fatigue at 6 months. These two dimensions were robust across secondary analyses and were broadly replicated in the separate large-scale EHR analysis, which also showed that the association with D-dimer is specific to COVID-19, unlike the association with fibrinogen.

In contrast to univariate regressions, in which covariations between a single biomarker and a cognitive outcome are estimated, CCA can capture more complex associations between biomarker and cognitive profiles. In particular, univariate regressions between fibrinogen (or D-dimer) and cognitive outcomes do not reveal significant associations as they fail to capture the important role of CRP in each biomarker profile (Supplementary Note 1). Unlike clusters, dimensions of covariation are not mutually exclusive so that individuals can score high on multiple dimensions. For instance, someone with high fibrinogen and high D-dimer relative to CRP would tend to score high on both dimensions and would be at higher risk of objective and subjective cognitive deficits and occupational impact.

Besides their statistical significance and robustness, results were also clinically meaningful. Individuals in the top half of the cohort along the first dimension had a mean C-PSQ at 6 months of 2.52 (out of 7) compared to 1.79 for those in the bottom half. This difference could occur, for instance, between individuals reporting some versus a lot of difficulties in both remembering/concentrating and understanding/being understood. Similarly, individuals in the top half of the cohort along the second dimension had a mean C-PSQ at 6 months of 2.90, reporting 40% of symptoms of subjective cognitive deficits on average (versus 1.42 or 20% for those in the bottom half). Being in the top half of the population along the second dimension was associated with a 6.8% absolute risk increase (22.1% versus 15.3%) of changing occupation and an 18.5% absolute risk increase of reporting difficulty working (60.2% versus 41.7%). Differences in MoCA scores along the first dimension were significant but more modest; this might in part reflect the lack of sensitivity of the MoCA to detect post-COVID brain fog (as opposed to mild cognitive impairment for which it has been validated^33^).

The associations were specific to biomarkers measured during the acute (rather than post-acute) phase and they cannot be accounted for simply by more severe illness (as there was no mediation by severity of the acute illness) nor by pre-existing cognitive deficits. Various mechanisms might explain how raised fibrinogen during the acute phase of COVID-19 can be associated with subsequent cognitive deficits. Fibrinogen is both a marker of inflammation (as an acute phase protein^34^) and hypercoagulable states^35^. It has a central role in coagulation with higher fibrinogen levels leading to faster fibrin formation and higher fibrin density, strength and stability^36^. COVID-19 is known to induce a hyper-coagulable state and to be associated with raised fibrinogen^37,38^. It is also thought that fibrinogen may directly affect the brain due to its unique structure containing binding sites for several receptors expressed in the nervous system, which might lead to microglial activation, axonal damage and binding of amyloid-β^39^. Raised fibrinogen level without raised CRP has been associated with cognitive deficit^40^ and subsequent dementia^41^. Fibrinogen can only reach the brain parenchyma if the blood–brain barrier is compromised, which can be caused by the SARS-CoV-2 main protease (M_pro_) inducing the death of brain endothelial cells^17^ or by fibrinogen itself via direct actions on these cells^39^(which would be compatible with the replication of findings among patients with raised fibrinogen but without COVID-19). Raised fibrinogen was only associated with post-acute cognitive deficits when raised relative to CRP. This might support the hypothesis that this biomarker profile results from hypercoagulopathy rather than an acute phase response. Another possibility is that raised fibrinogen relative to CRP represents delayed presentation to hospital with respect to infection onset, as fibrinogen remains elevated after CRP has peaked^34^. Delayed presentation might have deleterious health consequences that predispose to cognitive deficits. To distinguish these possibilities, studies with repeated biomarker measurements during the acute phase of COVID-19 would be informative. Taken together, our findings regarding the first dimension of covariation might reflect a combination of hypercoagulable state and the direct effects of fibrinogen on the brain.

Elevated D-dimer level is common during hospitalization with COVID-19 (refs. 35,42). It can have different causes^43^, but levels well above the normal limit (as observed for individuals in the top half of the cohort along the second dimension) often indicate the presence of thrombi^44^. The link between raised D-dimer and cognitive deficits might therefore reflect the presence of microthrombi in the cerebral vasculature, which have been observed in autopsies post-COVID-19 (ref. 22) and which tend to present with raised D-dimer and only moderately raised CRP^45^. But it might also reflect thromboembolism in the pulmonary vasculature. This is supported by the mediation of the association by shortness of breath and the observation in the EHR data that raised D-dimer associated with COVID-19 differs from raised D-dimer in the absence of COVID-19 in terms of risk of venous thromboembolism and post-acute cognitive deficits but not ischemic stroke. In addition, raised D-dimer correlates with reduced pulmonary perfusion in patients hospitalized with COVID-19 (ref. 46) and venous thromboembolism is thought to be associated with raised D-dimer but normal fibrinogen^47^, which would explain why this mechanism is captured by a separate dimension of covariation. It is plausible that COVID-19-induced pulmonary embolisms (PEs) lead to cerebral hypoxia which in turn leads to a subtle degree of cognitive impairment, which is subjectively evident but not easily objectively measured (as subjective cognitive deficit can be a sensitive sign of early decline)^48^. A separate explanation for the link between PE and cognitive deficit is that PE can lead to fatigue^49^, and fatigue can lead to subjective cognitive impairment in the absence of objective signs of deficit^48^. This is supported by the mediation by fatigue of the association between D-dimer and cognitive deficits. In summary, the association between raised D-dimer and subjective cognitive deficits might result from COVID-19-associated coagulopathy causing brain microthrombi or PEs with associated hypoxia or fatigue. The fact that subjective post-COVID cognitive deficits are associated with blood biomarkers might be validating for some patients reporting brain fog^26^ and highlights the importance for clinicians to avoid inferring that subjective deficits in the absence of objective signs are insignificant and cannot have a biological underpinning.

These mechanistic insights might help suggest further studies and treatment evaluations. For instance, investigations of brain imaging in people with post-COVID cognitive deficits might identify whether there is evidence of cerebral ischemia. If this is so, then evaluation of anticoagulants during the acute illness in a population at risk might be worthwhile. To further test whether post-COVID cognitive deficits can result from impaired pulmonary function, lung imaging combined with longitudinal cognitive and pulmonary function tests would be informative. If this proves to be a contributing mechanism, then adequate oxygen support, respiratory physiotherapy and/or enhanced prophylaxis for venous thromboembolism might be considered for clinical evaluation. If, in addition, fatigue is confirmed to be an important mediator, then adequate support with occupational and physiotherapy could also be considered.

As well as the mechanistic insights they provide, the results from this study might help in the development of predictive models of patients at risk of post-COVID cognitive deficits. Such predictive models are important to inform prognosis, recruit participants into studies aimed at testing prophylactic interventions and stratify interventions once they become available; however, the present study has not established the predictive value of biomarker profiles. That would require a different analytical approach, replication of the findings in a more heterogeneous population, integration with other predictors and the derivation of a validated predictive rule.

This study has strengths, including its longitudinal nature, large sample size, assessment of both subjective and objective cognitive function, several robustness analyses and replication (and extension) of findings using a large EHR database; however, it also has limitations. First, the cohort was recruited early in the pandemic before the emergence of many variants. This is partially mitigated by the replication using EHR data (not restricted to a specific variant). Second, participants in the prospective cohort study were all unvaccinated. Third, the study was observational and causal inference should not be drawn. While both the prospective cohort study and the retrospective EHR-based analyses were well adjusted for a range of covariates, residual confounding cannot be excluded. Fourth, cohorts were limited to hospitalized patients and findings might not generalize to people who did not require hospitalization but might still be at risk of cognitive deficits^2^. Fifth, we used a pragmatic approach to define subjective cognitive impairment based on data available within the PHOSP-COVID study (including seven self-rated items; Methods) rather than a validated scale. Sixth, this study cannot differentiate cognitive deficits that persisted since the acute illness from cognitive deficits that emerged after an initial recovery. While both timelines would qualify as a long COVID presentation^50^, they might have different pathogeneses, which this study cannot differentiate. Seventh, the replication of the results within a large-scale EHR dataset has its own limitations (1) objective and subjective cognitive deficits could not be separately investigated; and (2) comparison of biomarker profiles could only be achieved by creating and comparing cohorts rather than assessing the whole spectrum of values.

In summary, this prospective cohort study found two distinct dimensions linking acute blood biomarker profiles to post-acute cognitive profiles in patients hospitalized with COVID-19. A first dimension links raised fibrinogen relative to CRP with both objective and subjective cognitive deficits and might reflect immunothrombotic events with potential direct effects of fibrinogen on the brain. A second dimension links raised D-dimer relative to CRP with subjective but not objective cognitive deficits and with evidence of occupational impact. This dimension might reflect COVID-19-associated coagulopathy with thrombi in the cerebral or pulmonary vasculature. Mechanisms are speculative and further studies are needed to better delineate them. In the meantime, these biomarker profiles, based on routine blood tests, might help in the development of predictive models of post-COVID cognitive deficits, which could facilitate prognosis and accelerate research into management strategies.

## Online content

Any methods, additional references, Nature Portfolio reporting summaries, source data, extended data, supplementary information, acknowledgements, peer review information; details of author contributions and competing interests; and statements of data and code availability are available at https://doi.org/10.1038/s41591-023-02525-y.

## Methods

### PHOSP-COVID study

For our primary analysis, we used data from the Post-hospitalization COVID-19 study (PHOSP-COVID), which is a large-scale long-term study of 6,134 adults (aged ≥18 years) discharged from a hospital from one of 83 National Health Service (NHS) trusts in the UK with a clinical diagnosis of COVID-19 (between 29 January 2020 and 20 November 2021)^5,30^. For our analysis, we restricted the dataset to ‘Tier 2’ participants (*n* = 2,542) who had undergone additional specific research visits alongside routine clinical care. Tier 2 involved data collection at three time points: baseline (during hospitalization), at 2–7 months post-discharge (which corresponded to an average of about 6 months after admission and which we refer to as the 6-month follow-up for simplicity) and 12 months after hospital discharge (for a subset of participants). Collected measurements included routine clinical data on admission, results of blood tests on admission and at follow-up, as well as lifestyle, demographics and clinical scales. Patient demographics and characteristics oftheir acute COVID-19 admission, including confirmation of their COVID-19 diagnosis, treatments and organ support received, were obtained from hospital notes by the study team at each site. In this study, we focused on people who had a blood test recorded in hospital and completed a MoCA at 6 months so that the latter was available for each participant in our analysis.

More details about the study can be found in other papers^5,16,30^ and relevant variables are described below. Written informed consent was obtained from all study participants. The study was approved by the Leeds West Research Ethics Committee (20/YH/0225) and is registered on the ISRCTN Registry (ISRCTN10980107).

### Biomarker profiles

A blood sample was collected in participants during admission to hospital. When multiple blood samples were drawn, the first one upon admission was used. From that sample, the following six laboratory measurements were extracted: CRP, D-dimer (converted to FEU units if in D-dimer units), fibrinogen, lymphocytes, neutrophils and platelets. While the focus of this study was on biomarkers measured on admission to hospital, the same laboratory measurements were also acquired at the 6-month follow-up and their association with cognitive profiles was assessed in a post-hoc analysis. Supplementary Note 2 provides a description of the quality control of biomarker profiles.

### Cognitive profiles

At the 6-month visit and (for a subset of participants) at the 12-month visit, both clinician-acquired and patient-reported clinical scales were measured in participants. This study focuses on cognitive measurements at follow-up along two dimensions:

- The MoCA, which objectively measures cognitive deficits along seven domains: visuospatial and executive function, naming, attention, language, abstraction, delayed recall and orientation. Scores across domains are added and the maximum total score is 30 with a cutoff of 26 often used as a screening tool for dementia^51^.
- The C-PSQ, which assesses subjective cognitive deficits based on self-reported impairment in seven domains: confusion, short term memory loss, difficulty communicating, difficulty understanding or being understood, difficulty concentrating, slowing down of thinking and difficulty remembering (Supplementary Note 3 contains the definition ofeach item)^5^.

For both the objective and subjective cognitive deficits scales, scores for individual domains were used as input to the CCA (see below).

### Occupational impact

Occupational impact was captured in a subset of participants using two variables measured at 6 and 12 months. The first variable was the answer to a simple question ‘Has your illness affected your ability to do your usual work?’ and we refer to those answering positively to this question as having ‘difficulty working’. The second variable captures changes in occupation and was based on participants reporting that their occupation had changed between before and after their COVID-19 illness. A subset of participants also reported their occupation before and after COVID-19. We only recorded a positive outcome in those who reported a change in occupation and for whom the occupation after COVID-19 was not ‘Working full-time’ (as a change in occupation can also reflect an increase in number of hours worked). Similarly, for participants who reported a change in occupation and for whom there was no information on their occupation before and after their COVID-19 illness, we reported the change in occupation as ‘unknown’.

### Covariates

The following diagnoses (made before the diagnosis of COVID-19) and sociodemographic factors were included as covariates in the analysis:

- Respiratory condition
- Rheumatological condition
- Cardiovascular condition
- Gastrointestinal condition
- Cerebrovascular accident
- Dementia
- Parkinson’s disease
- Psychiatric or neurological condition, captured by a participant answering ‘Yes’ to any of the following: (1) depression or anxiety; (2) treatment with an antidepressant; (3) treatment by a mental health professional; or (iv) other chronic neurological disorder
- CFS, fibromyalgia or chronic pain
- Diabetes mellitus
- Hypothyroidism/hyperthyroidism or other chronic metabolic/ endocrine disorder
- Chronic kidney disease
- Cancer
- Chronic infectious diseases
- Educational level (highest level completed) encoded as a categorical variable with the following eight categories: (1) none; (2) primary school; (3) secondary school (GCSE level, NVQ level 1/2 or equivalent); (4) sixth-form college (A-levels, NVQ level 3 or equivalent); (5) vocational qualification (NVQ level 4 or equivalent); (6) undergraduate university degree or NVQ level 5 or equivalent; (7) postgraduate qualification; and (8) prefer not to say
- Annual household income encoded as a categorical variable with the following categories: (1) <£19,000; (2) £19,001–26,000; (3) £26,001–35,000; (4) £35,001–48,000; (5) >£48,001; and (6) prefer not to say
- Marital status encoded with a single binary variable (married versus not)
- Whether English was a participant’s first language, as reported by the patient and encoded as a binary variable
- Sex
- Ethnicity

### Canonical correlation analysis

CCA is a method used to find linear relationships between two separate sets ofvariables which are both measured in the same individuals. In our case, CCA was used to find relationships between blood biomarkers on admission to hospital (six variables measured in each individual) and 14 individual items of the cognitive assessments at the 6-month follow-up (seven items from the MoCA domains and seven items from the C-PSQ domains). Each of the six biomarker and each of the 14 cognitive scores were first adjusted for each covariate defined above using a generalized linear model and the *z* score-standardized adjusted biomarkers and cognitive scores were input to the CCA. The outputs of CCA were pairs of linear combinations: one linear combination of blood biomarkers (a weighted sum of blood test results) and one linear combination of cognitive scores (a weighted sum of cognitive items) so that the former was maximally correlated with the latter. A linear combination of biomarkers summarizes all blood test results by a single number: individuals with a particular combination of blood test results will score high on that number, whereas others will score low and in this sense, we refer to that linear combination as a ‘biomarker profile’. The same applies to the weighted sum of cognitive items and the resulting ‘cognitive profile’.

Because the biomarker and cognitive profiles are maximally correlated, individuals can be represented along a single dimension, which links the two (the line of best fit between the biomarker and cognitive profiles). We refer to this dimension as a dimension of covariation (sometimes also referred to as a mode of covariation). The location of individuals along that dimension can be calculated as the mean of their biomarker and cognitive profiles.

Once a dimension has been discovered and found to be statistically significant (see next section), its correlation with other variables not used as input to CCA can be calculated to provide further insight into the covariation it captures. This can be a correlation with the biomarker profile, the cognitive profile or the mean profile (the location of an individual along the dimension) depending on the association of interest. In this study, we calculated the Pearson’s correlation coefficient between each cognitive profile and the total MoCA score and C-PSQ score at 6 months (adjusted for all covariates described above) to better understand what the cognitive profile represented (as it was made of individual MoCA and C-PSQ items rather than total scores). We also calculated correlation between the cognitive profiles at 6 months and the total MoCA and C-PSQ scores at 12 months to assess whether the association was longer-lasting than 6 months. Cognitive items at the 12-month follow-up were not included as input to CCA to provide an opportunity to test whether the association discovered using data measured at 6-month follow-up can predict outcomes at later time points and because they were not available for all individuals. Similarly, we calculated correlation with occupational outcomes (ability to work and occupational changes) at 6 and 12 months to provide insight into the possible association between dimensions of covariation and occupational impact.

Finally, we also assessed whether the dimensions were significantly associated with predefined recovery clusters (as defined in a previous analysis based on a subset of 767 participants of the PHOSP-COVID study^5^ and here, applied to all participants) based on patient symptom questionnaires, physical performance and cognitive assessment data. The four resulting clusters, stratifying patients in terms of the severity of their recovery and the level of subsequent impairment, were categorized as follows:

- Mild impairment
- Moderate impairment with cognitive impairment
- Severe impairment
- Very severe impairment

Recovery cluster variable was encoded categorically with mild impairment used as a reference level.

### Statistical analysis

Blood biomarker values were transformed to a log scale when the log-transformed variable was found to be more normally distributed than the linear-scale variable as determined by a Shapiro–Wilk normality test. This implied that D-dimer, neutrophils, platelets, CRP and lymphocytes were all log-transformed. All input variables to the CCA were first adjusted for all covariates specified above using generalized linear models in which the CCA inputs (blood biomarker or cognitive item) were the dependent variables and the covariates were independent variables. Logistic regressions were used for binary variables (for example all yes/no answers to C-PSQ items) and linear regressions otherwise. Missing data (in terms of adjusted biomarker values or cognitive items) were imputed using multiple imputation by chain equation model with 20 chains and five iterations, using the mice package in R (v.3.14.0). Imputed data were then used as input to CCA and Rubin rule was used to combine them^52^. This approach to imputation was used under the assumption of missingness at random (that is that conditional on covariates and biomarker values, missing data were randomly distributed across participants). This assumption is justified given the large number of covariates and the fact that blood samples were collected for all individuals included in this study, so that missing biomarker values represent small departure from the protocol (for example a clinician forgetting to request part of laboratory investigations) rather than a participant not having a blood test at all. The number of imputations (number of chains and iterations) was justified by examination of convergence plots and by repeating the whole analysis (including multiple imputations and CCA analysis) three times and checking for stability of the results across the three repetitions.

To assess whether the dimensions of covariation were statistically significant, permutation tests with 10,000 permutations were applied. Within each permutation, the subject IDs of the cognitive scores were randomly permuted relative to those of the biomarker scores, CCA was applied to the result and the maximum correlation coefficient achieved (in absolute value) was recorded. Comparison against this null distribution of maximum correlation coefficients therefore controls for multiple comparisons across dimensions of covariation. The *P* value for a dimension of covariation was calculated using the formula for permutation tests:

$$P=\frac{1+n_{>}}{1+n}$$

where *n* = 10,000 is the number ofpermutations and *n*_>_ is the number of permutations for which the correlation coefficient was greater (in absolute value) than that observed in the non-permuted dataset.

Similarly, to assess whether correlations between external variables (for example occupational outcomes or cognitive scores at 12 months) and dimensions of covariation were statistically significant, the subject IDs for the external variable were permuted 10,000 times and the correlation coefficients were calculated for each permutation. This leads to null distributions for each correlation coefficient of interest, from which a *P* value can be calculated using the above formula.

To better appreciate how different variables are distributed along dimensions of covariation, we divided the cohort into subgroups based on their location along that mode (those above and those below the median along the dimension) and we compared the values of different variables between the subgroups. Raw data are presented as single dots per individual for continuous variables and contingency tables for dichotomous variables and effect sizes are summarized as Cohen’s *d* for continuous variables and ORs for dichotomous variables. Only complete (not imputed) data are represented in this way to display with more transparency the available data (for example this clearly shows that MoCA at 12 months had fewer records than MoCA at 6 months). The association with recovery clusters was reported as 4 × 2 contingency tables (representing the distribution of individuals over the four clusters of severity and between the top and bottom half of the cohort along the dimension) and the null hypothesis that being in the top or bottom half of the cohort did not affect the odds of being severely impaired was tested using Fisher’s exact test.

To assess whether any other aspects of a person’s health in the post-acute phase of COVID-19 might mediate the association between biomarker and cognitive profiles, individual mediation analyses were conducted in which the biomarker profile was the independent variable, the cognitive profile was the dependent variable and the other aspects of individual health were mediators. These aspects were encoded by 14 clinical scales capturing ten domains of health, including shortness of breath (using the Dyspnea-12 scale), fatigue (FACIT fatigue scale), pain (BPI interference and severity scales), sarcopenia (SARC-F), cardiopulmonary exercise (ISWT as absolute score and % predicted), physical activity (GPPAQ occupational and exercise subscales and physical activity index), physical performance (SPPB), depression (PHQ-9), PTSD (PCL-5) and anxiety (GAD-7 scale). For each mediation analysis, the fraction of the association explained by the mediators (sometimes referred to as the ratio of the ‘indirect effect’ to the ‘total effect’) was tested against the null hypothesis that it equals zero using the mediation R package (v.4.5.0).

To assess whether the association between the biomarker and cognitive profiles can be entirely explained by severity of the acute illness, we conducted a single mediation analysis with multiple mediators representing different aspects of the acute illness severity, including:

- World Health Organization (WHO) clinical progression scale^53^, which is a scale defined by the WHO to capture the level of respiratory support needed by patients with COVID-19. It consists of four levels: no oxygen required (level 0); supplemental oxygen required (level 1); ventilation required (level 2, which we captured based on either continuous positive airway pressure ventilation, bi-level non-invasive ventilation or high-flow nasal oxygen needed at any point during hospital admission); and last, invasive ventilation/oxygenation required (level 3, which was captured as either invasive mechanical ventilation or extra-corporeal membrane oxygenation). This was encoded as a continuous variable.
- National Early Warning Scores (NEWS) on admission to hospital (first recorded NEWS from admission). This scale captures the degree of departure of physical observations from their normal range and is used nationally in the NHS in the UK. It is a score ranging from 0 to 20, which we encoded as a continuous variable. Specifically, the following scoring is applied for the different physical observations and the total score is obtained by summing up the scores for the different items:

- Respiratory rate (breaths per min): ≤8 (+2 points), 9–11 (+1 point), 12–20 (0 points), 21–21 (+2 points) and ≥25 (+3 points);
- Oxygen saturation: ≤91% (+3 points), 92–93% (+2 points), 94–95% (+1 point) and ≥96% (+0 points);
- Any supplemental oxygen: no (+0 points) and yes (+2 points);
- Temperature: ≤35 °C (+3 points), 35.1–36 °C (+1 point), 36.1–38 °C (+0 points), 38.1–39 °C (+1 point) and ≥39.1 °C (+2 points);
- Systolic blood pressure (mm Hg): ≤90 (+3 points), 91–100 (+2 points), 101–110 (+1 point), 111–219 (+0 points) and ≥220 (+3 points);
- Heart rate (beats per minute): ≤40 (+3 points), 41–50 (+1 point), 51–90 (+0 points), 91–110 (+1 point), 111–130 (+2 points) and ≥131 (+3 points).

- Duration of hospital admission: captured from the participant’s health record and recorded as a continuous variable.
- Admission to intensive care: captured from the participant’s health record and recorded as a dichotomous variable.
- Presence of altered consciousness or confusion during admission: captured from the participant’s health record and recorded as a dichotomous variable.

The residual ‘direct effect’ linking biomarker and cognitive profiles after accounting for the above mediators was tested against the null hypothesis that it is zero using the lavaan package (v.0.6.14) which uses a *z* statistic to compute a *P* value.

All statistical analyses were conducted in R v.4.2.0. Statistical significance was defined based on a two-tailed *P* < 0.05.

### Robustness analyses

The robustness of the results was tested in four ways. First, random split analysis was conducted in which the cohort was randomly split in two sub-cohorts of equal size (±1) and the analysis was repeated in each sub-cohort. Within each repetition, the coefficients defining the biomarker and cognitive profiles (the weights of the weighted sums defining those profiles) for the first two dimensions of covariation were compared to those in the primary analysis using Pearson’s correlation coefficient (one correlation coefficient for the biomarker profile and one for the cognitive profile). Because CCA is defined up to the sign of the profiles (multiplying both the biomarker and cognitive profiles by -1 would be an equivalent result from a CCA point of view), the sign was defined so that the maximum correlation coefficient (in absolute value) was positive. The 200 correlations thereby generated (100 repetitions × two sub-cohorts) were then averaged and reported. To assess whether these average correlation coefficients were statistically significant, a permutation test was used in which the whole process was repeated 1,000 times after permuting the subject IDs of the biomarker values with respect to the cognitive scores (within each permutation, 100 random splits of the data were generated and the average correlation was calculated). This process generated a null distribution of average correlation coefficients against which the initial average correlation coefficients could be compared to calculate a *P* value.

Second, leave-one-out cross-validation was performed. This was achieved by leaving one participant out and calculating CCA and dimensions of covariation using data from all the other participants. The biomarker and cognitive profiles defined based on all other participants were then calculated in the left-out individual. We repeated this process across all participants resulting in biomarker and cognitive profiles for each participant estimated using data from all the others. The correlation coefficients between the biomarker and cognitive profiles thereby estimated for the first two dimensions were calculated and the null hypothesis that it equals zero was tested using a *t*-test.

Third, data were limited to complete cases (those with missing data on any biomarker values or cognitive items, or covariates were excluded, *n* = 355) and the correlation between the biomarker and cognitive profiles for the first two dimensions of covariation was compared to that observed in the whole cohort with imputed data. The results were deemed robust in this complete case dataset if the correlations were both significantly greater than zero in this restricted sample and were not significantly different from the correlation coefficients in the whole sample.

Fourth, we assessed whether our findings could be attributed to pre-COVID cognitive deficits. A large subgroup of the PHOSP-COVID cohort (*n* = 547) was asked, at the 6-month follow-up, to report (retrospectively) what their cognitive function was before they had COVID-19 using a subset of items of the C-PSQ scale. Specifically, they were asked:

- Before you had COVID-19, did you have difficulty remembering or concentrating?
- Before you had COVID-19, did you have difficulty communicating, for example understanding or being understood? They could answer each of these two questions by choosing from the following options:
  1. No: 0 points
  2. Yes, some difficulty: + 1 point
  3. Yes, a lot of difficulty: + 2 points
  4. Yes, could not do at all: +3 points

As part of the C-PSQ and during the same follow-up visit, they were also asked to answer the following two questions which assessed their current cognitive function (and which they could also answer by choosing from the four options above):

C. Currently, do you have difficulty remembering or concentrating?
D. Currently, do you have difficulty communicating, for example understanding or being understood?

Because C and D are two items from the C-PSQ (Supplementary Note 3), we refer to the sum of their scores as C-PSQ-2 at 6 months and the sum of the scores of answers to questions A and B as the pre-COVID C-PSQ-2. Questions C and D were then repeated at 12 months in 205 participants providing a C-PSQ-2 at 12 months.

This longitudinal dataset containing both pre- and post-COVID cognitive scores allowed us to assess whether pre-COVID cognitive deficits could explain the associations observed in this study. We first assessed whether cognitive deficits at 6 and 12 months merely reflected pre-existing cognitive deficits by testing whether there were significant changes in C-PSQ-2 between before and after COVID-19. We then assessed whether pre-existing cognitive deficits predicted biomarker profiles, which would indicate that they might confound the association between biomarker and post-acute cognitive profiles. Finally, we assessed whether C-PSQ-2 at 6 and 12 months was associated with dimensions of covariation (which is important as there is no guarantee that limiting C-PSQ to two items encodes the kinds of subjective cognitive deficits captured by the two dimensions of covariation) and, ifso, whether changes in C-PSQ-2 between pre-COVID and 6 and 12 months post-COVID were also associated with these dimensions.

### Replication and expansion with EHR data

We sought to replicate the findings from the prospective PHOSP-COVID study using a retrospective cohort study based on EHR data.

### Study design and data collection

We used data from the TriNetX Analytics Network, a large-scale federated EHR network which, at the time of study, holds anonymized data from over 90 million patients within 57 healthcare organizations, primarily in the US. Patient information collected on the platform includes demographics, diagnoses (encoded as ICD-10 codes), medications and procedures. Using the TriNetX platform, cohorts can be created on the basis of inclusion and exclusion criteria, matched for confounding variables with a built-in propensity-score-matching algorithm and compared for outcomes of interest over specified time periods.

### Cohorts

Two cohorts were compared to seek to reproduce each of the first and second dimensions of covariation, based on the following inclusion/exclusion criteria.

Cohort 1 was defined as all patients meeting the following criteria:

- (A) The individual was hospitalized with COVID-19 (ICD-10 code U07.1) on or after 20 January 2020 (date of first case of COVID-19 in the United States).
- (B_1_) The individual had a recorded fibrinogen level >5.88 g l^-1^ (which was the median value in the cohort defined by criterion A) between 4 d before and 2 weeks after their hospital admission with COVID-19. The reason for including those with a fibrinogen level within 4 d before their COVID-19 diagnosis is that 4 d was considered to be the maximum time taken for a SARS-CoV-2 test result to become available.
- (C) The individual had a recorded CRP level ≤10 mgl^-1^. The reason for including this criterion is that the first dimension of covariation was found to be such that raised fibrinogen was not accompanied by correspondingly raised CRP (despite the correlation between the two at the cohort level). As discussed in the Results, this is akin to adjusting for CRP level. Adjusting for post-exposure variables (such as CRP) within TriNetX is only possible by restricting the cohorts to have the value within a specific range.
- (D) The individual was still alive at the time of the analysis. Cohorts 2, 3 and 4 were defined as meeting criteria A, C and D as above, but with criterion B_1_ replaced by B_2_, B_3_ and B_4_, respectively:
- (B_2_) The individual had a recorded fibrinogen level ≤5.88 g l^-1^ between 4 d before and 2 weeks after their hospital admission with COVID-19. They could not have had a fibrinogen level >5.88 g l^-1^ within that time window to avoid including those from cohort 1 who had a normalized fibrinogen level during this time window.
- (B_3_) The individual had a recorded D-dimer level >14,700 μg l^-1^ (FEU) (which was the median value in the cohort defined by criterion A) between 4 d before and 2 weeks after their hospital admission with COVID-19.
- (B_4_) The individual had a recorded D-dimer level ≤14,700 μg l^-1^ (FEU) between 4 d before and 2 weeks after their hospital admission with COVID-19. They could not have had a D-dimer level >14,700 μg l^-1^ (FEU) within that time window to avoid including those from cohort 3 who had a normalized D-dimer level during this time window.

To seek to replicate the first dimension of covariation, cohort 1 was matched to and then compared to cohort 2. To seek to replicate the second dimension of covariation, cohort 3 was matched to and then compared to cohort 4 (see below for details). To explore the importance of criterion C in the definitions of cohorts above, the analyses were repeated by increasing the limit on CRP to any level ≤20 mg l^-1^ and by removing the criterion altogether.

Finally, to assess whether the same association between biomarker and cognitive profiles could be observed in the absence of COVID-19, an additional set of four cohorts were defined exactly as cohorts 1–4 but with criterion A modified by A′:

(A′). The individual was hospitalized on or before 24 July 2019. The latter date corresponds to 6 months (180 d) before the first case of COVID-19 in the United States, so that all these individuals did not have COVID-19 at the time of their biomarker measurements nor during the 6-month follow-up that ensued.

This resulted in cohorts 1′–4′; cohort 1′ was matched to and compared to cohort 2′ and cohort 3′ was matched to and compared to cohort 4′.

### Outcomes

We used a time-to-event analysis with a 180-d follow-up. The primary outcome was a composite of ICD-10 codes capturing the range of diagnostic codes that patients presenting with ‘brain fog’ might receive, as defined in our previous studies^2,3,31,54^. Specifically the following codes were used: F05 (‘Delirium due to known physiological condition’), F06.8 (‘Other specified mental disorders due to known physiological condition’), G93.40 (‘Encephalopathy, unspecified’), R40 (‘Somnolence, stupor and coma’), R41 (‘Other symptoms and signs involving cognitive functions and awareness’) or R48 (‘Dyslexia and other symbolic dysfunction’), F01 (‘Vascular dementia’), F02 (‘Dementia in other disease classified elsewhere’), F03 (‘Unspecified dementia’), G30 (‘Alzheimer’s disease’), G31.0 (‘Frontotemporal dementia’), G31.83 (‘Dementia with Lewy bodies’) and G31.84 (‘Mild cognitive impairment’ (MCI)).

In a post-hoc analysis, we explored possible reasons for the significant moderation by COVID-19 status of the association between D-dimer and post-acute cognitive deficits by propensity-score-matching cohort 3 to cohort 3′ and comparing the risk of a first ischemic stroke (ICD-10 code I63) and a first VTE (ICD-10 code I82) within the first 30 d since biomarker measurement.

### Statistical analysis

In each comparison, the two cohorts being compared were propensity-score-matched on covariates which are confirmed or suspected risk factors for COVID-19, more severe COVID-19 illness or subsequent neuropsychiatric consequences of COVID-19, including^2,3,32,55–57^ age, sex, ethnicity, race, socioeconomic deprivation, obesity, diabetes, hypertension, ischemic heart disease and other forms of heart disease, asthma, chronic lower respiratory diseases, chronic kidney disease, organ transplant, nicotine dependence, other substance use disorder, neoplasm (both benign and malignant), hematological cancer, chronic liver disease, stroke, dementia, rheumatoid arthritis, lupus, psoriasis, disorders involving an immune mechanism, psychotic disorders, mood disorders, anxiety disorders, insomnia, somnolence, delirium, brain hemorrhage, Parkinson’s disease, Guillain–Barré syndrome, nerve, nerve root or plexus disorders, diseases ofmyoneuraljunction and muscle, encephalitis, encephalopathy, dyslexia and other symbolic dysfunctions, MCI, epilepsy, convulsions, COVID-19 vaccine, antidepressants (with fluvoxamine in particular), antipsychotics (with clozapine in particular) and lithium. More details on covariates including ICD-10 codes, can be found in Supplementary Note 4.

Matching (1:1) was achieved using a greedy nearest neighbor algorithm with caliper distance of 0.1. For each characteristic, matching was considered to be successful where the standardized mean difference between the cohorts was <0.1 (ref. 58). The propensity score was calculated using a logistic regression (implemented by the function LogisticRegression of the scikit-learn package in Python 3.7), including each of the covariates mentioned above. To eliminate the influence of ordering of records, the order of the records in the covariate matrix was randomized before matching.

The Kaplan–Meier estimator was used to estimate the incidence of each outcome and the log-rank test to test for differences between cohorts. HRs with 95% CI were calculated using a Cox proportional hazards model.

## Extended Data

## Supplementary Material

Supplementary Material

## Figures and Tables

**Table 1:** Baseline characteristics for the whole cohort and the sub-cohorts that score in the top and bottom half along the first and second dimensions of covariation discovered in this study

Cohort	Whole cohort	Bottom half on first dimension	Top half on first dimension	Bottom half on second dimension	Top half on second dimension
Number	1,837	919	918	919	918
Demographics					
Age, mean (s.d.)	57.9 (12.4)	58.0 (12.7)	57.8 (12.2)	57.8 (12.6)	58.0 (12.3)
Sex, *n* (%)					
Female	673 (36.6)	330 (35.9)	343 (37.4)	316 (34.4)	357 (38.9)
Male	1,060 (57.7)	540 (58.8)	520 (56.6)	553 (60.2)	507 (55.2)
Missing	104 (5.7)	49 (5.3)	55 (6.0)	50 (5.4)	54 (5.9)
Race, *n* (%)					
White	1,385 (75.4)	689 (75.0)	696 (75.8)	689 (75.0)	696 (75.8)
Mixed	26 (1.4)	16 (1.7)	10 (1.1)	14 (1.5)	12 (1.3)
Asian	217 (11.8)	103 (11.2)	114 (12.4)	117 (12.7)	100 (10.9)
Black	104 (5.7)	52 (5.7)	52 (5.7)	54 (5.9)	50 (5.4)
Other	58 (3.2)	31 (3.4)	27 (2.9)	30 (3.3)	28 (3.1)
Unknown	47 (2.6)	28 (3.0)	19 (2.1)	15 (1.6)	32 (3.5)
Education					
Primary school	40 (2.2)	18 (2.0)	22 (2.4)	17 (1.8)	23 (2.5)
Secondary school	550 (29.9)	274 (29.8)	276 (30.1)	281 (30.6)	269 (29.3)
Sixth-form college	237 (12.9)	117 (12.7)	120 (13.1)	114 (12.4)	123 (13.4)
Vocational qualification	222 (12.1)	108 (11.8)	114 (12.4)	107 (11.6)	115 (12.5)
Undergraduate university degree	301 (16.4)	150 (16.3)	151 (16.4)	160 (17.4)	141 (15.4)
Postgraduate qualification	237 (12.9)	122 (13.3)	115 (12.5)	123 (13.4)	114 (12.4)
Prefer not to say	51 (2.8)	25 (2.7)	26 (2.8)	24 (2.6)	27 (2.9)
None	50 (2.7)	26 (2.8)	24 (2.6)	29 (3.2)	21 (2.3)
Missing	149 (8.1)	79 (8.6)	70 (7.6)	64 (7.0)	85 (9.3)
Income, *n* (%)					
<£19,000	252 (13.7)	116 (12.6)	136 (14.8)	117 (12.7)	135 (14.7)
£19,001–26,000	207 (11.3)	97 (10.6)	110 (12.0)	107 (11.6)	100 (10.9)
£26,001–35,000	202 (11.0)	100 (10.9)	102 (11.1)	104 (11.3)	98 (10.7)
£35,001–48,000	200 (10.9)	103 (11.2)	97 (10.6)	102 (11.1)	98 (10.7)
>£48,001	439 (23.9)	224 (24.4)	215 (23.4)	227 (24.7)	212 (23.1)
Prefer not to say	386 (21.0)	198 (21.5)	188 (20.5)	193 (21.0)	193 (21.0)
Missing	151 (8.2)	81 (8.8)	70 (7.6)	69 (7.5)	82 (8.9)
Is married, *n* (%)					
Yes	1,034 (56.3)	517 (56.3)	517 (56.3)	523 (56.9)	511 (55.7)
No	669 (36.4)	334 (36.3)	335 (36.5)	335 (36.5)	334 (36.4)
Missing	134 (7.3)	68 (7.4)	66 *(7.2)*	61 (6.6)	73 (8.0)
English as the first language, *n* (%)					
Yes	1,469 (80.0)	734 (79.9)	735 (80.1)	733 (79.8)	736 (80.2)
No	244 (13.3)	123 (13.4)	121 (13.2)	133 (14.5)	111 (12.1)
Missing	124 (6.8)	62 (6.7)	62 (6.8)	53 (5.8)	71 (7.7)
Comorbidities, *n* (%)					
Cardiovascular condition	826 (45.0)	410 (44.6)	416 (45.3)	400 (43.5)	426 (46.4)
History of cerebrovascular accident	79 (4.3)	37 (4.0)	42 (4.6)	35 (3.8)	44 (4.8)
Dementia	<10	<10	<10	<10	<10
Parkinson’s disease	<10	<10	<10	<10	<10
Psychiatric or neurological condition	332 (18.1)	168 (18.3)	164 (17.9)	159 (17.3)	173 (18.8)
ME/CFS/fibromyalgia/chronic pain	93 (5.1)	45 (4.9)	48 (5.2)	44 (4.8)	49 (5.3)
Diabetes	366 (19.9)	179 (19.5)	187 (20.4)	173 (18.8)	193 (21.0)
Respiratory condition	507 (27.6)	261 (28.4)	246 (26.8)	249 (27.1)	258 (28.1)
Rheumatological condition	285 (15.5)	148 (16.1)	137 (14.9)	136 (14.8)	149 (16.2)
Gastrointestinal condition	391 (21.3)	199 (21.7)	192 (20.9)	194 (21.1)	197 (21.5)
Endocrine condition	147 (8.0)	71 *(7.7)*	76 (8.3)	74 (8.1)	73 (8.0)
Chronic kidney disease	72 (3.9)	37 (4.0)	35 (3.8)	30 (3.3)	42 (4.6)
History of cancer	134 (7.3)	66 *(7.2)*	68 (7.4)	67 (7.3)	67 (7.3)
Chronic infectious disease	38 (2.1)	20 (2.2)	18 (2.0)	22 (2.4)	16 (1.7)
Follow-up					
Number at 6 months, *n* (%)	1,837 (100.0)	919 (100.0)	918 (100.0)	919 (100.0)	918 (100.0)
Time at 6 months, median (IQR), days	176 (135–206)	176 (137–206)	177 (133–206)	177 (134–207)	175 (138–205)
Number at 12 months, *n* (%)	626 (34.0)	308 (33.5)	318 (34.6)	310 (33.7)	316 (34.4)
Time at 12 months, median (IQR), days	403 (375–426)	404 (376–426)	402 (374–428)	406 (376–427)	400 (374–426)
Diagnosis of COVID-19					
Positive PCR test, *n* (%)	1,553 (84.5)	795 (86.5)	758 (82.6)	795 (86.5)	758 (82.6)
Undocumented method, *n* (%)	284 (15.5)	124 (13.5)	160 (17.4)	124 (13.5)	160 (17.4)

IQR, interquartile range; ME, myalgic encephalomyelitis.

## Data Availability

For the PHOSP-COVID data, the protocol, consent form, definition and derivation of clinical characteristics and outcomes, training materials, regulatory documents, requests for data access and other relevant study materials are available online at https://www.phosp.org. For the TriNetX data, the system returned the results of these analyses as csv files, which we downloaded and archived. Aggregate data, as presented in this article, can be freely accessed at https://osf.io/kzhfs/. This study had no special privileges. Inclusion criteria specified in Methods and Supplementary Information would allow other researchers to identify similar cohorts of patients as we used here for these analyses; however, TriNetX is a live platform with new data being added daily so exact counts will vary. To gain access to the data, a request can be made to TriNetX (join@trinetx.com), but costs might be incurred and a data-sharing agreement would be necessary.

## Reporting summary

Further information on research design is available in the Nature Portfolio Reporting Summary linked to this article.

## Code availability

The code to reproduce the analyses can be accessed via https://osf.io/kzhfs/.
