# Supplementary material for: Acute blood biomarker profiles predict cognitive deficits 6 and 12 months after COVID-19 hospitalization
Source: Nat Med. Author manuscript; Available in PMC 2023 Oct 18. (PMC10579097; doi:10.1038/s41591-023-02525-y)
Supplement: Supplementary Material [file EMS188593-supplement-Supplementary_Material.pdf]

nature medicine

Article

https://doi.org/10.1038/s41591-023-02525-y

Acute blood biomarker profiles predict cognitive deficits 6 and 12 months after COVID-19 hospitalization

In the format provided by the authors and unedited

Supplementary Material

PHOSP-COVID Collaborative Group

Core Management Group

Chief Investigator C E Brightling, *Members* R A Evans (Lead Co-I), L V Wain (Lead Co-I), J D Chalmers, V C Harris, L P Ho, A Horsley, M Marks, K Poinasamy, B Raman, A Shikotra, A Singapuri

PHOSP-COVID Study Central Coordinating Team

C E Brightling (Chief Investigator), R A Evans (*Lead Co-I*), L V Wain (*Lead Co-I*), R Dowling, C Edwardson, O Elneima, S Finney, N J Greening, B Hargadon, V C Harris, L Houchen--Wolloff, O C Leavy, H J C McAuley, C Overton, T Plekhanova, R M Saunders, M Sereno, A Singapuri, A Shikotra, C Taylor, S Terry, C Tong, B Zhao

Steering Committee

Co-chairs D Lomas, E Sapey*, Institution representatives* C Berry, C E Bolton, N Brunskill, E R Chilvers, R Djukanovic, Y Ellis, D Forton, N French, J George, N A Hanley, N Hart, L McGarvey, N Maskell, H McShane, M Parkes, D Peckham, P Pfeffer, A Sayer, A Sheikh, A A R Thompson, N Williams and core management group representation

Executive Board

Chair C E Brightling, representation from the core management group, each working group and platforms

Platforms

Bioresource

W Greenhalf (*Co-Lead*), M G Semple (*Co-Lead*), M Ashworth, H E Hardwick, L Lavelle-Langham, W Reynolds, M Sereno, R M Saunders, A Singapuri, V Shaw, A Shikotra, B Venson, L V Wain

Data Hub

A B Docherty (*Co-Lead*), E M Harrison (*Co-Lead*), A Sheikh (*Co-Lead*), J K Baillie, C E Brightling, L Daines, R Free, R A Evans, S Kerr, O C Leavy, N I Lone, H J C McAuley, R Pius, J Quint, M Richardson, M Sereno, M Thorpe, L V Wain

Imaging Alliance

M Halling-Brown (*Co-Lead*), F Gleeson (*Co-Lead*), J Jacob (*Co-Lead*), S Neubauer (*Co-Lead*) B Raman (*Co-Lead*) S Siddiqui (*Co-Lead*) J M Wild (*Co-Lead*), S Aslani, G Baxter, M Beggs, C Bloomfield, M P Cassar, A Chiribiri, E Cox, D J Cuthbertson, M Halling-Brown, V M Ferreira, L Finnigan, S Francis, P Jezzard, G J Kemp, H Lamlum, E Lukaschuk, C Manisty, G P McCann , C McCracken, K McGlynn , R Menke , C A Miller , A J Moss, T E Nichols, C Nikolaidou , C O'Brien , G Ogbole, B Rangelov, D P O'Regan , A Pakzad, S Piechnik , S Plein, I Propescu, A A Samat, L Saunders, Z B Sanders, R Steeds, T Treibel, E M Tunnicliffe, M Webster, J Willoughby, J Weir McCall, C Xie, M Xu

Omics

L V Wain (*Co-Lead)*, J K Baillie (*Co-Lead*), H Baxendale, C E Brightling, M Brown, J D Chalmers, R A Evans, B Gooptu, W Greenhalf, H E Hardwick, R G Jenkins, D Jones, I Koychev, C Langenberg, A Lawrie, P L Molyneaux, A Shikotra, J Pearl, M Ralser, N Sattar, R M Saunders, J T Scott, T Shaw, D Thomas, D Wilkinson

Working Groups

Airways

L G Heaney (*Co-Lead*), A De Soyza (*Co-Lead*), D Adeloye, C E Brightling, J S Brown, J Busby, J D Chalmers, C Echevarria, L Daines, O Elneima, RA Evans, J Hurst, P Novotny, C Nicolaou, P Pfeffer, K Poinasamy, J Quint, I Rudan, E Sapey, M Shankar-Hari, A Sheikh, S Siddiqui, S Walker, B Zheng

Brain

J R Geddes (*Lead*), M Hotopf *(Co-Lead),* K Abel, R Ahmed, L Allan, C Armour, D Baguley, D Baldwin, C Ballard, K Bhui, G Breen, K Breeze, M Broome, T Brugha, E Bullmore, D Burn, F Callard, J Cavanagh, T Chalder, D Clark, A David, B Deakin, H Dobson, B Elliott, J Evans, RA Evans, R Francis, E Guthrie, P Harrison, M Henderson, A Hosseini, N Huneke, M Husain, T Jackson, I Jones, T Kabir, P Kitterick, A Korszun, I Koychev, J Kwan, A Lingford-Hughes, P Mansoori, H McAllister-Williams, K McIvor, B Michael, L Milligan, R Morriss, E Mukaetova-Ladinska, K Munro, A Nevado-Holgado, T Nicholson, C Nicolaou, S Paddick, C Pariante, J Pimm, K Saunders, M Sharpe, G Simons, J P Taylor, R Upthegrove, S Wessely

Cardiac

G P McCann (*Lead*), S Amoils, C Antoniades, A Banerjee, A Bularga, C Berry, P Chowienczyk, J P Greenwood, A D Hughes, K Khunti, C Lawson, N L Mills, A J Moss, S Neubauer, B Raman, A N Sattar, C L Sudlow, M Toshner,

Immunology

P J M Openshaw (*Lead*), D Altmann, J K Baillie, R Batterham, H Baxendale, N Bishop, C E Brightling, P C Calder, R A Evans, J L Heeney, T Hussell, P Klenerman, J M Lord, P Moss, S L Rowland-Jones, W Schwaeble, M G Semple, R S Thwaites, L Turtle, L V Wain, S Walmsley, D Wraith

Intensive Care

M J Rowland (*Lead*), A Rostron (*Co-Lead*), J K Baillie, B Connolly, A B Docherty, N I Lone, D F McAuley, D Parekh, A Rostron, J Simpson, C Summers

Lung Fibrosis

R G Jenkins (*Co-Lead*), J Porter (*Co-Lead*), R J Allen, R Aul, J K Baillie, S Barratt, P Beirne, J Blaikley, R C Chambers, N Chaudhuri, C Coleman, E Denneny, L Fabbri, P M George, M Gibbons, F Gleeson, B Gooptu, B Guillen Guio, I Hall, N A Hanley, L P Ho, E Hufton, J Jacob, I Jarrold, G Jenkins, S Johnson, M G Jones, S Jones, F Khan, P Mehta, J Mitchell, P L Molyneaux, J E Pearl, K Piper Hanley, K Poinasamy, J Quint, D Parekh, P Rivera-Ortega, L C Saunders, M G Semple, J Simpson, D Smith, M Spears, L G Spencer, S Stanel, I Stewart, A A R Thompson, D Thickett, R Thwaites, L V Wain, S Walker, S Walsh, J M Wild, D G Wootton, L Wright

Metabolic

S Heller (*Co-Lead*), M J Davies (*Co-Lead*), H Atkins, S Bain, J Dennis, K Ismail, D Johnston, P Kar, K Khunti, C Langenberg, P McArdle, A McGovern, T Peto, J Petrie, E Robertson, N Sattar, K Shah, J Valabhji, B Young

Pulmonary and Systematic Vasculature

L S Howard (*Co-Lead*), Mark Toshner (*Co-Lead*), C Berry, P Chowienczyk, D Lasserson, A Lawrie, O C Leavy, J Mitchell, J Newman, L Price, J Quint, A Reddy, J Rossdale, N Sattar, C Sudlow, A A R Thompson, J M Wild, M Wilkins

Rehabilitation, Sarcopenia and Fatigue

S J Singh (*Co-Lead*), W D-C Man (*Co-Lead*), J M Lord (*Co-Lead*), N J Greening (*Co-Lead*), T Chalder (*Co-Lead*), J T Scott (*Co-Lead*), N Armstrong, E Baldry, M Baldwin, N Basu, M Beadsworth, L Bishop, C E Bolton, A Briggs, M Buch, G Carson, J Cavanagh, H Chinoy, C Dawson, E Daynes, S Defres, R A Evans, L Gardiner, P Greenhaff, S Greenwood, M Harvie, L HOuchen-Wolloff, M Husain, S MacDonald, A McArdle, H J C McAuley, A McMahon, M McNarry, G Mills, C Nolan, K O'Donnell, D Parekh, Pimm, J Sargent, L Sigfrid, M Steiner, D Stensel, A L Tan, I Vogiatzis, J Whitney, D Wilkinson, D Wilson, M Witham, D G Wootton, T Yates

Renal

D Thomas (*Lead*), N Brunskill (*Co-Lead*), S Francis (*Co-Lead*), S Greenwood (*Co-Lead*), C Laing (*Co-Lead*), K Bramham, P Chowdhury, A Frankel, L Lightstone, S McAdoo, K McCafferty, M Ostermann, N Selby, C Sharpe, M Willicombe

Patient Public Engagement Group

L Houchen-Wolloff (*Lead*), J Bunker, R Gill, C Hastie, R Nathu, N Rogers, N Smith

Local Clinical Centre PHOSP-COVID trial staff

(listed in alphabetical order)

Airedale NHS Foundation Trust

A Shaw (PI), L Armstrong, B Hairsine, H Henson, C Kurasz, L Shenton

Aneurin Bevan University Health Board

S Fairbairn (PI), A Dell, N Hawkings, J Haworth, M Hoare, A Lucey, V Lewis, G Mallison, H Nassa, C Pennington, A Price, C Price, A Storrie, G Willis, S Young

Barts Health NHS Trust & Queen Mary University of London

P Pfeffer (PI), K Chong-James, C David, W Y James, C Manisty, A Martineau, O Zongo

Barnsley Hospital NHS Foundation Trust

A Sanderson (PI)

Belfast Health and Social Care Trust & Queen's University Belfast

L G Heaney (PI), C Armour, V Brown, T Craig, S Drain, B King, N Magee, D McAulay, E Major, L McGarvey, J McGinness, R Stone

Betsi Cadwaladr University Health Board

A Haggar (PI), A Bolger, F Davies, J Lewis, A Lloyd, R Manley, E McIvor, D Menzies, K Roberts, W Saxon, D Southern, C Subbe, V Whitehead

Borders General Hospital, NHS Borders

H El-Taweel (PI), J Dawson, L Robinson

Bradford Teaching Hospitals NHS Foundation Trust

D Saralaya (PI), L Brear, K Regan, K Storton

Cambridge University Hospitals NHS Foundation Trust, NIHR Cambridge Clinical Research Facility & University of Cambridge
J Fuld (PI), A Bermperi, I Cruz, K Dempsey, A Elmer, H Jones, S Jose, S Marciniak, M Parkes, C Ribeiro, J Taylor, M Toshner, L Watson, J Weir McCall, J Worsley

Cardiff and Vale University Health Board

R Sabit (PI), L Broad, A Buttress, T Evans, M Haynes, L Jones, L Knibbs, A McQueen, C Oliver, K Paradowski, J Williams

Chesterfield Royal Hospital NHS Trust

E Harris (PI), C Sampson

Cwm Taf Morgannwg University Health Board

C Lynch (PI), E Davies, C Evenden , A Hancock, K Hancock, M Rees , L Roche, N Stroud, T Thomas-Woods

East Cheshire NHS Trust

M Babores (PI), J Bradley-Potts, M Holland, N Keenan, S Shashaa, H Wassall

East Kent Hospitals University NHS Foundation Trust

E Beranova (PI), H Weston (PI), T Cosier, L Austin, J Deery, T Hazelton, C Price, H Ramos, R Solly, S Turney

Gateshead NHS Trust

L Pearce (PI), W McCormick, S Pugmire, W Stoker, A Wilson

Guy's and St Thomas' NHS Foundation Trust
N Hart (PI), LA Aguilar Jimenez, G Arbane, S Betts, K Bisnauthsing, A Dewar, P Chowdhury, A Chiribiri, A Dewar, G Kaltsakas, H Kerslake, MM Magtoto, P Marino, LM Martinez, C O'Brien, M Ostermann, J Rossdale, TS Solano, E Wynn

Hampshire Hospitals NHS Foundation Trust
N Williams (PI), W Storrar (PI), M Alvarez Corral, A Arias, E Bevan, D Griffin, J Martin, J Owen, S Payne, A Prabhu, A Reed, C Wrey Brown

Harrogate and District NHD Foundation Trust

C Lawson (PI), T Burdett, J Featherstone, A Layton, C Mills, L Stephenson,

Hull University Teaching Hospitals NHS Trust & University of Hull
N Easom (PI), P Atkin, K Brindle, M G Crooks, K Drury, R Flockton, L Holdsworth, A Richards, D L Sykes, S Thackray-Nocera, C Wright

Hywel Dda University Health Board

K E Lewis (PI), A Mohamed (PI), G Ross (PI), S Coetzee, K Davies, R Hughes, R Loosley, L O'Brien, Z Omar, H McGuinness, E Perkins, J Phipps, A Taylor, H Tench, R Wolf-Roberts

Imperial College Healthcare NHS Trust & Imperial College London
L S Howard (PI), O Kon (PI), D C Thomas (PI), S Anifowose, L Burden, E Calvelo, B Card, C Carr, E R Chilvers, D Copeland, P Cullinan, P Daly, L Evison, T Fayzan, H Gordon, S Haq, R G Jenkins, C King, K March, M Mariveles, L McLeavey, N Mohamed, S Moriera, U Munawar, J Nunag, U Nwanguma, L Orriss- Dib, D P O'Regan, A Ross, M Roy, E Russell, K Samuel, J Schronce, N Simpson, L Tarusan, C Wood, N Yasmin

Kettering General Hospital NHS Trust

R Reddy (PI), A-M, Guerdette, M Hewitt, K Warwick, S White

King's College Hospital NHS Foundation Trust & Kings College London
A M Shah (PI), C J Jolley (PI), O Adeyemi, R Adrego, H Assefa-Kebede, J Breeze, M Brown, S Byrne, T Chalder, A Chiribiri, P Dulawan, N Hart, A Hayday, A Hoare, A Knighton, M Malim, C O'Brien, S Patale, I Peralta, N Powell, A Ramos, K Shevket, F Speranza, A Te

Leeds Teaching Hospitals & University of Leeds
P Beirne (PI), A Ashworth, J Clarke, C Coupland, M Dalton, E Wade, C Favager, J Greenwood, J Glossop, L Hall, T Hardy, A Humphries, J Murira, D Peckham, S Plein, J Rangeley, G Saalmink, A L Tan, B Whittam, N Window, J Woods,

Lewisham & Greenwich NHS Trust

G Coakley (PI)

Liverpool University Hospitals NHS Foundation Trust & University of Liverpool

D G Wootton (PI), L Turtle (PI), L Allerton, AM All, M Beadsworth, A Berridge, J Brown, S Cooper, A Cross, D J Cuthbertson, S Defres, S L Dobson, J Earley, N French, W Greenhalf, H E Hardwick, K Hainey, J Hawkes, V Highett, S Kaprowska, G J Kemp, AL Key, S Koprowska, L Lavelle-Langham, N Lewis-Burke, G Madzamba, F Malein, S Marsh, C Mears, L Melling, M J Noonan, L Poll, J Pratt, E Richardson, A Rowe, M G Semple, V Shaw, K A Tripp, B Vinson, L O Wajero, S A Williams-Howard, J Wyles

London North West University Healthcare NHS Trust
S N Diwanji (PI), P Papineni (PI), S Gurram, S Quaid, G F Tiongson, E Watson

Manchester University NHS Foundation Trust & University of Manchester
B Al-Sheklly (PI), A Horsley (PI), C Avram, J Blaikely, M Buch, N Choudhury, D Faluyi, T Felton, T Gorsuch, N A Hanley, T Hussell, Z Kausar, C A Miller, N Odell, R Osbourne, K Piper Hanley, K Radhakrishnan, S Stockdale

Newcastle upon Tyne Hospitals NHS Foundation Trust & University of Newcastle
A De Soyza (PI), C Echevarria (PI), A Ayoub, J Brown, G Burns, G Davies, H Fisher, C Francis, A Greenhalgh, P Hogarth, J Hughes, K Jiwa, G Jones, G MacGowan, D Price, A Sayer, J Simpson, H Tedd, S Thomas, S West, M Witham, S Wright, A Young

NHS Dumfries and Galloway
M J McMahon (PI), P Neill

NHS Greater Glasgow and Clyde Health Board & University of Glasgow
D Anderson (PI), H Bayes (PI), C Berry (PI), D Grieve (PI), I B McInnes (PI), N Basu, A Brown, A Dougherty, K Fallon, L Gilmour, K Mangion, A Morrow, K Scott, R Sykes, R Touyz

NHS Highland
E K Sage (PI), F Barrett, A Donaldson

NHS Lanarkshire
M Patel (PI), D Bell, A Brown, M Brown, R Hamil, K Leitch, L Macliver, J Quigley, A Smith, B Welsh

NHS Lothian & University of Edinburgh

G Choudhury (PI), J K Baillie, S Clohisey, A Deans, A B Docherty, J Furniss, E M Harrison, S Kelly, N I Lone, D E Newby, A Sheikh

NHS Tayside & University of Dundee
J D Chalmers (PI), D Connell, A Elliott, C Deas, J George, S Mohammed, J Rowland, A R Solstice, D Sutherland, C J Tee

North Bristol NHS Trust & University of Bristol

N Maskell (PI), D Arnold, S Barrett, H Adamali, A Dipper, S Dunn, A Morley, L Morrison, L Stadon, S Waterson, H Welch

North Middlesex Hospital NHS Trust
B Jayaraman (PI), T Light

Nottingham University Hospitals NHS Trust & University of Nottingham
C E Bolton (PI), P Almeida, J Bonnington, M Chrystal, E Cox, C Dupont, S Francis, P Greenhaff, A Gupta, L Howard, W Jang, S Linford, L Matthews, R Needham, A Nikolaidis, S Prosper, K Shaw, A K Thomas

Oxford University Hospitals NHS Foundation Trust & University of Oxford
L P Ho (PI), N M Rahman (PI), M Ainsworth, A Alamoudi, M Beggs, A Bates, A Bloss, A Burns, P Carter, M Cassar, K M Channon, J Chen, F Conneh, T Dong, R I Evans, E Fraser, X Fu, J R Geddes, F Gleeson, P Harrison, M Havinden-Williams, P Jezzard, N Kanellakis, I Koychev, P Kurupati, X Li, E Lukaschuk, K McGlynn, H McShane, C Megson, K Motohashi, S Neubauer, D Nicoll, G Ogg, E Pacpaco, M Pavlides, Y Peng, N Petousi, J Propescu, N Rahman, B Raman, M J Rowland, K Saunders, M Sharpe, N Talbot, E Tunnicliffe

Royal Brompton and Harefield Clinical Group, Guy's and St Thomas' NHS Foundation Trust.

W D-C Man (PI), B Patel (PI), R E Barker, D Cristiano, N Dormand, M Gummadi, S Kon, K Liyanage, C M Nolan, S Patel, O Polgar, P Shah, S J Singh, J A Walsh

Royal Free London NHS Foundation Trust
J Hurst (PI), H Jarvis (PI), S Mandal (PI), S Ahmad, S Brill, L Lim, D Matila, O Olaosebikan, C Singh

Royal Papworth Hospital NHS Foundation Trust

M Toshner (PI), H Baxendale, L Garner, C Johnson, J Mackie, A Michael, J Pack, K Paques, H Parfrey, J Parmar

Salford Royal NHS Foundation Trust
N Diar Bakerly (PI), P Dark, D Evans, E Hardy, A Harvey, D Holgate, S Knight, N Mairs, N Majeed, L McMorrow, J Oxton, J Pendlebury, C Summersgill, R Ugwuoke, S Whittaker

Salisbury NHS Foundation Trust

W Matimba-Mupaya (PI), S Strong-Sheldrake

Sheffield Teaching NHS Foundation Trust & University of Sheffield

S L Rowland-Jones (PI), A A R Thompson (Co PI), J Bagshaw, M Begum, K Birchall, R Butcher, H Carborn, F Chan, K Chapman, Y Cheng, L Chetham, C Clark, Z Coburn, J Cole, M Dixon, A Fairman, J Finnigan, L Finnigan, H Foot, D Foote, A Ford, R Gregory, K Harrington, L Haslam, L Hesselden, J Hockridge, A Holbourn, B Holroyd-Hind, L Holt, A Howell, E Hurditch, F Ilyas, C Jarman, A Lawrie, E Lee, J-H Lee, R Lenagh, A Lye, I Macharia, M Marshall, A Mbuyisa, J McNeill, S Megson, J Meiring, L Milner, S Misra, H Newell, T Newman, C Norman, L Nwafor, D Pattenadk, M Plowright, J Porter, P Ravencroft, C Roddis, J Rodger, P Saunders, J Sidebottom, J Smith, L Smith, N Steele, G Stephens, R Stimpson, B Thamu, N Tinker, K Turner, H Turton, P Wade, S Walker, J Watson, J M Wild, I Wilson, A Zawia

St George's University Hospitals NHS Foundation Trust
R Aul (PI), M Ali, A Dunleavy (PI), D Forton, N Msimanga, M Mencias, T Samakomva, S Siddique, J Teixeira, V Tavoukjian

Sherwood Forest Hospitals NHS Foundation Trust
J Hutchinson (PI), L Allsop, K Bennett, P Buckley, M Flynn, M Gill, C Goodwin, M Greatorex, H Gregory, C Heeley, L Holloway, M Holmes, J Kirk, W Lovegrove, TA Sewell, S Shelton, D Sissons, K Slack, S Smith, D Sowter, S Turner, V Whitworth, I Wynter

Shropshire Community Health NHS Trust
L Warburton (PI), S Painter, J Tomlinson

Somerset NHS Foundation Trust
C Vickers (PI), T Wainwright, D Redwood, J Tilley, S Palmer

Swansea Bay University Health Board
G A Davies (PI), L Connor, A Cook, T Rees, F Thaivalappil, C Thomas

Tameside and Glossop Integrated Care NHS Foundation

A Butt (PI), M Coulding, H Jones, S Kilroy, J McCormick, J McIntosh, H Savill, V Turner, J Vere

The Great Western Hospital Foundation Trust

E Fraile (PI), J Ugoji

The Hillingdon Hospitals NHS Foundation Trust
S S Kon (PI), H Lota, G Landers, M Nasseri, S Portukhay

The Rotherham NHS Foundation Trust

A Hormis (PI), A Daniels, J Ingham, L Zeidan

United Lincolnshire Hospitals NHS Trust
M Chablani (PI), L Osborne

University College London Hospital & University College London
M Marks (PI), J S Brown (PI), N Ahwireng, B Bang, D Basire, R C Chambers, A Checkley, R Evans, M Heightman, T Hillman, J Hurst, J Jacob, S Janes, R Jastrub, M Lipman, S Logan, D Lomas, M Merida Morillas, A Pakzad, H Plant, J C Porter, K Roy, E Wall, B Williams, M Xu

University Hospital Birmingham NHS Foundation Trust & University of Birmingham
D Parekh (PI), N Ahmad Haider, C Atkin, R Baggott, M Bates, A Botkai, A Casey, B Cooper, J Dasgin, K Draxlbauer, N Gautam,

J Hazeldine, T Hiwot, S Holden, K Isaacs, T Jackson, S Johnson, V Kamwa, D Lewis,

J M Lord, S Madathil, C McGhee, K Mcgee, A Neal, A Newton Cox, J Nyaboko, D Parekh, Z Peterkin, H Qureshi, B Rangelov, L Ratcliffe, E Sapey, J Short, T Soulsby, R Steeds, J Stockley, Z Suleiman, T Thompson, M Ventura, S Walder, C Welch, D Wilson, S Yasmin, K P Yip

University Hospitals of Derby and Burton

P Beckett (PI) C Dickens, U Nanda

University Hospitals of Leicester NHS Trust & University of Leicester

C E Brightling (CI), R A Evans (PI), M Aljaroof, N Armstrong, H Arnold, H Aung, M Bakali, M Bakau, M Baldwin, M Bingham, M Bourne, C Bourne, N Brunskill, P Cairns, L Carr, A Charalambou, C Christie, M J Davies, S Diver, S Edwards, C Edwardson, O Elneima, H Evans, J Finch, S Glover, N Goodman, B Gootpu, N J Greening, K Hadley, P Haldar, B Hargadon, V C Harris, L Houchen-Wolloff, W Ibrahim, L Ingram, K Khunti, A Lea, D Lee, G P McCann, H J C McAuley, P McCourt, T Mcnally, G

Mills, A Moss, W Monteiro, M Pareek, S Parker, A Rowland, A Prickett, I N Qureshi, R Russell, N Samani, M Sereno, M Sharma, A Shikotra, S Siddiqui, A Singapuri, S J Singh, J Skeemer, M Soares, E Stringer, T Thornton, M Tobin, E Turner, L V Wain, T J C Ward, F Woodhead, J Wormleighton, T Yates, A Yousuf

University Hospital Southampton NHS Foundation Trust & University of Southampton
M G Jones (PI), C Childs, R Djukanovic, S Fletcher, M Harvey, E Marouzet, B Marshall, R Samuel, T Sass, T Wallis, H Wheeler

Whittington Health NHS
R Dharmagunawardena (PI), E Bright, P Crisp, M Stern

Wirral University Teaching Hospital

A Wight (PI), L Bailey, A Reddington

Wrightington Wigan and Leigh NHS trust

A Ashish (PI), J Cooper, E Robinson

Yeovil District Hospital NHS Foundation Trust

A Broadley (PI)

York & Scarborough NHS Foundation Trust

K Howard (PI), L Barman, C Brookes, K Elliott. L Griffiths, Z Guy, D Ionita, H Redfearn, C Sarginson

A Turnbull

Health and Care Research Wales

Y Ellis

London School of Hygiene & Tropical Medicine (LSHTM)
M Marks, A Briggs

NIHR Office for Clinical Research Infrastructure

K Holmes

Patient Public Involvement Leads

Asthma UK and British Lung Foundation Partnership - K Poinasamy, S Walker

Royal Surrey NHS Foundation Trust
M Halling-Brown

South London and Maudsley NHS Foundation Trust & Kings College London
G Breen, M Hotopf

Swansea University & Swansea Welsh Network

K Lewis, N Williams

Supplementary Notes

Supplementary Note 1

CCA seeks to identify the relatively complex biomarker profiles that best correlate with cognitive profiles. While it is informative to think of those biomarker profiles as being driven by specific biomarkers (e.g. by raised fibrinogen relative to CRP for the first dimension), such a simplified view might fail to capture the complexity of the biomarker profile necessary to predict cognitive outcomes. In this supplementary note, we explore the simplest representation of the dimension of covariation that can still reliably reproduce the main results from the manuscript.

One possibility would be that dimensions of covariation imply associations at the univariate level (i.e. that high fibrinogen and high D-dimer themselves predict cognitive outcomes). However, we found this not to be the case. Adjusting for all covariates as in the primary analysis, fibrinogen was significantly associated with neither C-PSQ nor MoCA at 6-months ($p>0.05$ for both), and neither was D-dimer ($p>0.05$ for both). One reason might be that such univariate associations fail to encode the role of CRP in the biomarker profile.

A simplified representation of the dimensions of covariation which captures the role of CRP is to simplify the biomarker profile to be the difference between fibrinogen and CRP for the first dimension of covariation and the difference between D-dimer and CRP for the second dimension of covariation (more specifically the difference between covariate-adjusted and, for D-dimer and CRP, log-transformed biomarker values). This amounts to setting the weights of the weighted combinations representing biomarker profiles to 1 for fibrinogen (respectively D-dimer for the second dimension) and -1 for CRP, while setting all other weights to zero. These simplified biomarker profiles were found to be significantly associated with C-PSQ at 6 months ($r=0.09$ and $p<0.0001$ for both dimensions). This implies that simplified biomarker profiles in which only fibrinogen and CRP (respectively D-dimer and CRP for the second dimension) are kept and in which their relative contributions are given equal weights are enough to capture the association between biomarkers and subjective cognitive deficit. Interestingly, the simplified profile for the first dimension of covariation was not significantly associated with the MoCA at 6-months, and the full dimension of covariation was needed to

capture this association (as in the primary analysis). This might in part reflect the lower effect size of the association with MoCA.

Supplementary Note 2

The blood biomarkers used in this study are routinely collected in clinical practice and tests were performed by clinical laboratories within the National Health Service (NHS). These laboratories and the equipment that they use are subject to strict standards in terms of calibration, reliability, and accuracy of results (specifically through ISO 15189:2012 standards).

In addition, as per the PHOSP protocol (which is publicly available as part of Appendix 2 of the first PHOSP-COVID study[1]):

1) Outlying values were investigated in the raw data and excluded as appropriate. This was done before the current study was initiated.
2) Data quality was maintained using tools within the REDCap environment and through dashboards provided by the REDCap host site. These ensure data quality at individual sites is maintained and allow early notification of problems. Regular reviews of the data quality were undertaken by dedicated PHOSP team members.

Supplementary Note 3

The cognitive subset of the Patient Symptom Questionnaire (C-PSQ) is a score ranging from 0 to 7 which we encoded as a continuous variable. Specifically, the score was defined as the sum of the following items from PSQ:

- Within the last seven days, have you had any of these symptoms…?
    - Confusion (Yes: +1 point, No: 0 point)
    - Short term memory loss (Yes: +1 point, No: 0 point)

    - Difficulty with concentration (Yes: +1 point, No: 0 point)
    - Difficulty with communication (Yes: +1 point, No: 0 point)
    - Slowing down in your thinking (Yes: +1 point, No: 0 point)
- Do you have difficulty remembering or concentrating?:
    - No: 0 points
    - Yes, some difficulty: + 1/3 point
    - Yes, a lot of difficulty: + 2/3 points
    - Yes, cannot do at all: +1 point
- Do you have difficulty communicating, for example understanding or being understood?
  - No: 0 points
  - Yes, some difficulty: + 1/3 point
  - Yes, a lot of difficulty: + 2/3 points
  - Yes, cannot do at all: +1 point

Supplementary Note 4

The effect of confounding in the replication study with EHR data was reduced with 1:1 matching on the following factors (provided with ICD-10/CDC codes in brackets):

1) **Age** at the time of index event.

2) **Sex** coded as female, male, or other.

3) **Race** encoded as 6 separate dichotomous variables: White (2106-3), Black or African American (2054-5), American Indian or Alaska Native (1002-5), Asian (2028-9), Native Hawaiian or Other Pacific Islander (2076-8), or Unknown Race (2131-1).

4) **Ethnicity** encoded as Hispanic or Latino (2135-2), Not Hispanic or Latino (2186-5), or Unknown Ethnicity.

5) **Socioeconomic deprivation** encoded as the ICD-10 code for Problems related to housing and economic circumstances (Z59).

6) **Obesity** encoded as Overweight and obesity (E66)

7) **Hypertension** encoded as 2 dichotomous variables: Hypertensive diseases (I10-I16) and the now deprecated version that was used until 2018 Hypertension diseases (I10-I15).

8) **Diabetes mellitus** encoded as 2 dichotomous variables: Type 1 diabetes mellitus (E10) and Type 2 diabetes mellitus (E11).

9) **Chronic lower respiratory diseases** encoded by each sub-category of the corresponding ICD-10 group: Bronchitis, not specified as acute or chronic (J40), Simple and mucopurulent chronic bronchitis (J41), Unspecified chronic bronchitis (J42), Emphysema (J43), Other chronic obstructive pulmonary disease (J44), Asthma (J45), Bronchiectasis (J47).

10) **Nicotine dependence** encoded as the corresponding ICD-10 diagnosis (F17.2).

11) **Substance use disorders** encoded as the ICD-10 code for mental and behavioural disorders due to psychoactive substance use (F10-F19).

12) **Psychotic disorders** encoded as the ICD-10 code for schizophrenia, schizotypal, delusional, and other non-mood psychotic disorders (F20-F29).

13) **Mood disorders** encoded as a single variable (as well as individual codes, see below) with any of the ICD10 code for mood disorders (F30-F39).

14) **Anxiety disorders** encoded as the ICD-10 code for anxiety, dissociative, stress-related, somatoform and other nonpsychotic mental disorders (F40-F48)

15) **Heart diseases** encoded as 2 categorical variables: Ischaemic heart disease (I20-I25) and Other forms of heart disease (I30-I52).

16) **Chronic kidney disease** encoded as 2 dichotomous variables: Chronic kidney disease (N18) and Hypertensive chronic kidney disease (I12).

17) **Chronic liver disease** encoded as 8 categorical variables: Alcoholic liver disease (K70), Hepatic failure, not elsewhere classified (K72), Chronic hepatitis, not elsewhere classified (K73), Fibrosis and cirrhosis of 6 liver (K74), Fatty (change of) liver, not elsewhere classified (K76.0), Chronic passive congestion of liver (K76.1), Portal hypertension (K76.6), Other specified diseases of liver (K76.8).

18**) Stroke** encoded as the dichotomous variable Cerebral infarction (I63) .

19) **Dementia** encoded as 6 dichotomous variables: Vascular dementia (F01), Dementia in other diseases classified elsewhere (F02), Unspecified dementia (F03), Alzheimer's disease (G30), Frontotemporal dementia (G31.0), and Dementia with Lewy bodies (G31.83).

20) **Neoplasm (benign or malignant) and haematological cancer in particular** encoded as 2 dichotomous variables: Neoplasms (C00-D49) and Malignant neoplasms of lymphoid, hematopoietic and related tissue (C81-C96).

21) **Organ transplant** encoded as 2 dichotomous variables: Renal Transplantation Procedures and Liver Transplantation Procedures.

22) **Rheumatoid arthritis** encoded as 2 dichotomous variables: Rheumatoid arthritis with rheumatoid factor (M05) and Other rheumatoid arthritis (M06).

23) **Systemic lupus erythematosus** encoded as a dichotomous variable corresponding ICD-10 code (M32).

24) **Psoriasis** encoded as a dichotomous variable corresponding ICD-10 code (L40).

25) **Disorders involving an immune mechanism** encoded as a dichotomous variable "Certain disorders involving the immune mechanism" (D80-D89).

26) **Insomnia** encoded as 2 dichotomous variables: Insomnia (G47.0) and Insomnia not due to a substance or known physiological condition (F51.0)

27) **Intracranial haemorrhage** encoded as 3 dichotomous variables: Nontraumatic subarachnoid haemorrhage (I60), Nontraumatic intracerebral haemorrhage (I61) and Other and unspecified nontraumatic intracranial haemorrhage (I62).

28) **Parkinsonism** encoded as 2 dichotomous variables: Parkinson's disease (G20) and Secondary parkinsonism (G21).

29) **Peripheral neurologic disorders** encoded as 3 dichotomous variables: Guillan-Barre syndrome (G61.0), Nerve, nerve root and plexus disorders (G50-G59) and Diseases of myoneural junction and muscle (G70-G73).

30) **Encephalopathy and encephalitis** encoded as 5 dichotomous variables: Encephalopathy, unspecified (G93.40), Encephalitis, myelitis and encephalomyelitis (G04), Encephalitis, myelitis and encephalomyelitis

in diseases classified elsewhere (G05), Unspecified viral encephalitis (A86) and Other specified viral encephalitis (A85.8).

31) **Cognitive impairment and mental disorders** encoded as 3 dichotomous variables: Mild cognitive impairment, so stated (G31.84), Dyslexia and other symbolic dysfunctions, not elsewhere classified (R48), Other specified mental disorders due to known physiological condition (F06.8).

32) **Altered mental state** encoded as 3 dichotomous variables: Delirium due to known physiological condition (F05), Somnolence, stupor, coma (R40), Other symptoms and signs involving cognitive functions and awareness (R41).

33) **Epilepsy and seizures** encoded as 2 dichotomous variables: Epilepsy and recurrent seizures (G40) and Convulsions, not elsewhere classified (R56).

34) **Antipsychotics** as a class, encoded as VA Class CN700

35) **Clozapine** encoded as RxNorm 2626

36) **Antidepressants** as a class, encoded as VA Class CN600

37) **Fluvoxamine** encoded as RxNorm 42355

38) **Lithium salts** encoded as VA Class CN750

39) **SARS-CoV-2** vaccination encoded as 4 dichotomous variables:

a) SARS-CoV-2 (covid-19) vaccine, unspecified encoded as CVX 213

b) Pfizer's Severe acute respiratory syndrome coronavirus 2 (SARS-CoV-2) (Coronavirus disease [COVID-19]) vaccine, mRNA-LNP, spike protein, preservative free, 30 mcg/0.3mL dosage, diluent reconstituted, for intramuscular use, encoded as CPT 91300

c) Moderna's Severe acute respiratory syndrome coronavirus 2 (SARS-CoV-2) (Coronavirus disease [COVID-19]) vaccine, mRNA-LNP, spike protein, preservative free, 100 mcg/0.5mL dosage, for intramuscular use, encoded as CPT 91301

d) Janssen's Severe acute respiratory syndrome coronavirus 2 (SARS-CoV-2) (Coronavirus disease [COVID-19]) vaccine, DNA, spike protein, adenovirus type 26 (Ad26) vector, preservative free, 5x10^10 viral particles/0.5mL dosage, for intramuscular use, encoded as CPT 91303.

Supplementary Figures

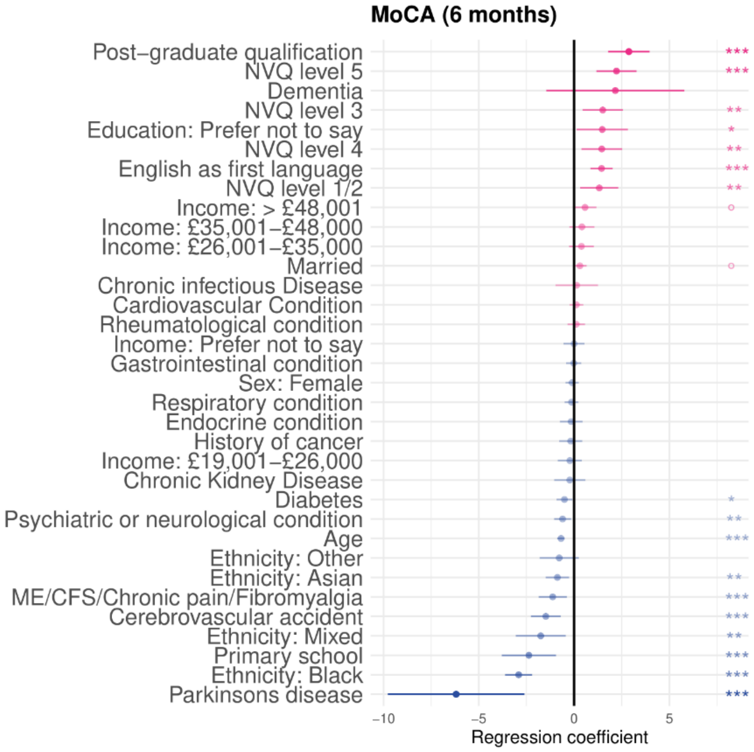

Supplementary Figure 1: Association between baseline characteristics and MoCA at 6 months (measuring objective cognitive deficits, lower indicate more deficits). Age was z-transformed in this analysis which means that the coefficient corresponds to a difference in MoCA corresponding to a difference by 1 standard deviation in age. ME/CFS= Myalgic Encephalomyelitis/Chronic fatigue syndrome. n=1837 individual participants. Dots indicate point estimates and horizontal lines indicate 95% confidence intervals. P-values were estimated as part of the generalised linear model and are two-sided and not adjusted for multiple comparisons: ° p<0.1, * p<0.05, ** p<0.01, *** p<0.001.

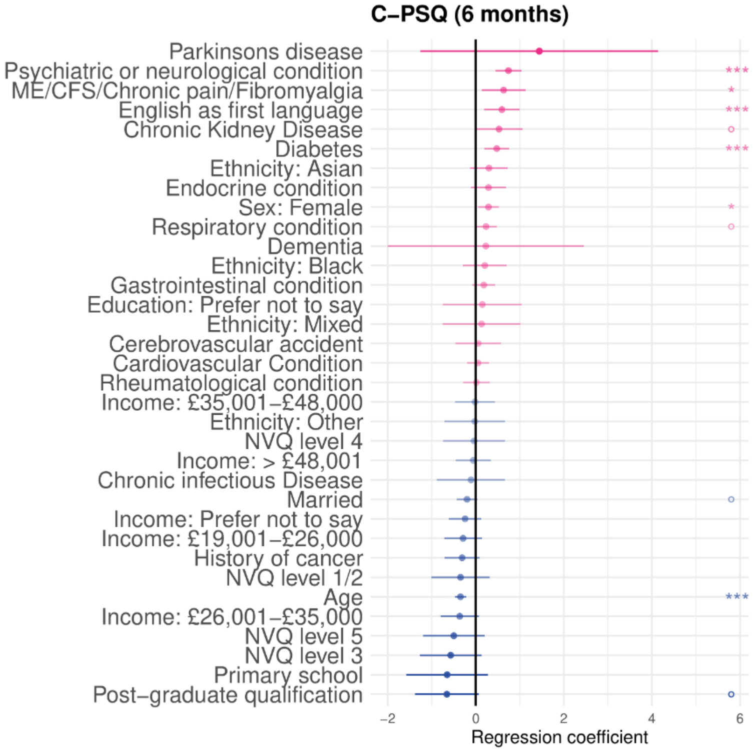

Supplementary Figure 2: Association between baseline characteristics and C-PSQ at 6 months (measuring subjective cognitive deficits, higher values indicate more deficits). Age was z-transformed in this analysis which means that the coefficient corresponds to a difference in C-PSQ corresponding to a difference by 1 standard deviation in age. ME/CFS= Myalgic Encephalomyelitis/Chronic fatigue syndrome. n=1502 individual participants. Dots indicate point estimates and horizontal lines indicate 95% confidence intervals. P-values were estimated as part of the generalised linear model and are two-sided and not adjusted for multiple comparisons: ° p<0.1, * p<0.05, ** p<0.01, *** p<0.001.

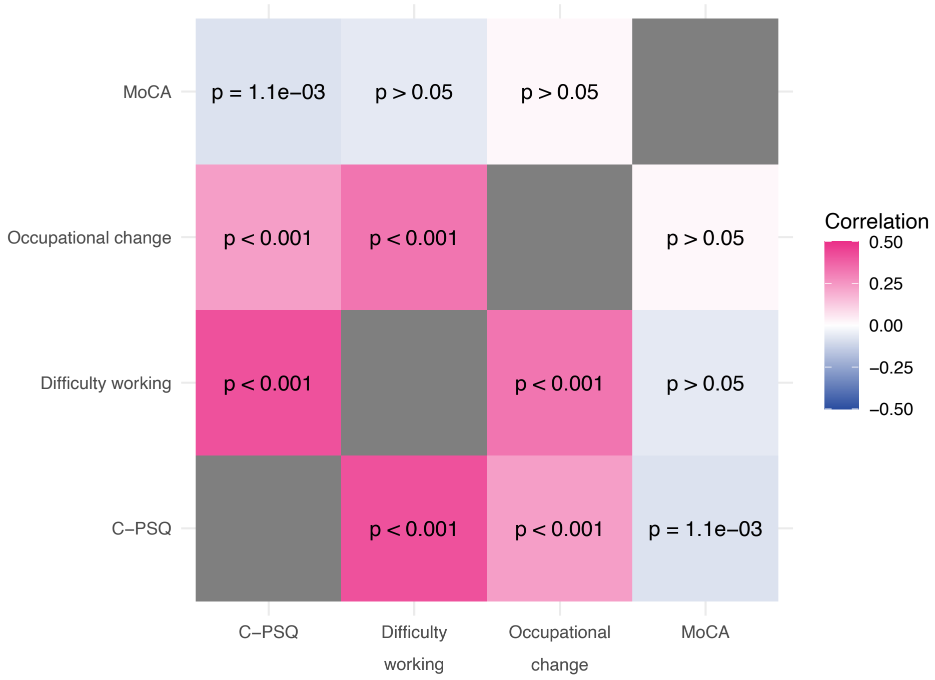

Supplementary Figure 3: Correlations between the different outcome variables (i.e. objective and subjective cognitive deficits and occupational outcomes) all measured at 6-months post-COVID-19.

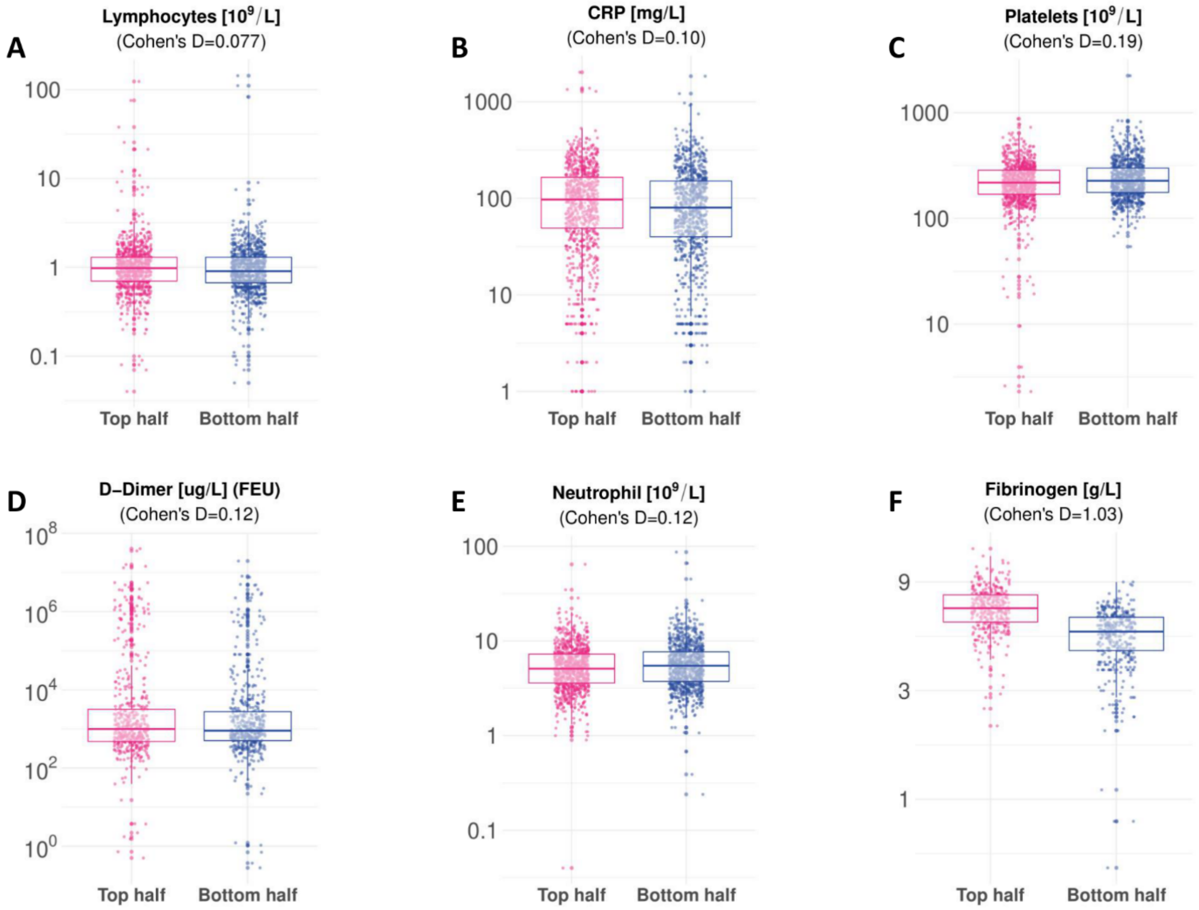

Supplementary Figure 4: Distribution of biomarkers between the top half and the bottom half of the cohort along the first dimension of covariation. Each of the six biomarkers are represented and only complete data are displayed (n=1818, 1777, 1810, 977, 1796, 768 individual participants respectively). The centre of the boxes represent the median, their bounds represent the 25th and 75th centile and the lower and upper ends of whiskers represent the smallest/largest value, no further than 1.5 times the interquartile range from the boxplot respective end.

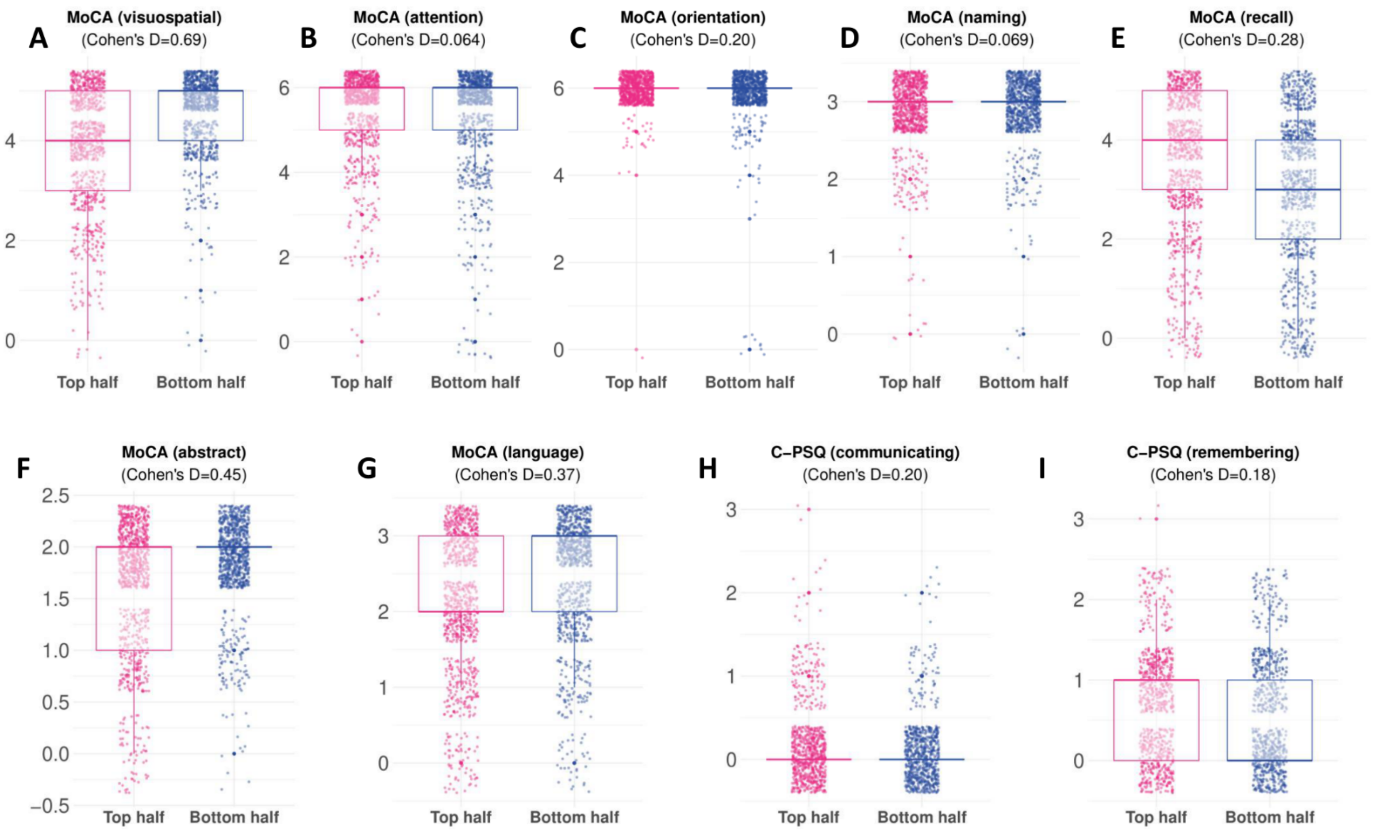

Supplementary Figure 5: Distribution of test scores between the top half and the bottom half of the cohort along the first dimension of covariation for different items of the MoCA and the numerical part of the C-PSQ score measured at the 6-months follow-up. Higher C-PSQ scores and lower MoCA scores represent more deficits. Only complete data are displayed (n=1837 individual participants for A-G and 1537, 1544 for H and I, respectively). The centre of the boxes represent the median, their bounds represent the 25th and 75th centile and the lower and upper ends of whiskers represent the smallest/largest value, no further than 1.5 times the interquartile range from the boxplot respective end.

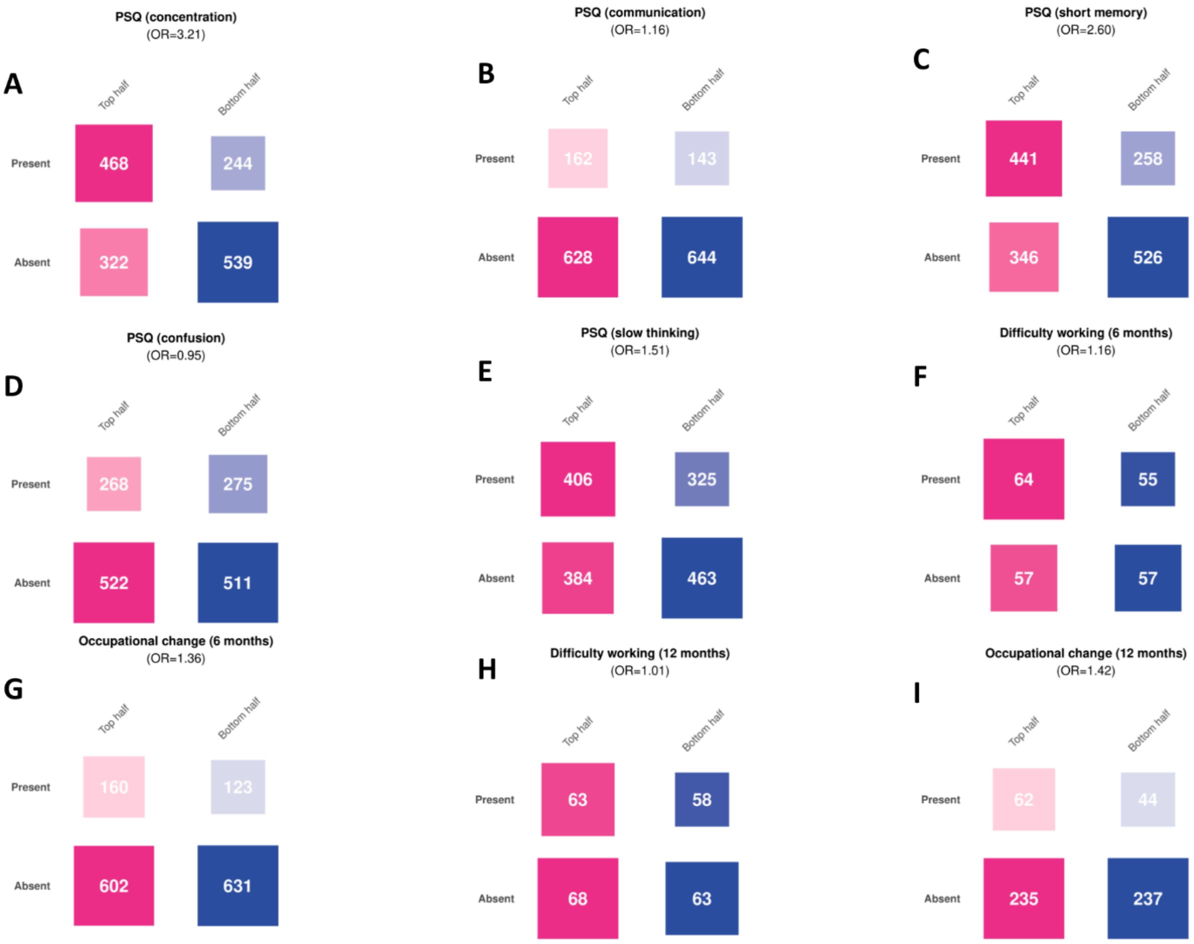

Supplementary Figure 6: Contingency tables showing the distributions of binary variables between the top and bottom half of the cohort along the first dimension of covariation. This is represented for signs of occupational impact (i.e. difficulty working and occupational changes) measured at 6 and 12 months follow-up and the 5 binary items of the C-PSQ score measured at the 6-months follow-up.

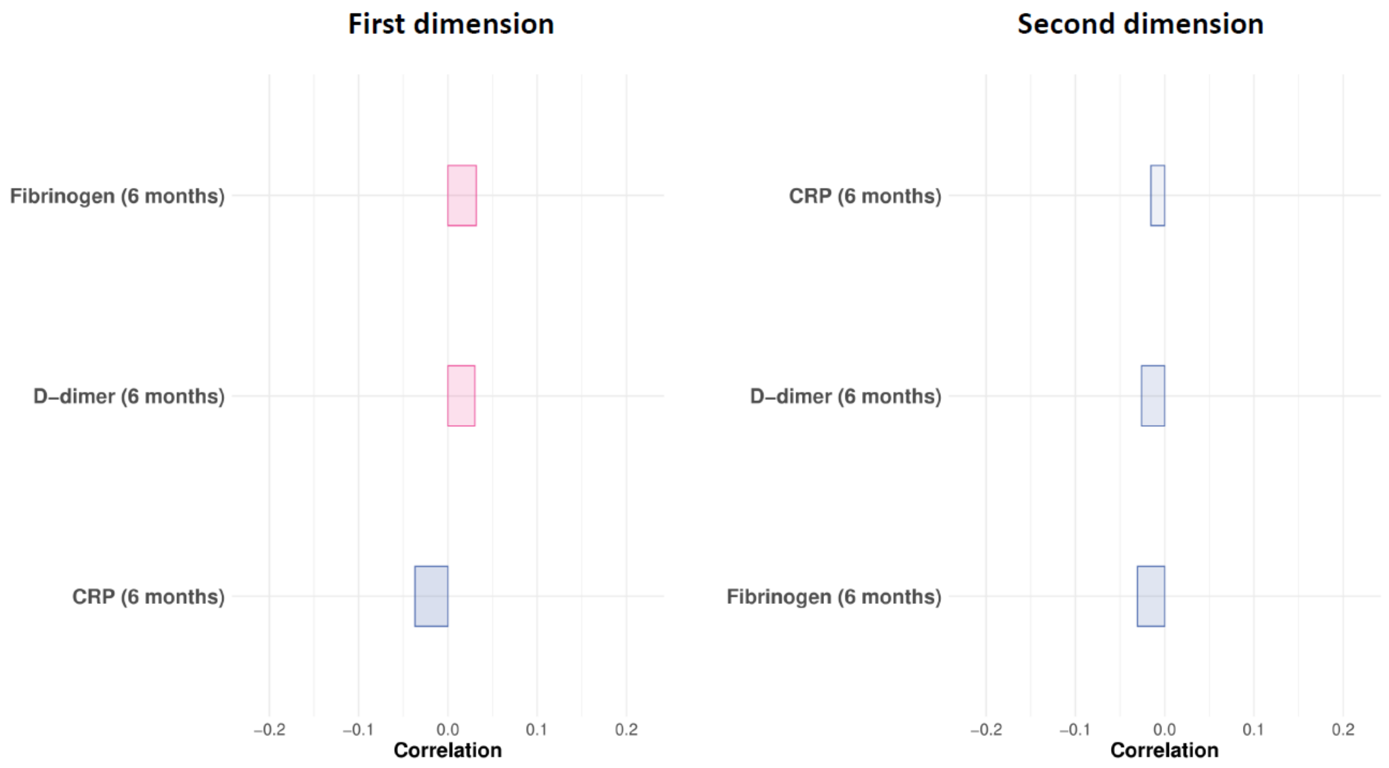

Supplementary Figure 7: Correlations of the key biomarkers measured at the 6-month follow-up with the cognitive profiles of the first and second dimensions of covariation. No association reached statistical significance.

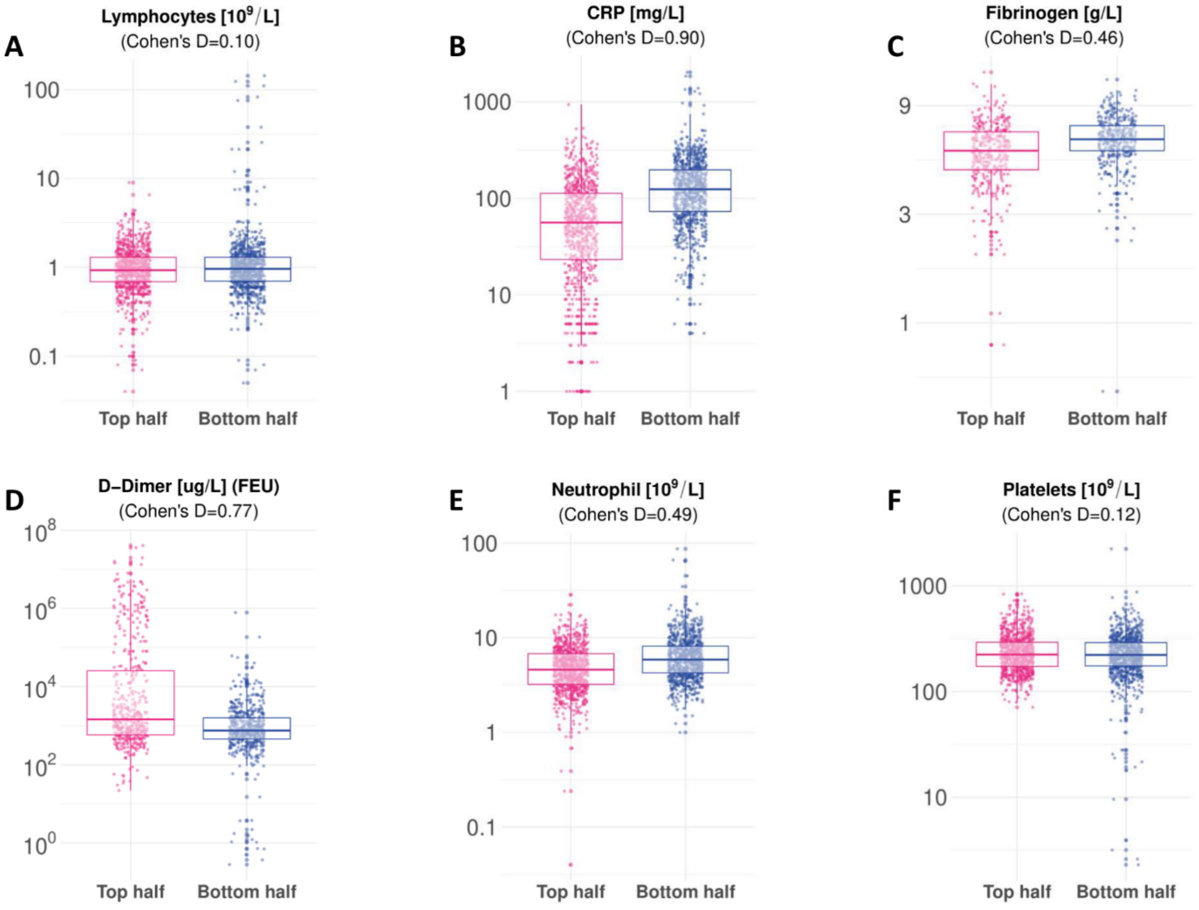

Supplementary Figure 8: Distribution of biomarkers between the top half and the bottom half of the cohort along the second dimension of covariation. Each of the six biomarkers are represented and only complete data are displayed (n=1818, 1777, 768, 977, 1796, 1810 individual participants respectively). The centre of the boxes represent the median, their bounds represent the 25th and 75th centile and the lower and upper ends of whiskers represent the smallest/largest value, no further than 1.5 times the interquartile range from the boxplot respective end.

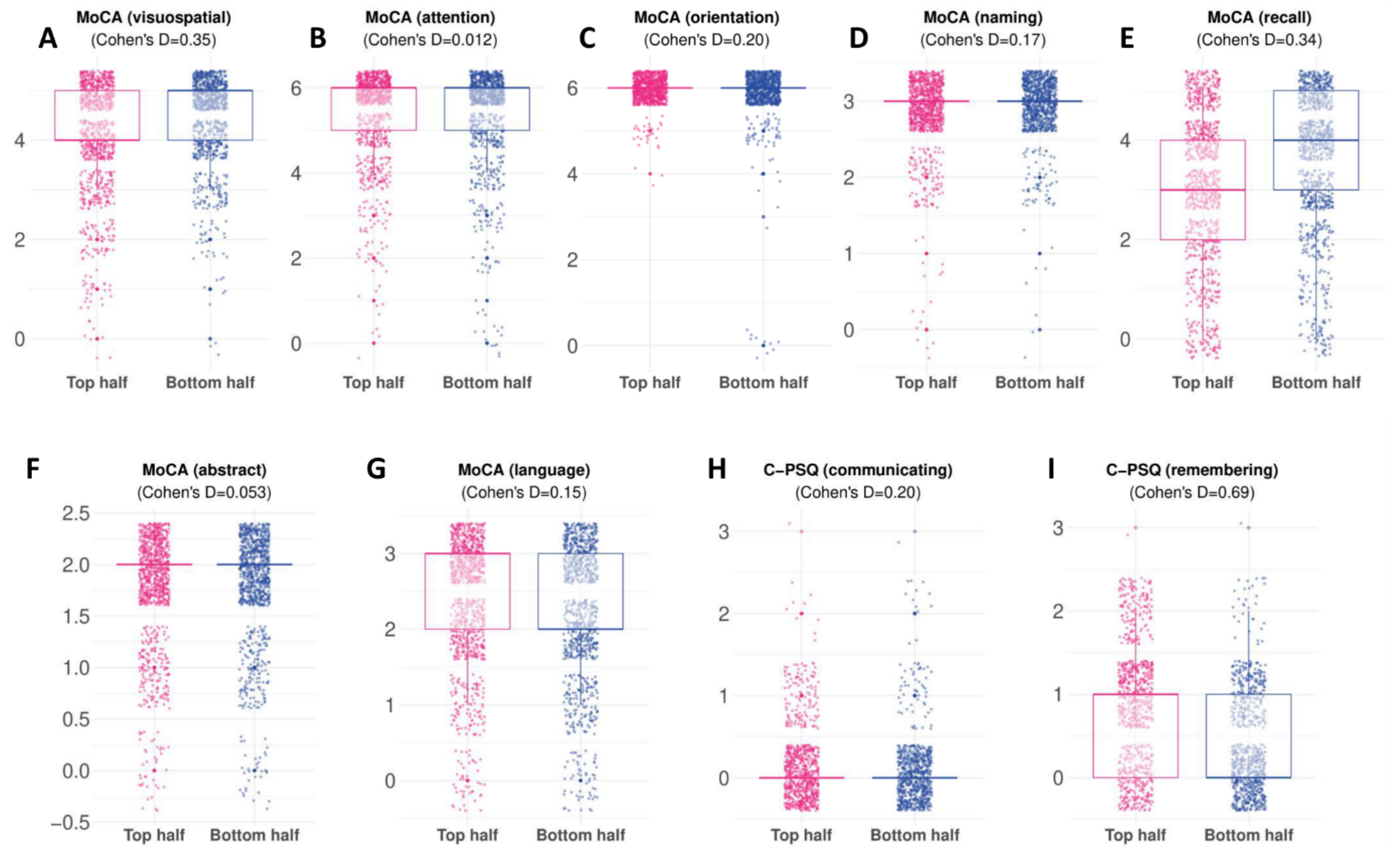

Supplementary Figure 9: Distribution of test scores between the top and the bottom half of the cohort along the second dimension of covariation for different items of the MoCA and the numerical part of the C-PSQ score measured at the 6-months follow-up. Higher C-PSQ scores and lower MoCA scores represent more deficits. Only complete data are displayed (n=1837 individual participants for A-G and 1537, 1544 for H and I, respectively). The centre of the boxes represent the median, their bounds represent the 25th and 75th centile and the lower and upper ends of whiskers represent the smallest/largest value, no further than 1.5 times the interquartile range from the boxplot respective end.

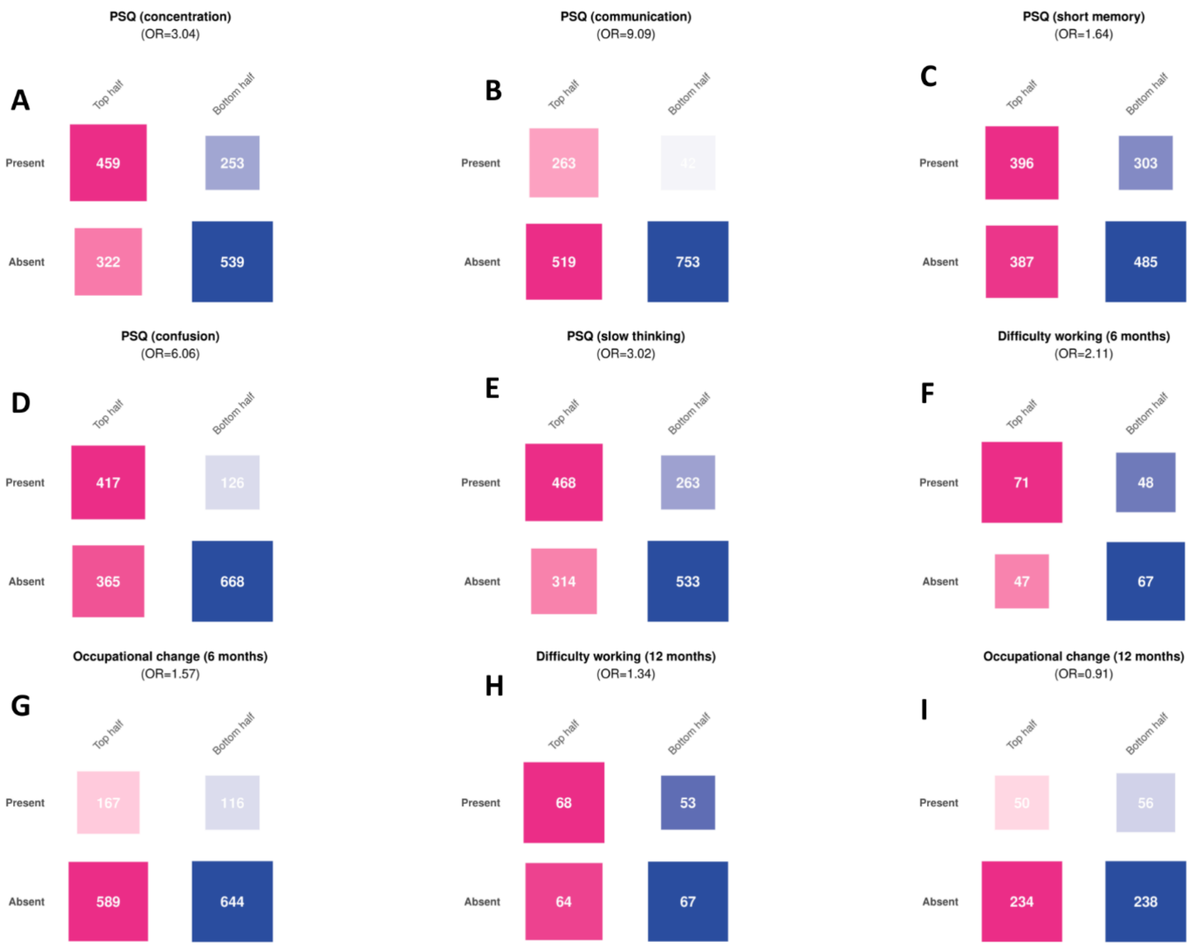

Supplementary Figure 10: Contingency tables showing the distributions of binary variables between the top and bottom halves of the cohort along the second dimension of covariation. This is represented for signs of occupational impact (i.e. difficulty working and occupational changes) measured at 6 and 12 months follow-up and the 5 binary items of the C-PSQ score measured at the 6-months follow-up.

Supplementary Tables

Supplementary Table 1: Baseline characteristics for the entire PHOSP cohort, the study cohort, and the study cohort at the 12 months' follow-up.

**Cohort**	**PHOSP cohort**	**Study cohort with 6-month follow-up**	**12-month follow-up study cohort**
Number	(N=6134)	(N=1837)	(N=626)
DEMOGRAPHICS			
Age; mean (SD)	59.1 (13.5)	57.9 (12.4)	58.7 (12.2)
Sex; n (%)			
Female	3166 (51.6)	1060 (57.7)	385 (61.5)
Male	2130 (34.7)	673 (36.6)	221 (35.3)
Missing	838 (13.7)	104 (5.7)	20 (3.2)
Race; n (%)			
White	4135 (67.4)	1385 (75.4)	487 (77.8)
Mixed	77 (1.3)	26 (1.4)	10 (1.6)
Asian	518 (8.4)	217 (11.8)	79 (12.6)
Black	282 (4.6)	104 (5.7)	33 (5.3)
Other/Unknown	1122 (18.3)	105 (5.7)	17 (2.7)
Education			
None/Primary school	258 (4.2)	90 (4.9)	25 (4.0)
Secondary school	1545 (25.2)	550 (29.9)	185 (29.6)
Sixth form college	618 (10.1)	237 (12.9)	80 (12.8)
Vocational qualification	624 (10.2)	222 (12.1)	81 (12.9)
Undergraduate university degree	763 (12.4)	301 (16.4)	110 (17.6)
Postgraduate qualification	602 (9.8)	237 (12.9)	91 (14.5)
Prefer not to say	428 (7.0)	51 (2.8)	11 (1.8)
Missing	1296 (21.1)	149 (8.1)	43 (6.9)
Income, n (%)			
< £19,000	796 (13.0)	252 (13.7)	77 (12.3)
£19,001 - £26,000	564 (9.2)	207 (11.3)	76 (12.1)
£26,001 - £35,000	498 (8.1)	202 (11.0)	75 (12.0)
£35,001 - £48,000	519 (8.5)	200 (10.9)	69 (11.0)
> £48,001	936 (15.3)	439 (23.9)	159 (25.4)
Prefer not to say	1521 (24.8)	386 (21.0)	129 (20.6)
Missing	1300 (21.2)	151 (8.2)	41 (6.5)
Is Married, n (%)			
Yes	2870 (46.8)	1034 (56.3)	371 (59.3)
No	2083 (34.0)	669 (36.4)	217 (34.7)
Missing	1181 (19.3)	134 (7.3)	38 (6.1)
English as the first language, n (%)			
Yes	4152 (67.7)	1469 (80.0)	507 (81.0)
No	828 (13.5)	244 (13.3)	86 (13.7)
Missing	1154 (18.8)	124 (6.8)	33 (5.3)
COMORBIDITIES, n (%)			
Cardiovascular condition	2553 (41.6)	826 (45.0)	289 (46.2)
Cerebrovascular accident	215 (3.5)	79 (4.3)	27 (4.3)
Dementia	17 (0.3)	< 10	< 10

Parkinson's disease	< 10	< 10	< 10
Neurological or psychiatric condition	971 (15.8)	332 (18.1)	100 (16.0)
Chronic condition	242 (3.9)	93 (5.1)	29 (4.6)
Diabetes	1139 (18.6)	366 (19.9)	133 (21.2)
Respiratory condition	1575 (25.7)	507 (27.6)	178 (28.4)
Rheumatological condition	854 (13.9)	285 (15.5)	97 (15.5)
Gastrointestinal condition	1044 (17.0)	391 (21.3)	131 (20.9)
Endocrine condition	471 (7.7)	147 (8.0)	49 (7.8)
Chronic kidney disease	272 (4.4)	72 (3.9)	22 (3.5)
Cancer	400 (6.5)	134 (7.3)	49 (7.8)
Infectious disease	145 (2.4)	38 (2.1)	13 (2.1)

Supplementary Table 2: Weights of the weighted combination representing the biomarker profile of the first dimension of covariation, relative weights (representing the correlation of each biomarker with the biomarker profile), and two-sided p-values (not adjusted for multiple comparisons) for each relative weight calculated using a permutation test. Note that the minimum p-value achievable with a permutation test with 10,000 permutations is 0.0001. Some associations might have smaller p-values and would be reported as p=0.0001.

	**Weights**	**Relative weight**	**P-value**
D-dimer	0.052	0.079	0.0010
Neutrophils	-0.14	-0.11	0.0003
Platelets	-0.42	-0.14	0.0001
CRP	-0.78	0.032	0.17
Fibrinogen	1.36	0.69	0.0001
Lymphocytes	0.057	0.052	0.025

Supplementary Table 3: Correlations and p-values (derived from permutation tests, two-sided, and not adjusted for multiple comparisons) between cognitive outcomes (including cognitive scores and signs of occupational impact at 6 and 12 months) and the cognitive profile of the first dimension (i.e. what is displayed in Fig. 2B of the main manuscript). The correlations and p-values are also provided for the association between cognitive outcomes and the dimension of covariation (i.e. the location of individuals along the dimension of covariation which is calculated as the mean between the cognitive and biomarker profile). Two-sided p-values (not adjusted for multiple comparisons) for each correlation were calculated using a permutation test. Note that the minimum p-value achievable with a permutation test with 10,000 permutations is 0.0001. Some associations might have smaller p-values and would be reported as p=0.0001

	Correlation with the cognitive profile	P value (Cognitive profile)	Correlation with the dimension of covariation	P value (Dimension of covariation)
MoCA (6 months)	-0.075	0.0012	-0.057	0.014
MoCA (12 months)	-0.16	< 0.0001	-0.12	0.0023
Occupational change (6 months)	0.044	0.091	0.044	0.087
Occupational change (12 months)	0.077	0.067	0.12	0.0024
Difficulty working (6 months)	0.086	0.19	0.087	0.18
Difficulty working (12 months)	0.054	0.4	0.049	0.44
C-PSQ (6 months)	0.24	< 0.0001	0.19	0.0001
C-PSQ (12 months)	0.11	0.0061	0.058	0.15

Supplementary Table 4: Weights of the weighted combination representing the biomarker profile of the second dimension of covariation, relative weights (representing the correlation of each biomarker with the biomarker profile), and two-sided p-values for each relative weight calculated using a permutation test (two-sided and non-adjusted for multiple comparisons). Note that the minimum p-value achievable with a permutation test with 10,000 permutations is 0.0001. Some associations might have smaller p-values and would be reported as p=0.0001

	**Weight**	**Relative weight**	**P-value**
D-dimer	0.68	0.51	0.0001
Neutrophils	-0.29	-0.36	0.0001
Platelets	0.3	0.16	0.0001
CRP	-0.76	-0.62	0.0001
Fibrinogen	-0.007	-0.35	0.0001
Lymphocytes	-0.26	-0.13	0.0001

Supplementary Table 5: Correlations and p-values (derived from permutation tests, two-sided, and not adjusted for multiple comparisons) between cognitive outcomes (including cognitive scores and signs of occupational impact at 6 and 12 months) and the cognitive profile of the second dimension of covariation (i.e. what is displayed in Fig. 3B of the main manuscript). The correlations and p-values are also provided for the association between cognitive outcomes and the dimension of covariation (i.e. the location of individuals along the dimension of covariation which is calculated as the mean between the cognitive and biomarker profile). Two-sided p-values (not adjusted for multiple comparisons) for each correlation were calculated using a permutation test. Note that the minimum p-value achievable with a permutation test with 10,000 permutations is 0.0001. Some associations might have smaller p-values and would be reported as p=0.0001

	Correlation with the cognitive profile	P value (Cognitive profile)	Correlation with the dimension of covariation	P value (Dimension of covariation)
MoCA (6 months)	-0.039	0.095	-0.03	0.19
MoCA (12 months)	-0.078	0.051	-0.053	0.18
Occupational change (6 months)	0.12	< 0.0001	0.096	0.0002
Occupational change (12 months)	0.074	0.079	0.018	0.66
Difficulty working (6 months)	0.24	0.0003	0.24	0.0003
Difficulty working (12 months)	0.19	0.0018	0.097	0.12
C-PSQ (6 months)	0.53	< 0.0001	0.41	0.0001
C-PSQ (12 months)	0.35	< 0.0001	0.25	0.0001

Supplementary Table 6: Results of the mediation analysis wherein the biomarker profile was the independent variable, the cognitive profile was the dependent variable, and mediators were 14 different clinical scales capturing different aspect of participants' health. Specifically, the table presents fractions explained by each mediator for the link between biomarker and cognitive profile alongside the 1st and 2nd mode of covariation, as well as the two-sided p-value testing the null hypothesis of no mediation. P-values were two-sided, not adjusted for multiple comparisons, and derived from non-parametric bootstrap with 1000 bootstrap repetitions.

	Fraction explained [%] (first dimension)	P value (first dimension)	Fraction explained [%] (second dimension)	P value (second dimension)
PHQ-9	1.21	0.31	5.18	0.098
GAD-7	0.92	0.35	1.49	0.65
PCL-5	0.4	0.68	4.31	0.25
Dyspnoea-12	0.3	0.73	8.63	<0.001
GPPAQ occupational	-0.25	0.58	0.082	0.9
GPPAQ exercise	-0.25	0.61	0.061	0.92
GPPAQ PAI	-0.54	0.4	0.13	0.76
SARC-F	0.15	0.87	4.16	0.054
FACIT	0.23	0.82	9.05	0.004
SPPB	0.78	0.22	-0.41	0.72
ISWT	0.089	0.81	0.57	0.7
BPI severity	0.55	0.5	1.6	0.32
BPI interference	0.76	0.4	3.72	0.086
ISWT relative	0.38	0.53	0.28	0.87

Supplementary Table 7: Baseline characteristics from the EHR dataset in the cohorts hospitalised with COVID-19, and with high and low fibrinogen (and normal CRP), before and after propensity score matching.

	**Hospitalised with COVID-19 high fibrinogen (Unmatched)**	**Hospitalised with COVID-19 low fibrinogen (Unmatched)**	**Hospitalised with COVID-19 high fibrinogen (Matched)**	**Hospitalised with COVID-19 low fibrinogen (Matched)**
Total Number	1404	2542	1276	1276
DEMOGRAPHICS				
Age; mean (SD)	48.8 (19.5)	44.5 (24.2)	48.1 (19.9)	48.7 (22.3)
Sex; n (%)				
Female	480 (34.2)	1259 (49.5)	474 (37.1)	488 (38.2)
Male	920 (65.5)	1283 (50.5)	802 (62.9)	788 (61.8)
Other	10 (0.7)	0 (0.0)	0 (0.0)	0 (0.0)
Race; n (%)				
White	607 (43.2)	1234 (48.5)	572 (44.8)	576 (45.1)
Black or African American	415 (29.6)	650 (25.6)	372 (29.2)	375 (29.4)
Asian	41 (2.9)	80 (3.1)	36 (2.8)	37 (2.9)
American Indian or Alaska Native	10 (0.7)	18 (0.7)	10 (0.8)	10 (0.8)
Native Hawaiian or Other Pacific Islander	10 (0.7)	10 (0.4)	10 (0.8)	10 (0.8)
Unknown	327 (23.3)	557 (21.9)	285 (22.3)	278 (21.8)
Ethnicity; n (%)				
Hispanic or Latino	361 (25.7)	430 (16.9)	294 (23.0)	307 (24.1)
Not Hispanic or Latino	923 (65.7)	1859 (73.1)	871 (68.3)	858 (67.2)
Unknown	120 (8.5)	253 (10.0)	111 (8.7)	111 (8.7)
Problems related to housing and economic circumstances	12 (0.9)	60 (2.4)	12 (0.9)	10 (0.8)
COMORBIDITIES				
Overweight and obesity	378 (26.9)	617 (24.3)	336 (26.3)	345 (27.0)
Hypertensive disease; n (%)				
Hypertensive diseases	494 (35.2)	921 (36.2)	451 (35.3)	454 (35.6)
Hypertensive diseases (deprecated 2018)	493 (35.1)	918 (36.1)	450 (35.3)	453 (35.5)
Diabetes mellitus; n (%)				
Type 1 diabetes mellitus	21 (1.5)	37 (1.5)	19 (1.5)	19 (1.5)
Type 2 diabetes mellitus	331 (23.6)	518 (20.4)	292 (22.9)	298 (23.4)
Chronic lower respiratory diseases; n (%)				
Asthma	127 (9.0)	264 (10.4)	116 (9.1)	110 (8.6)
Bronchiectasis	10 (0.7)	12 (0.5)	10 (0.8)	10 (0.8)
Bronchitis, not specified as acute or chronic	27 (1.9)	55 (2.2)	24 (1.9)	22 (1.7)
Emphysema	24 (1.7)	32 (1.3)	17 (1.3)	20 (1.6)
Other chronic obstructive pulmonary disease	58 (4.1)	134 (5.3)	54 (4.2)	54 (4.2)
Simple and mucopurulent chronic bronchitis	10 (0.7)	10 (0.4)	10 (0.8)	10 (0.8)
Unspecified chronic bronchitis	10 (0.7)	11 (0.4)	10 (0.8)	10 (0.8)
Nicotine dependence	111 (7.9)	248 (9.8)	101 (7.9)	105 (8.2)
Psychiatric comorbidities; n (%)				
Anxiety disorders	185 (13.2)	440 (17.3)	175 (13.7)	172 (13.5)
Substance misuse	164 (11.7)	373 (14.7)	149 (11.7)	149 (11.7)
Mood disorders	148 (10.5)	333 (13.1)	135 (10.6)	136 (10.7)
Psychotic disorders	28 (2.0)	64 (2.5)	23 (1.8)	21 (1.6)
Heart disease; n (%)				
Ischemic heart diseases	144 (10.3)	301 (11.8)	133 (10.4)	150 (11.8)
Other forms of heart disease	343 (24.4)	712 (28.0)	317 (24.8)	297 (23.3)
Chronic kidney diseases; n (%)				
Chronic kidney disease (CKD)	95 (6.8)	206 (8.1)	88 (6.9)	89 (7.0)

Hypertensive chronic kidney disease	61 (4.3)	107 (4.2)	54 (4.2)	60 (4.7)
Chronic liver diseases; n (%)				
Alcoholic liver disease	10 (0.7)	18 (0.7)	10 (0.8)	10 (0.8)
Chronic hepatitis, not elsewhere classified	10 (0.7)	10 (0.4)	10 (0.8)	10 (0.8)
Chronic passive congestion of liver	10 (0.7)	10 (0.4)	10 (0.8)	10 (0.8)
Fatty (change of) liver, not elsewhere classified	40 (2.8)	84 (3.3)	37 (2.9)	32 (2.5)
Fibrosis and cirrhosis of liver	10 (0.7)	50 (2.0)	10 (0.8)	10 (0.8)
Hepatic failure, not elsewhere classified	22 (1.6)	55 (2.2)	18 (1.4)	21 (1.6)
Other specified diseases of liver	17 (1.2)	40 (1.6)	17 (1.3)	16 (1.3)
Portal hypertension	10 (0.7)	26 (1.0)	10 (0.8)	10 (0.8)
Cerebral infarction	44 (3.1)	156 (6.1)	44 (3.4)	51 (4.0)
Dementia; n (%)				
Alzheimer's disease	10 (0.7)	20 (0.8)	10 (0.8)	10 (0.8)
Dementia in other diseases classified elsewhere	10 (0.7)	23 (0.9)	10 (0.8)	10 (0.8)
Dementia with Lewy bodies	0 (0.0)	10 (0.4)	0 (0.0)	0 (0.0)
Frontotemporal dementia	10 (0.7)	0 (0.0)	0 (0.0)	0 (0.0)
Unspecified dementia	15 (1.1)	59 (2.3)	15 (1.2)	18 (1.4)
Vascular dementia	10 (0.7)	10 (0.4)	10 (0.8)	10 (0.8)
Neoplasms; n (%)				
Malignant neoplasms of lymphoid, hematopoietic and related tissue	16 (1.1)	75 (3.0)	16 (1.3)	19 (1.5)
Neoplasms (any including benign)	151 (10.8)	380 (14.9)	145 (11.4)	137 (10.7)
Organ transplant; n (%)				
Liver Transplantation Procedures	0 (0.0)	10 (0.4)	0 (0.0)	0 (0.0)
Renal Transplantation Procedures	10 (0.7)	10 (0.4)	10 (0.8)	10 (0.8)
Psoriasis	12 (0.9)	20 (0.8)	10 (0.8)	10 (0.8)
Rheumatoid arthritis; n (%)				
Other rheumatoid arthritis	21 (1.5)	29 (1.1)	17 (1.3)	18 (1.4)
Rheumatoid arthritis with rheumatoid factor	10 (0.7)	10 (0.4)	10 (0.8)	10 (0.8)
Systemic lupus erythematosus (SLE)	10 (0.7)	13 (0.5)	10 (0.8)	10 (0.8)
Disorders involving the immune mechanism	40 (2.8)	115 (4.5)	35 (2.7)	35 (2.7)
MEDICATIONS				
COVID-19 vaccine; n (%)				
Pfizer	10 (0.7)	10 (0.4)	10 (0.8)	10 (0.8)
Moderna	0 (0.0)	10 (0.4)	0 (0.0)	0 (0.0)
Janssen	0 (0.0)	0 (0.0)	0 (0.0)	0 (0.0)
Not specified	10 (0.7)	37 (1.5)	10 (0.8)	10 (0.8)
Antidepressants; n (%)				
Any	168 (12.0)	393 (15.5)	158 (12.4)	158 (12.4)
Fluvoxamine	10 (0.7)	0 (0.0)	0 (0.0)	0 (0.0)
Lithium	10 (0.7)	10 (0.4)	10 (0.8)	10 (0.8)
Antipsychotics; n (%)				
Any	63 (4.5)	151 (5.9)	57 (4.5)	58 (4.5)
Clozapine	0 (0.0)	10 (0.4)	0 (0.0)	0 (0.0)

Supplementary Table 8: Baseline characteristics from the EHR dataset in the cohorts hospitalised with COVID-19, and with high and low D-dimer, before and after propensity score matching.

	**Hospitalised with COVID-19 High D-dimer (Unmatched)**	**Hospitalised with COVID-19 Low D-dimer (Unmatched)**	**Hospitalised with COVID-19 High D-dimer (Matched)**	**Hospitalised with COVID-19 Low D-dimer (Matched)**
Total Number	8207	6330	5722	5722
DEMOGRAPHICS				
Age; mean (SD)	54.7 (19.6)	52.5 (17.4)	52.7 (19.6)	53.0 (17.4)
Sex; n (%)				
Female	3583 (43.7)	3054 (48.2)	2610 (45.6)	2645 (46.2)
Male	4614 (56.2)	3276 (51.8)	3112 (54.4)	3077 (53.8)
Other	10 (0.1)	0 (0.0)	0 (0.0)	0 (0.0)
Race; n (%)				
White	4446 (54.2)	4252 (67.2)	3704 (64.7)	3709 (64.8)
Black or African American	1948 (23.7)	1107 (17.5)	1024 (17.9)	1071 (18.7)
Asian	398 (4.8)	190 (3.0)	215 (3.8)	188 (3.3)
American Indian or Alaska Native	49 (0.6)	50 (0.8)	43 (0.8)	38 (0.7)
Native Hawaiian or Other Pacific Islander	22 (0.3)	19 (0.3)	19 (0.3)	18 (0.3)
Unknown	1344 (16.4)	712 (11.2)	717 (12.5)	698 (12.2)
Ethnicity; n (%)				
Hispanic or Latino	1805 (22.0)	1348 (21.3)	1264 (22.1)	1251 (21.9)
Not Hispanic or Latino	6084 (74.1)	4739 (74.9)	4231 (73.9)	4255 (74.4)
Unknown	318 (3.9)	243 (3.8)	227 (4.0)	216 (3.8)
Problems related to housing and economic circumstances	75 (0.9)	94 (1.5)	66 (1.2)	60 (1.0)
COMORBIDITIES				
Overweight and obesity	1766 (21.5)	1770 (28.0)	1438 (25.1)	1426 (24.9)
Hypertensive disease; n (%)				
Hypertensive diseases	3120 (38.0)	2431 (38.4)	2145 (37.5)	2127 (37.2)
Hypertensive diseases (deprecated 2018)	3105 (37.8)	2422 (38.3)	2136 (37.3)	2120 (37.0)
Diabetes mellitus; n (%)				
Type 1 diabetes mellitus	111 (1.4)	94 (1.5)	74 (1.3)	83 (1.5)
Type 2 diabetes mellitus	1900 (23.2)	1408 (22.2)	1245 (21.8)	1246 (21.8)
Chronic lower respiratory diseases; n (%)				
Asthma	586 (7.1)	656 (10.4)	498 (8.7)	514 (9.0)
Bronchiectasis	40 (0.5)	18 (0.3)	17 (0.3)	18 (0.3)
Bronchitis, not specified as acute or chronic	157 (1.9)	218 (3.4)	144 (2.5)	151 (2.6)
Emphysema	148 (1.8)	108 (1.7)	89 (1.6)	97 (1.7)
Other chronic obstructive pulmonary disease	448 (5.5)	396 (6.3)	326 (5.7)	337 (5.9)
Simple and mucopurulent chronic bronchitis	25 (0.3)	26 (0.4)	19 (0.3)	18 (0.3)
Unspecified chronic bronchitis	29 (0.4)	33 (0.5)	21 (0.4)	24 (0.4)
Nicotine dependence	549 (6.7)	518 (8.2)	418 (7.3)	418 (7.3)
Psychiatric comorbidities; n (%)				
Anxiety disorders	1020 (12.4)	1091 (17.2)	818 (14.3)	832 (14.5)
Substance misuse	825 (10.1)	721 (11.4)	592 (10.3)	589 (10.3)
Mood disorders	816 (9.9)	879 (13.9)	653 (11.4)	663 (11.6)
Psychotic disorders	145 (1.8)	119 (1.9)	100 (1.7)	99 (1.7)
Heart disease; n (%)				
Ischemic heart diseases	945 (11.5)	676 (10.7)	607 (10.6)	593 (10.4)
Other forms of heart disease	2101 (25.6)	1619 (25.6)	1422 (24.9)	1421 (24.8)
Chronic kidney diseases; n (%)				
Chronic kidney disease (CKD)	702 (8.6)	531 (8.4)	446 (7.8)	437 (7.6)

Hypertensive chronic kidney disease	311 (3.8)	155 (2.4)	157 (2.7)	148 (2.6)
Chronic liver diseases; n (%)				
Alcoholic liver disease	53 (0.6)	10 (0.2)	10 (0.2)	10 (0.2)
Chronic hepatitis, not elsewhere classified	10 (0.1)	10 (0.2)	10 (0.2)	10 (0.2)
Chronic passive congestion of liver	29 (0.4)	16 (0.3)	14 (0.2)	15 (0.3)
Fatty (change of) liver, not elsewhere classified	248 (3.0)	289 (4.6)	204 (3.6)	215 (3.8)
Fibrosis and cirrhosis of liver	84 (1.0)	61 (1.0)	51 (0.9)	46 (0.8)
Hepatic failure, not elsewhere classified	69 (0.8)	13 (0.2)	10 (0.2)	13 (0.2)
Other specified diseases of liver	128 (1.6)	121 (1.9)	96 (1.7)	84 (1.5)
Portal hypertension	34 (0.4)	10 (0.2)	10 (0.2)	10 (0.2)
Cerebral infarction	233 (2.8)	173 (2.7)	151 (2.6)	140 (2.4)
Dementia; n (%)				
Alzheimer's disease	67 (0.8)	36 (0.6)	28 (0.5)	34 (0.6)
Dementia in other diseases classified elsewhere	84 (1.0)	46 (0.7)	42 (0.7)	42 (0.7)
Dementia with Lewy bodies	10 (0.1)	10 (0.2)	10 (0.2)	10 (0.2)
Frontotemporal dementia	10 (0.1)	0 (0.0)	0 (0.0)	0 (0.0)
Unspecified dementia	191 (2.3)	101 (1.6)	93 (1.6)	94 (1.6)
Vascular dementia	22 (0.3)	22 (0.3)	18 (0.3)	14 (0.2)
Neoplasms; n (%)				
Malignant neoplasms of lymphoid, hematopoietic and related tissue	75 (0.9)	48 (0.8)	48 (0.8)	43 (0.8)
Neoplasms (any including benign)	988 (12.0)	904 (14.3)	724 (12.7)	724 (12.7)
Organ transplant; n (%)				
Liver Transplantation Procedures	10 (0.1)	0 (0.0)	0 (0.0)	0 (0.0)
Renal Transplantation Procedures	10 (0.1)	0 (0.0)	0 (0.0)	0 (0.0)
Psoriasis	58 (0.7)	46 (0.7)	42 (0.7)	41 (0.7)
Rheumatoid arthritis; n (%)				
Other rheumatoid arthritis	104 (1.3)	86 (1.4)	63 (1.1)	71 (1.2)
Rheumatoid arthritis with rheumatoid factor	21 (0.3)	20 (0.3)	13 (0.2)	17 (0.3)
Systemic lupus erythematosus (SLE)	42 (0.5)	31 (0.5)	24 (0.4)	27 (0.5)
Disorders involving the immune mechanism	198 (2.4)	250 (3.9)	164 (2.9)	183 (3.2)
MEDICATIONS				
COVID-19 vaccine; n (%)				
Pfizer	50 (0.6)	53 (0.8)	37 (0.6)	39 (0.7)
Moderna	10 (0.1)	10 (0.2)	10 (0.2)	10 (0.2)
Janssen	10 (0.1)	10 (0.2)	10 (0.2)	10 (0.2)
Not specified	135 (1.6)	59 (0.9)	49 (0.9)	53 (0.9)
Antidepressants; n (%)				
Any	1151 (14.0)	1187 (18.8)	935 (16.3)	938 (16.4)
Fluvoxamine	13 (0.2)	10 (0.2)	10 (0.2)	10 (0.2)
Lithium	14 (0.2)	10 (0.2)	10 (0.2)	10 (0.2)
Antipsychotics; n (%)				
Any	425 (5.2)	348 (5.5)	284 (5.0)	301 (5.3)
Clozapine	10 (0.1)	10 (0.2)	10 (0.2)	10 (0.2)

Supplementary Table 9: Baseline characteristics from the EHR dataset in the cohorts hospitalised before the COVID-19 pandemic (i.e. without COVID-19), and with high and low fibrinogen, before and after propensity score matching.

	**Other hospitalisation high fibrinogen (Unmatched)**	**Other hospitalisation low fibrinogen (Unmatched)**	**Other hospitalisation high fibrinogen (Matched)**	**Other hospitalisation low fibrinogen (Matched)**
Total Number	6786	34139	6782	6782
DEMOGRAPHICS				
Age; mean (SD)	40.4 (22.5)	40.6 (25.1)	40.4 (22.5)	41.2 (23.9)
Sex; n (%)				
Female	3124 (46.0)	16417 (48.1)	3123 (46.0)	3144 (46.4)
Male	3652 (53.8)	17677 (51.8)	3649 (53.8)	3629 (53.5)
Other	10 (0.1)	45 (0.1)	10 (0.1)	10 (0.1)
Race; n (%)				
White	3864 (56.9)	21263 (62.3)	3864 (57.0)	3898 (57.5)
Black or African American	1682 (24.8)	6517 (19.1)	1678 (24.7)	1670 (24.6)
Asian	159 (2.3)	1016 (3.0)	159 (2.3)	157 (2.3)
American Indian or Alaska Native	75 (1.1)	349 (1.0)	75 (1.1)	81 (1.2)
Native Hawaiian or Other Pacific Islander	18 (0.3)	72 (0.2)	18 (0.3)	18 (0.3)
Unknown	988 (14.6)	4922 (14.4)	988 (14.6)	958 (14.1)
Ethnicity; n (%)				
Hispanic or Latino	849 (12.5)	4304 (12.6)	848 (12.5)	866 (12.8)
Not Hispanic or Latino	5235 (77.1)	25663 (75.2)	5232 (77.1)	5256 (77.5)
Unknown	702 (10.3)	4172 (12.2)	702 (10.4)	660 (9.7)
Problems related to housing and economic circumstances	92 (1.4)	444 (1.3)	92 (1.4)	91 (1.3)
COMORBIDITIES				
Overweight and obesity	1420 (20.9)	5142 (15.1)	1418 (20.9)	1436 (21.2)
Hypertensive disease; n (%)				
Hypertensive diseases	3270 (48.2)	14217 (41.6)	3266 (48.2)	3242 (47.8)
Hypertensive diseases (deprecated 2018)	3265 (48.1)	14189 (41.6)	3261 (48.1)	3238 (47.7)
Diabetes mellitus; n (%)				
Type 1 diabetes mellitus	413 (6.1)	1343 (3.9)	411 (6.1)	410 (6.0)
Type 2 diabetes mellitus	1579 (23.3)	6377 (18.7)	1575 (23.2)	1574 (23.2)
Chronic lower respiratory diseases; n (%)				
Asthma	795 (11.7)	3348 (9.8)	794 (11.7)	783 (11.5)
Bronchiectasis	111 (1.6)	367 (1.1)	109 (1.6)	104 (1.5)
Bronchitis, not specified as acute or chronic	286 (4.2)	1066 (3.1)	284 (4.2)	273 (4.0)
Emphysema	233 (3.4)	851 (2.5)	231 (3.4)	246 (3.6)
Other chronic obstructive pulmonary disease	567 (8.4)	2538 (7.4)	564 (8.3)	558 (8.2)
Simple and mucopurulent chronic bronchitis	42 (0.6)	147 (0.4)	42 (0.6)	48 (0.7)
Unspecified chronic bronchitis	56 (0.8)	203 (0.6)	55 (0.8)	51 (0.8)
Nicotine dependence	1149 (16.9)	5062 (14.8)	1147 (16.9)	1130 (16.7)
Psychiatric comorbidities; n (%)				
Anxiety disorders	1738 (25.6)	6872 (20.1)	1734 (25.6)	1706 (25.2)
Substance misuse	1725 (25.4)	7734 (22.7)	1722 (25.4)	1669 (24.6)
Mood disorders	1523 (22.4)	6381 (18.7)	1521 (22.4)	1515 (22.3)
Psychotic disorders	240 (3.5)	1047 (3.1)	240 (3.5)	237 (3.5)
Heart disease; n (%)				
Ischemic heart diseases	1513 (22.3)	6846 (20.1)	1510 (22.3)	1498 (22.1)
Other forms of heart disease	3336 (49.2)	14972 (43.9)	3332 (49.1)	3233 (47.7)

Chronic kidney diseases; n (%)				
Chronic kidney disease (CKD)	1316 (19.4)	5004 (14.7)	1313 (19.4)	1308 (19.3)
Hypertensive chronic kidney disease	867 (12.8)	3084 (9.0)	865 (12.8)	861 (12.7)
Chronic liver diseases; n (%)				
Alcoholic liver disease	66 (1.0)	977 (2.9)	66 (1.0)	61 (0.9)
Chronic hepatitis, not elsewhere classified	28 (0.4)	177 (0.5)	28 (0.4)	30 (0.4)
Chronic passive congestion of liver	195 (2.9)	904 (2.6)	194 (2.9)	196 (2.9)
Fatty (change of) liver, not elsewhere classified	344 (5.1)	1479 (4.3)	344 (5.1)	335 (4.9)
Fibrosis and cirrhosis of liver	160 (2.4)	1887 (5.5)	160 (2.4)	151 (2.2)
Hepatic failure, not elsewhere classified	343 (5.1)	2042 (6.0)	342 (5.0)	299 (4.4)
Other specified diseases of liver	447 (6.6)	2107 (6.2)	447 (6.6)	447 (6.6)
Portal hypertension	83 (1.2)	1308 (3.8)	83 (1.2)	74 (1.1)
Cerebral infarction	723 (10.7)	4293 (12.6)	723 (10.7)	666 (9.8)
Dementia; n (%)				
Alzheimer's disease	14 (0.2)	77 (0.2)	14 (0.2)	16 (0.2)
Dementia in other diseases classified elsewhere	30 (0.4)	127 (0.4)	30 (0.4)	28 (0.4)
Dementia with Lewy bodies	10 (0.1)	21 (0.06)	10 (0.1)	10 (0.1)
Frontotemporal dementia	10 (0.1)	10 (0.03)	10 (0.1)	10 (0.1)
Unspecified dementia	67 (1.0)	354 (1.0)	67 (1.0)	65 (1.0)
Vascular dementia	26 (0.4)	98 (0.3)	26 (0.4)	34 (0.5)
Neoplasms; n (%)				
Malignant neoplasms of lymphoid, hematopoietic and related tissue	1064 (15.7)	3103 (9.1)	1062 (15.7)	1060 (15.6)
Neoplasms (any including benign)	2363 (34.8)	9496 (27.8)	2360 (34.8)	2297 (33.9)
Organ transplant; n (%)				
Liver Transplantation Procedures	23 (0.3)	251 (0.7)	23 (0.3)	21 (0.3)
Renal Transplantation Procedures	34 (0.5)	230 (0.7)	34 (0.5)	31 (0.5)
Psoriasis	90 (1.3)	376 (1.1)	90 (1.3)	95 (1.4)
Rheumatoid arthritis; n (%)				
Other rheumatoid arthritis	219 (3.2)	1012 (3.0)	219 (3.2)	237 (3.5)
Rheumatoid arthritis with rheumatoid factor	40 (0.6)	136 (0.4)	40 (0.6)	44 (0.6)
Systemic lupus erythematosus (SLE)	269 (4.0)	1205 (3.5)	269 (4.0)	282 (4.2)
Disorders involving the immune mechanism	862 (12.7)	2915 (8.5)	859 (12.7)	826 (12.2)
MEDICATIONS				
COVID-19 vaccine; n (%)				
Pfizer	0 (0.0)	0 (0.0)	0 (0.0)	0 (0.0)
Moderna	0 (0.0)	0 (0.0)	0 (0.0)	0 (0.0)
Janssen	0 (0.0)	0 (0.0)	0 (0.0)	0 (0.0)
Not specified	0 (0.0)	0 (0.0)	0 (0.0)	0 (0.0)
Antidepressants; n (%)				
Any	2472 (36.4)	10308 (30.2)	2468 (36.4)	2537 (37.4)
Fluvoxamine	10 (0.1)	35 (0.1)	10 (0.1)	10 (0.1)
Lithium	23 (0.3)	124 (0.4)	23 (0.3)	26 (0.4)
Antipsychotics; n (%)				
Any	1354 (20.0)	4624 (13.5)	1351 (19.9)	1349 (19.9)
Clozapine	10 (0.1)	19 (0.06)	10 (0.1)	10 (0.1)

Supplementary Table 10: Baseline characteristics from the EHR dataset in the cohorts hospitalised before the COVID-19 pandemic (i.e. without COVID-19), and with high and low D-dimer, before and after propensity score matching.

	**Other hospitalisation High D-dimer (Unmatched)**	**Other hospitalisation Low D-dimer (Unmatched)**	**Other hospitalisation High D-dimer (Matched)**	**Other hospitalisation Low D-dimer (Matched)**
Total Number	16099	12148	11129	11129
DEMOGRAPHICS				
Age; mean (SD)	47.4 (23.0)	49.2 (20.1)	49.1 (22.1)	48.8 (20.5)
Sex; n (%)				
Female	8607 (53.5)	6799 (56.0)	6109 (54.9)	6112 (54.9)
Male	7459 (46.3)	5349 (44.0)	5020 (45.1)	5017 (45.1)
Other	33 (0.2)	0 (0.0)	0 (0.0)	0 (0.0)
Race; n (%)				
White	9158 (56.9)	7702 (63.4)	6902 (62.0)	6919 (62.2)
Black or African American	4133 (25.7)	2511 (20.7)	2446 (22.0)	2417 (21.7)
Asian	415 (2.6)	208 (1.7)	204 (1.8)	204 (1.8)
American Indian or Alaska Native	136 (0.8)	120 (1.0)	108 (1.0)	107 (1.0)
Native Hawaiian or Other Pacific Islander	42 (0.3)	31 (0.3)	30 (0.3)	30 (0.3)
Unknown	2215 (13.8)	1576 (13.0)	1439 (12.9)	1452 (13.0)
Ethnicity; n (%)				
Hispanic or Latino	1826 (11.3)	1284 (10.6)	1138 (10.2)	1209 (10.9)
Not Hispanic or Latino	12414 (77.1)	9872 (81.3)	9055 (81.4)	8945 (80.4)
Unknown	1859 (11.5)	992 (8.2)	936 (8.4)	975 (8.8)
Problems related to housing and economic circumstances	320 (2.0)	359 (3.0)	271 (2.4)	292 (2.6)
COMORBIDITIES				
Overweight and obesity	3336 (20.7)	3357 (27.6)	2765 (24.8)	2769 (24.9)
Hypertensive disease; n (%)				
Hypertensive diseases	7854 (48.8)	6569 (54.1)	5813 (52.2)	5774 (51.9)
Hypertensive diseases (deprecated 2018)	7838 (48.7)	6558 (54.0)	5800 (52.1)	5764 (51.8)
Diabetes mellitus; n (%)				
Type 1 diabetes mellitus	868 (5.4)	919 (7.6)	725 (6.5)	729 (6.5)
Type 2 diabetes mellitus	3806 (23.6)	3519 (29.0)	3022 (27.2)	2971 (26.7)
Chronic lower respiratory diseases; n (%)				
Asthma	2064 (12.8)	2221 (18.3)	1751 (15.7)	1777 (16.0)
Bronchiectasis	192 (1.2)	192 (1.6)	154 (1.4)	156 (1.4)
Bronchitis, not specified as acute or chronic	824 (5.1)	976 (8.0)	739 (6.6)	742 (6.7)
Emphysema	549 (3.4)	495 (4.1)	419 (3.8)	430 (3.9)
Other chronic obstructive pulmonary disease	1946 (12.1)	1895 (15.6)	1553 (14.0)	1595 (14.3)
Simple and mucopurulent chronic bronchitis	119 (0.7)	114 (0.9)	88 (0.8)	91 (0.8)
Unspecified chronic bronchitis	149 (0.9)	169 (1.4)	124 (1.1)	128 (1.1)
Nicotine dependence	2849 (17.7)	2850 (23.5)	2338 (21.0)	2339 (21.0)
Psychiatric comorbidities; n (%)				
Anxiety disorders	3835 (23.8)	3811 (31.4)	3133 (28.2)	3154 (28.3)
Substance misuse	3979 (24.7)	3721 (30.6)	3129 (28.1)	3109 (27.9)
Mood disorders	3634 (22.6)	3616 (29.8)	2929 (26.3)	2972 (26.7)
Psychotic disorders	673 (4.2)	599 (4.9)	491 (4.4)	504 (4.5)
Heart disease; n (%)				
Ischemic heart diseases	3615 (22.5)	3411 (28.1)	2888 (25.9)	2878 (25.9)
Other forms of heart disease	7297 (45.3)	5864 (48.3)	5244 (47.1)	5227 (47.0)
Chronic kidney diseases; n (%)				

Chronic kidney disease (CKD)	2872 (17.8)	1971 (16.2)	1846 (16.6)	1829 (16.4)
Hypertensive chronic kidney disease	1851 (11.5)	1242 (10.2)	1194 (10.7)	1165 (10.5)
Chronic liver diseases; n (%)				
Alcoholic liver disease	437 (2.7)	171 (1.4)	181 (1.6)	169 (1.5)
Chronic hepatitis, not elsewhere classified	74 (0.5)	57 (0.5)	49 (0.4)	48 (0.4)
Chronic passive congestion of liver	362 (2.2)	257 (2.1)	239 (2.1)	236 (2.1)
Fatty (change of) liver, not elsewhere classified	825 (5.1)	795 (6.5)	625 (5.6)	658 (5.9)
Fibrosis and cirrhosis of liver	756 (4.7)	362 (3.0)	365 (3.3)	346 (3.1)
Hepatic failure, not elsewhere classified	778 (4.8)	228 (1.9)	228 (2.0)	227 (2.0)
Other specified diseases of liver	974 (6.0)	765 (6.3)	653 (5.9)	664 (6.0)
Portal hypertension	464 (2.9)	162 (1.3)	162 (1.5)	158 (1.4)
Cerebral infarction	1322 (8.2)	1079 (8.9)	962 (8.6)	963 (8.7)
Dementia; n (%)				
Alzheimer's disease	61 (0.4)	26 (0.2)	21 (0.2)	26 (0.2)
Dementia in other diseases classified elsewhere	86 (0.5)	53 (0.4)	47 (0.4)	46 (0.4)
Dementia with Lewy bodies	10 (0.06)	12 (0.1)	10 (0.09)	10 (0.09)
Frontotemporal dementia	10 (0.06)	10 (0.08)	10 (0.09)	10 (0.09)
Unspecified dementia	244 (1.5)	186 (1.5)	164 (1.5)	170 (1.5)
Vascular dementia	55 (0.3)	38 (0.3)	31 (0.3)	37 (0.3)
Neoplasms; n (%)				
Malignant neoplasms of lymphoid, hematopoietic and related tissue	1469 (9.1)	850 (7.0)	802 (7.2)	831 (7.5)
Neoplasms (any including benign)	4563 (28.3)	3663 (30.2)	3211 (28.9)	3211 (28.9)
Organ transplant; n (%)				
Liver Transplantation Procedures	94 (0.6)	14 (0.1)	18 (0.2)	14 (0.1)
Renal Transplantation Procedures	71 (0.4)	31 (0.3)	31 (0.3)	31 (0.3)
Psoriasis	220 (1.4)	207 (1.7)	158 (1.4)	164 (1.5)
Rheumatoid arthritis; n (%)				
Other rheumatoid arthritis	658 (4.1)	529 (4.4)	459 (4.1)	457 (4.1)
Rheumatoid arthritis with rheumatoid factor	93 (0.6)	69 (0.6)	65 (0.6)	59 (0.5)
Systemic lupus erythematosus (SLE)	733 (4.6)	441 (3.6)	402 (3.6)	417 (3.7)
Disorders involving the immune mechanism	1357 (8.4)	953 (7.8)	872 (7.8)	860 (7.7)
MEDICATIONS				
COVID-19 vaccine; n (%)				
Pfizer	0 (0.0)	0 (0.0)	0 (0.0)	0 (0.0)
Moderna	0 (0.0)	0 (0.0)	0 (0.0)	0 (0.0)
Janssen	0 (0.0)	0 (0.0)	0 (0.0)	0 (0.0)
Not specified	0 (0.0)	0 (0.0)	0 (0.0)	0 (0.0)
Antidepressants; n (%)				
Any	6441 (40.0)	5260 (43.3)	4623 (41.5)	4641 (41.7)
Fluvoxamine	21 (0.1)	24 (0.2)	17 (0.2)	19 (0.2)
Lithium	96 (0.6)	98 (0.8)	77 (0.7)	82 (0.7)
Antipsychotics; n (%)				
Any	2908 (18.1)	2110 (17.4)	1857 (16.7)	1904 (17.1)
Clozapine	21 (0.1)	24 (0.2)	18 (0.2)	21 (0.2)

References

1. Evans, R. A. *et al.* Physical, cognitive, and mental health impacts of COVID-19 after hospitalisation (PHOSP-COVID): a UK multicentre, prospective cohort study. *Lancet Respir Med* **9**, 1275–1287 (2021).
