# Supplementary material for: Acute blood biomarker profiles predict cognitive deficits 6 and 12 months after COVID-19 hospitalization
Source: Nat Med. 2023 Aug 31;29(10):2498–508. doi: 10.1038/s41591-023-02525-y (PMC10579097; doi:10.1038/s41591-023-02525-y)
Supplement: Supplementary file 1 [file 41591_2023_2525_MOESM2_ESM.pdf]

nature portfolio

Corresponding author(s): Maxime Taquet

Last updated by author(s): Jul 19, 2023

Reporting Summary

Nature Portfolio wishes to improve the reproducibility of the work that we publish. This form provides structure for consistency and transparency in reporting. For further information on Nature Portfolio policies, see our Editorial Policies and the Editorial Policy Checklist.

Statistics

For all statistical analyses, confirm that the following items are present in the figure legend, table legend, main text, or Methods section.

n/a	Confirmed	
☐	☒	The exact sample size (n) for each experimental group/condition, given as a discrete number and unit of measurement
☐	☒	A statement on whether measurements were taken from distinct samples or whether the same sample was measured repeatedly
☐	☒	The statistical test(s) used AND whether they are one- or two-sided<br>*Only common tests should be described solely by name; describe more complex techniques in the Methods section.*
☐	☒	A description of all covariates tested
☐	☒	A description of any assumptions or corrections, such as tests of normality and adjustment for multiple comparisons
☐	☒	A full description of the statistical parameters including central tendency (e.g. means) or other basic estimates (e.g. regression coefficient) AND variation (e.g. standard deviation) or associated estimates of uncertainty (e.g. confidence intervals)
☐	☒	For null hypothesis testing, the test statistic (e.g. F, t, r) with confidence intervals, effect sizes, degrees of freedom and P value noted<br>*Give P values as exact values whenever suitable.*
☐	☒	For Bayesian analysis, information on the choice of priors and Markov chain Monte Carlo settings
☐	☒	For hierarchical and complex designs, identification of the appropriate level for tests and full reporting of outcomes
☐	☒	Estimates of effect sizes (e.g. Cohen's d, Pearson's r), indicating how they were calculated

Our web collection on statistics for biologists contains articles on many of the points above.

Software and code

Policy information about availability of computer code

Data collection	No software was used to collect data of the PHOSP-COVID study.<br>Data for the electronic health records data were collected and aggregated within TriNetX internal platform.
Data analysis	R programming language; version 4.2.0 and the following R packages: mice (version 3.14.0), mediation (version 3.14.0), lavaan (version 0.6.14)

For manuscripts utilizing custom algorithms or software that are central to the research but not yet described in published literature, software must be made available to editors and reviewers. We strongly encourage code deposition in a community repository (e.g. GitHub). See the Nature Portfolio guidelines for submitting code & software for further information.

Data

Policy information about availability of data

All manuscripts must include a data availability statement. This statement should provide the following information, where applicable:

- Accession codes, unique identifiers, or web links for publicly available datasets
- A description of any restrictions on data availability
- For clinical datasets or third party data, please ensure that the statement adheres to our policy

For the PHOSP-COVID data, the protocol, consent form, definition and derivation of clinical characteristics and outcomes, training materials, regulatory documents, requests for data access and other relevant study materials are available online at https://www.phosp.org.

For the TriNetX data, the system returned the results of these analyses as csv files, which we downloaded and archived. Aggregate data, as presented in this article, can be freely accessed at https://osf.io/kzhfs/. This study had no special privileges. Inclusion criteria specified in the Methods and Supplementary Material would allow other researchers to identify similar cohorts of patients as we used here for these analyses; however, TriNetX is a live platform with new data being added daily so exact counts will vary. To gain access to the data, a request can be made to TriNetX (join@trinetx.com), but costs might be incurred, and a data sharing agreement would be necessary.

Human research participants

Policy information about studies involving human research participants and Sex and Gender in Research.

Reporting on sex and gender	In this study we only collected information on participants' sex (biological attribute) and throughout the manuscript we referred to it by its appropriate name. Findings presented in this manuscript apply to all participants regardless of the reported sex. This variable was determined on self-reporting. The sex was split into 2 categories: Female and Male. Overall, the study consisted of 673 female participants and 1060 male participants, with the remaining 104 participants with a missing record. The study did not collect participants' gender as it was assumed not to influence COVID-19 severity and the resulting COVID-19 induced cognitive deficit.
Population characteristics	1837 human research participants, with a mean (SD) age of 57.9 (12.4); 57.7% reported their sex as "Male", 36.6% as "Female" and 5.7% provided no information of their reported sex. Participants mostly white (75.4%) or Asian (11.8%), broadly uniformly split across categories of the highest attained education level or reported household income.<br>Mostly married (56.3%) and reporting English as their first language (80%). Prevalence of cardiovascular condition was 45%, diabetes was 19.9%, respiratory condition was 27.6%, rheumatological condition was 15.5%, psychiatric condition was 18.1% and a gastrointestinal condition was 21.3%.
Recruitment	The researchers collected data from clinic visits and from routine health records of all participants. This included signs and symptoms, medication, physical test results, questionnaire answers, laboratory test results and imaging. In a subset of participants, the researchers undertook additional research tests and obtained samples (for example, blood) for research experiments. Some participants were asked to take part in additional studies. The baseline characteristics of the PHOSP-COVID cohort appear largely representative of the general population of individuals hospitalised with COVID-19. In addition, the baseline characteristics of participants recruited in the Tier 2 sub-study of PHOSP-COVID (used here) were largely similar to those of the larger cohort recruited in Tier 1 (which only involved remote data collection based on their health records). This suggests that none of the baseline characteristics recorded were important determinants of self-selection in the study. However, it is possible that other non-recorded characteristics (e.g. general attitude towards science and medicine, genetic characteristics, etc) might have influenced self-selection. In this regard, the replication of the findings using real-world data is important as the latter is not subject to any form of self-selection.
Ethics oversight	Approved 14/07/2020, Yorkshire & The Humber-Leeds West Research Ethics Committee (NHSBT Newcastle Blood Donor Centre, Holland Drive, Newcastle upon Tyne, NE2 4NQ, UK; +44 (0)207 972 2504, +44 (0)207 104 8088, +44 (0)207 104 8018; leedswest.rec@hra.nhs.uk), REC ref: 20/YH/0225

Note that full information on the approval of the study protocol must also be provided in the manuscript.

Field-specific reporting

Please select the one below that is the best fit for your research. If you are not sure, read the appropriate sections before making your selection.

☒ Life sciences ☐ Behavioural & social sciences ☐ Ecological, evolutionary & environmental sciences

For a reference copy of the document with all sections, see nature.com/documents/nr-reporting-summary-flat.pdf

Life sciences study design

All studies must disclose on these points even when the disclosure is negative.

Sample size	All participants of the PHOSP-COVID Tier 2 study were eligible. We applied some exclusion criteria (see below) to make sure that they had some measurements for the variable of interest.
Data exclusions	To be part of the PHOSP study, individuals were excluded if they met any of the following exclusion criteria:<br>1. Confirmed diagnosis of a pathogen unrelated to the objectives of this study and no indication or likelihood of co-infection with a relevant pathogen<br>2. Attendance at an A&E or emergency department only<br>3. Refusal by participant, parent or appropriate representative<br>4. Other life-limiting illness with life expectancy <6 months such as disseminated malignancy<br><br>In addition, among those who were part of the PHOSP Tier 2 study, those who were included in this sub-study could not meet any of the following exclusion criteria:<br>- missing records for all of the six key biomarkers considered in the study;<br>- missing value for all of the MoCA components.

Replication	Replication of the main study finding was performed on a separate population using electronic health records (EHR) data from TriNetX Analytics, a large-scale EHR network covering over 90 million patients predominantly in the USA. Within this dataset, all individuals hospitalised with COVID-19 were identified and divided into dichotomous subgroups based on the measurements of key biomarkers - fibrinogen, CRP and D-dimer.<br><br>Within the TriNetX platform we were able to successfully replicate the main findings identified in the primary analysis.<br><br>The first dimension of covariation was replicated by comparing TriNetX sub-cohorts with high fibrinogen and normal CRP to subjects with low fibrinogen and normal CRP. In this comparison, fibrinogen level was found to be statistically significantly associated with post-COVID-19 cognitive deficits.<br><br>The second dimension was replicated by comparing a cohort with high D-dimer and normal CRP to cohort with low D-dimer and normal CRP at the time of the acute infection with SARS-CoV-2. In this comparison, D-dimer levels were found to be statistically significantly associated with post-COVID-19 cognitive deficits.
Randomization	For the prospective part of the study, the analysis was adjusted for a range of covariates included in a linear regression model from which residuals were used as input to the analysis. Top and bottom half of the cohorts along dimensions of covariation were note to be well balanced in terms of covariates. For the retrospective part of the study, cohorts were propensity-score matched on a wide range of covariates and appropriate matching was tested using standardised mean difference.
Blinding	Blinding was not relevant for this study as there was no groups which were compared in the primary analysis. Instead, a discovery science approach was used to identify biomarker profiles linked to cognitive profiles.

Reporting for specific materials, systems and methods

We require information from authors about some types of materials, experimental systems and methods used in many studies. Here, indicate whether each material, system or method listed is relevant to your study. If you are not sure if a list item applies to your research, read the appropriate section before selecting a response.

Materials & experimental systems

n/a	Involved in the study
☒	☐ Antibodies
☒	☐ Eukaryotic cell lines
☒	☐ Palaeontology and archaeology
☒	☐ Animals and other organisms
☐	☒ Clinical data
☒	☐ Dual use research of concern

Methods

n/a	Involved in the study
☒	☐ ChIP-seq
☒	☐ Flow cytometry
☒	☐ MRI-based neuroimaging

Clinical data

Policy information about clinical studies

All manuscripts should comply with the ICMJE guidelines for publication of clinical research and a completed CONSORT checklist must be included with all submissions.

Clinical trial registration	IRAS number<br>285439<br><br>Protocol/serial number<br>CPMS 46443, IRAS 285439
Study protocol	Study protocol is available under the following link<br>https://www.phosp.org/document/102/<br>Accessing the document requires a password.
Data collection	Dates of recruitment: August 2020 and March 2022 of patients hospitalised with COVID-19 between January 29, 2020 and November 20, 2021. Data collection at 6 and 12 months post-hospitalisation for each participant.<br><br>Countries of recruitment: England, Northern Ireland, Scotland, Wales<br><br>Trial participating centre<br>Queen Elizabeth Hospital<br>Heritage Building<br>University Hospitals of Birmingham NHS Trust<br>Mindelsohn Way<br>Edgbaston<br>Birmingham<br>B15 2TH<br>United Kingdom

Trial participating centre
Hull Royal Infirmary
Hull University Teaching Hospitals NHS Trust
Anlaby Rd
Hull
HU3 2JZ
United Kingdom

Trial participating centre
Bradford Royal Infirmary
Bradford Teaching Hospitals NHS Foundation Trust
Duckworth Ln
Bradford
BD9 6RJ
United Kingdom

Trial participating centre
Southmead Hospital
North Bristol NHS Trust
Southmead Rd
Bristol
BS10 5NB
United Kingdom

Trial participating centre
Fulbourn Hospital
Cambridgeshire and Peterborough NHS Foundation Trust
Elizabeth House
Cambridge
CB21 5EF
United Kingdom

Trial participating centre
Royal Papworth Hospital
Royal Papworth Hospital NHS Foundation Trust
Papworth Rd
Trumpington
Cambridge
CB2 0AY
United Kingdom

Trial participating centre
Leeds General Infirmary
The Leeds Teaching Hospitals NHS Trust
Great George St
Leeds
LS1 3EX
United Kingdom

Trial participating centre
Glenfield Hospital
University Hospitals of Leicester NHS Trust
Groby Road
Leicester
LE3 9QP
United Kingdom

Trial participating centre
Royal Liverpool Hospital
Liverpool University Hospitals NHS Foundation Trust
Prescot St
Liverpool
L7 8XP
United Kingdom

Trial participating centre
St Mary's Hospital
Imperial College Healthcare NHS Trust
The Bays
South Wharf Road
London
W2 1NY
United Kingdom

Trial participating centre
Chelsea & Westminster Hospital

Chelsea & Westminster Hospital NHS Trust
369 Fulham Rd
Chelsea
London
SW10 9NH
United Kingdom

Trial participating centre
Northwick Park Hospital
London North West University Healthcare NHS Trust
Central Middlesex
Ealing Hospital
London
HA1 3UJ
United Kingdom

Trial participating centre
Mount Vernon Hospital
The Hillingdon Hospitals NHS Foundation Trust
Rickmansworth Road
Northwood
London
HA6 2RN
United Kingdom

Trial participating centre
Royal Brompton & Harefield Trust
Royal Brompton Hospital
1 Manresa Rd
Chelsea
London
SW3 6LR
United Kingdom

Trial participating centre
St Thomas' Hospital
Guy's and St Thomas' NHS Foundation Trust
Westminster Bridge Rd
London
SE1 7EH
United Kingdom

Trial participating centre
King's College Hospital
King's College Hospital NHS Foundation Trust
Denmark Hill
Brixton
London
SE5 9RS
United Kingdom

Trial participating centre
The Royal Hospital
Barts Health NHS Trust
Whitechapel Rd
London
E1 1BB
United Kingdom

Trial participating centre
University College London Hospital
University College London Hospitals NHS Foundation Trust
235 Euston Road
Bloomsbury
London
NW1 2BU
United Kingdom

Trial participating centre
The Whittington Hospital
Whittington Health NHS Trust
Magdala Ave
London
N19 5NF
United Kingdom

Trial participating centre
Royal Free Hospital
Royal Free London NHS Foundation Trust
17 Lyndhurst Gardens
Hampstead
London
NW3 5NU
United Kingdom

Trial participating centre
North Middlesex University Hospital
North Middlesex University Hospital NHS Trust
Sterling Way
London
N18 1QX
United Kingdom

Trial participating centre
St George's Hospital
St George's University Hospitals NHS Foundation Trust
Blackshaw Road
Tooting
London
SW17 0QT
United Kingdom

Trial participating centre
Manchester Royal Infirmary
Manchester University NHS Foundation Trust
Cobbett House
Oxford Road
Manchester
M13 9WL
United Kingdom

Trial participating centre
Salford Royal Hospital
Salford Royal NHS Foundation Trust
Stott Ln
Salford
M6 8HD
United Kingdom

Trial participating centre
Freeman Hospital
Newcastle Upon Tyne Hospitals NHS Foundation Trust
Freeman Road
High Heaton
Newcastle-upon-Tyne
NE7 7DN
United Kingdom

Trial participating centre
Belfast City Hospital
Belfast Health and Social Care Trust
A Floor
Lisburn Road
Belfast
BT9 7AB
United Kingdom

Trial participating centre
Nottingham City Hospital
Nottingham University Hospitals NHS Trust
Hucknall Road
Nottingham
NG5 1PB
United Kingdom

Trial participating centre
John Radcliffe Hospital
Oxford University Hospitals NHS Foundation Trust
Headley Way
Headington
Oxford
OX3 9DU

United Kingdom

Trial participating centre
NHS Grampian
Summerfield House
2 Eday Road
Aberdeen
AB15 6RE
United Kingdom

Trial participating centre
NHS Dumfries and Galloway
21-22 High St
Moffat
DG10 9HL
United Kingdom

Trial participating centre
NHS Tayside
230 Clepington Rd
Dundee
DD2 1GZ
United Kingdom

Trial participating centre
NHS Fife
Hayfield House
Hayfield Rd
Kirkcaldy
KY2 5AH
United Kingdom

Trial participating centre
NHS Forth Valley
Stirling Rd
Larbert
FK5 4WR
United Kingdom

Trial participating centre
NHS Highland
Inverness Retail and Business Park
John Dewar Building
Highlander Way
Inverness
IV2 7GE
United Kingdom

Trial participating centre
NHS Greater Glasgow and Clyde
1055 Great Western Road
Glasgow
G12 0XH
United Kingdom

Trial participating centre
NHS Lothian
Search Results
Morningside Pl
Edinburgh
EH10 5HF
United Kingdom

Trial participating centre
Royal Hallamshire Hospital
Sheffield Teaching Hospitals NHS Foundation Trust
Glossop Rd
Broomhall
Sheffield
S10 2JF
United Kingdom

Trial participating centre
Southampton General Hospital,
University Hospital Southampton NHS Foundation Trust
Tremona Rd

Southampton
SO16 6YD
United Kingdom

Trial participating centre
Hywel Dda University Health Board
Ystwyth Building
St Davids Park
Jobswell Road
Carmarthen
SA31 3BB
United Kingdom

Trial participating centre
Swansea Bay University Health Board
1 Talbot Gateway
Baglan Energy Park
Baglan
Port Talbot
SA12 7BR
United Kingdom

Trial participating centre
Cardiff & Vale University Health Board
Heath Park
Cardiff
CF14 4XW
United Kingdom

Trial participating centre
Aneurin Bevan University Health Board
Ringland Circle
Newport
NP19 9PS
United Kingdom

Trial participating centre
Betsi Cadwaladr University Health Board
Glan Clwyd Hospital
Sarn Ln
Bodelwyddan
Rhyl
LL18 5UJ
United Kingdom

Trial participating centre
Lanarkshire Primary Care NHS Trust
East Kilbride
Glasgow
G75 8NH
United Kingdom

Outcomes

Primary outcome measures:

1. The Montreal Cognitive Assessment (MoCA) measured at 6 months following hospitalisation for COVID-19. This consists of 7 distinct items covering several cognitive domains, including short-term memory, visuospatial abilities, abstract reasoning, orientation in time and space, language fluency, sustained attention, and executive function.

2. Cognitive Patient Symptom Questionnaire (C-PSQ) measured at 6 months, following hospitalisation for COVID-19. The questionnaire consisted of 7 items relating to subjects' perceived difficulty in communicating, remembering, concentrating, recalling, and experiencing episodes of confusion or slow thinking.

Secondary outcome measures:

1. Occupational change anytime within 6 months of hospital discharge following COVID-19 hospitalisation.

2. Occupational change anytime 6 to 12 months after hospital discharge following COVID-19 hospitalisation.

3. Perceived difficulty working anytime within 6 months of hospital discharge following COVID-19 hospitalisation.

4. Perceived difficulty working 6 to 12 months after hospital discharge following COVID-19 hospitalisation.

Difficulty working was assessed based on a simple question "Has your (COVID-19) illness affected your ability to do your usual work?"

Occupational change was based on participants reporting a change in their main occupation which occurred between before and after their COVID-19 illness. We only recorded a positive outcome for those who reported a change in occupation and for whom the occupation after COVID-19 was not "Working full-time". Similarly, for participants who reported a change in occupation and for whom there was no information on their occupation before and after their COVID-19 illness, we reported the change in occupation as ‘unknown’.
